# Supplementary material for: Prognostic factors and prediction models for hospitalisation and all-cause mortality in adults presenting to primary care with a lower respiratory tract infection: a systematic review
Source: BMJ Open. 2024 Mar 23;14(3):e075475. doi: 10.1136/bmjopen-2023-075475 (PMC10961536; doi:10.1136/bmjopen-2023-075475)
Supplement: Supplementary data [file bmjopen-2023-075475supp001.pdf]

Supplementary data

Table S1: search strategy

Search date: 27 June 2022		
**Pubmed**	**Embase**	**The Cochrane Library**
((((("RTI"[Title/Abstract] OR "respiratory tract infection*"[Title/Abstract] OR "respiratory infection*"[Title/Abstract] OR "pneumonia*"[Title/Abstract] OR "bronchitis"[Title/Abstract] OR "lung infection*"[Title/Abstract] OR "chest infection*"[Title/Abstract]) OR ("Respiratory Tract Infections"[Mesh])) AND (((Validat* OR Predict*[Title] OR Rule*) OR (Predict* AND (Outcome* OR Risk* OR Model*)) OR ((History OR Variable* OR Criteria OR Scor* OR Characteristic* OR Finding* OR Factor*) AND (Predict* OR Model* OR Decision* OR Identif* OR Prognos*)) OR (Decision* AND (Model* OR Clinical* OR Logistic Models)) OR (Prognostic AND (History OR Variable* OR Criteria OR Scor* OR Characteristic* OR Finding* OR Factor* OR Model*))) OR (‘’Stratification’’ OR ‘’ROC Curve’’[Mesh] OR ‘’Discrimination’’ OR ‘’Discriminate’’ OR ‘’c-statistic’’ OR ‘’c statistic’’ OR ‘’Area under the curve’’ OR ‘’AUC’’ OR ‘’Calibration’’ OR ‘’Indices’’ OR ‘’Algorithm’’ OR ‘’Multivariable’’))) AND (("hospital*"[Title/Abstract] OR "complicat*"[Title/Abstract] OR "morbidity"[Title/Abstract] OR "death"[Title/Abstract] OR "mortality"[Title/Abstract] OR	(('rti':ti,ab,kw OR 'respiratory tract infection*':ti,ab,kw OR 'respiratory infection*':ti,ab,kw OR 'pneumonia*':ti,ab,kw OR 'bronchitis':ti,ab,kw OR 'lung infection*':ti,ab,kw OR 'chest infection*':ti,ab,kw) OR ('respiratory tract infection'/exp OR 'respiratory tract inflammation'/exp)) AND ((validat* OR predict*:ti OR rule* OR (predict* AND (outcome* OR risk* OR model*)) OR ((history OR variable* OR criteria OR scor* OR characteristic* OR finding* OR factor*) AND (predict* OR model* OR decision* OR identif* OR prognos*)) OR (decision* AND (model* OR clinical* OR logistic) AND models) OR (prognostic AND (history OR variable* OR criteria OR scor* OR characteristic* OR finding* OR factor* OR model*))) OR ('stratification' OR 'receiver operating characteristic'/exp OR 'discrimination' OR 'discriminate' OR 'c-statistic' OR 'c statistic' OR 'area under the curve' OR 'auc' OR 'calibration' OR 'indices' OR 'algorithm' OR 'multivariable')) AND (('hospital*':ti,ab,kw OR 'complicat*':ti,ab,kw OR 'morbidity':ti,ab,kw OR 'death':ti,ab,kw OR 'mortality':ti,ab,kw OR 'surviv*':ti,ab,kw) OR ('hospitalization'/exp OR 'hospital admission'/exp OR	(RTI OR respiratory tract infection OR respiratory infection OR pneumonia OR bronchitis OR lung infection OR chest infection) AND (hospitalisation OR hospitalization OR hospital OR complication OR complicated OR morbidity OR death OR mortality OR survival OR survive) AND (Primary care OR general practice OR general practitioner OR family practice OR GP) in Title Abstract Keyword

"surviv*"[Title/Abstract]) OR ("Mortality"[Mesh] OR "Hospitalization"[Mesh] OR "Death"[Mesh]))) AND (("Primary care"[Title/Abstract] OR "general practi*"[Title/Abstract] OR "family practice"[Title/Abstract] OR "GP"[Title/Abstract]) OR ("Primary Health Care"[Mesh] OR "Family Practice"[Mesh] OR "General Practice"[Mesh]))) NOT (("child*"[Title/Abstract] OR "pediatric*"[Title/Abstract] OR "paediatric*"[Title/Abstract] OR "infan*"[Title/Abstract] OR "adolescen*"[Title/Abstract] OR "young*"[Title/Abstract] OR “newborn*”[Title/Abstract]) OR ("Infant"[Mesh] OR "Child"[Mesh] OR "Adolescent"[Mesh] OR "Pediatrics"[Mesh] OR "Young Adult"[Mesh]))	'death'/exp OR 'mortality'/exp)) AND (('primary care':ti,ab,kw OR 'general practi*':ti,ab,kw OR 'family practice':ti,ab,kw OR 'gp':ti,ab,kw) OR ('primary medical care'/exp OR 'primary health care'/exp OR 'general practice'/exp)) NOT (('child*':ti,ab,kw OR 'pediatric*':ti,ab,kw OR 'paediatric*':ti,ab,kw OR 'infan*':ti,ab,kw OR 'adolescen*':ti,ab,kw OR 'young*':ti,ab,kw OR ‘newborn*’:ti,ab,kw) OR ('child'/exp OR 'adolescent'/exp OR 'pediatrics'/exp))	
Filters: none<br>Language restrictions: none<br>Publication date restrictions: none	Filters: none<br>Language restrictions: none<br>Publication date restrictions: none	Filters: none<br>Language restrictions: none<br>Publication date restrictions: none
Records identified: 1592	Records identified: 1392	Records identified: 207

Figure S2: Overall assessment of risk of bias and applicability of included studies

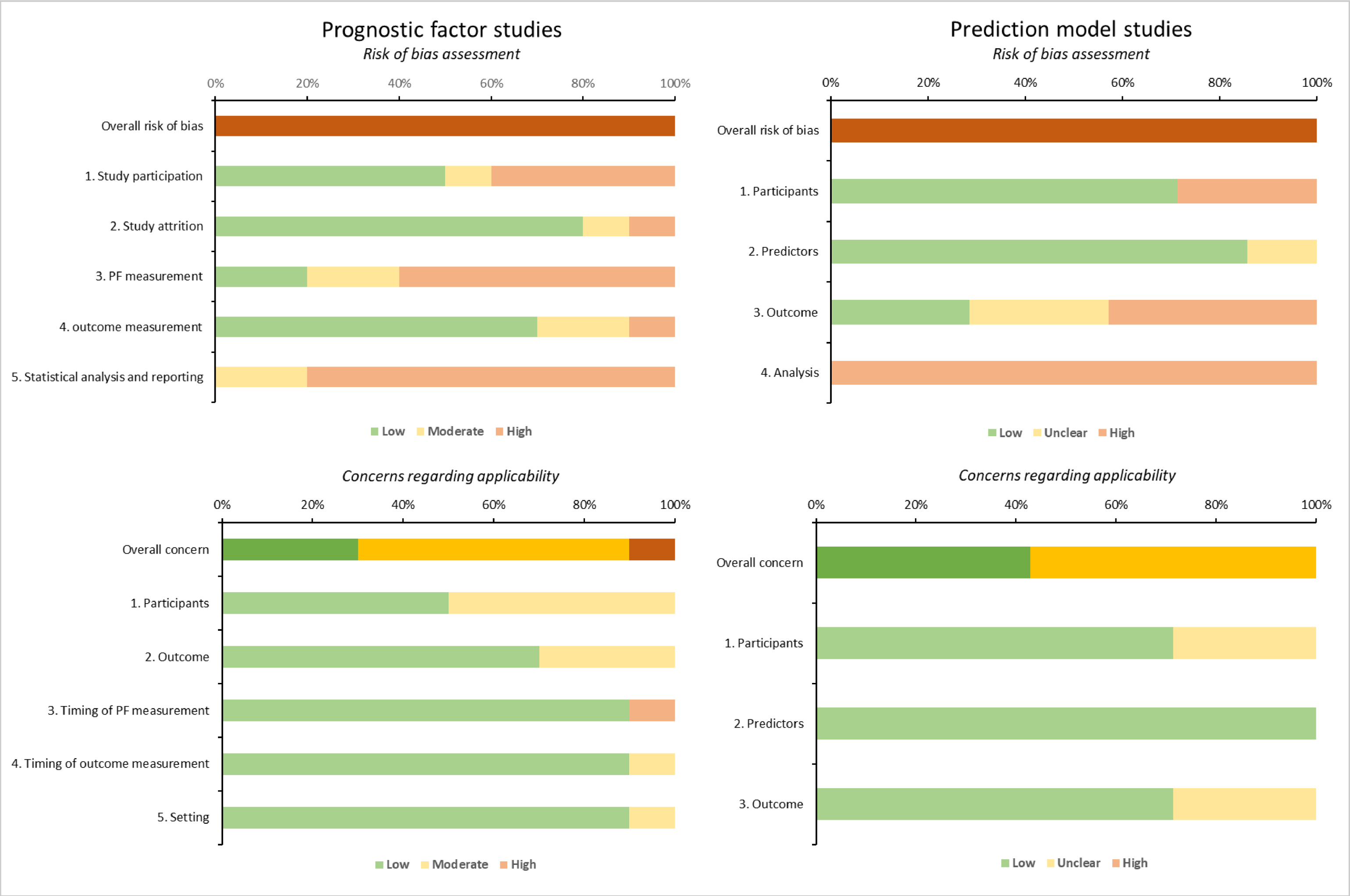

Figure S3: Risk of bias assessment and applicability per individual included study

QUIPS assessment of prognostic factor studies

Study	RoB					Applicability					Overall	
	Study participation	Study attrition	PF measurement	Outcome measurement	Statistical analysis and reporting	Participants	Outcome	Timing of PF measurement	Timing of outcome measurement	Setting	RoB	Applicability
Houston, 1997	±	+	-	+	-	+	+	+	+	±	-	±
Seppä, 2001	-	±	-	+	-	+	+	+	+	+	-	+
Hak, 2005	+	+	±	+	-	+	±	+	+	+	-	±
Myles, 2009	-	+	+	±	-	+	+	+	+	+	-	+
van de Nadort, 2009	+	+	+	±	-	±	+	+	+	+	-	±
Winchester, 2009	+	+	-	+	-	±	±	+	+	+	-	±
Millett, 2015	+	+	±	+	-	+	+	+	+	+	-	+
Moore, 2019	-	+	-	+	±	±	±	+	+	+	-	±
Hamilton, 2021	+	-	-	+	±	±	+	+	+	+	-	±
Martínez-Redondo, 2021	-	+	-	-	-	±	+	-	±	+	-	-

PROBAST assessment of prediction model studies

Study	RoB				Applicability			Overall	
	Participants	Predictors	Outcome	Analysis	Participants	Predictors	Outcome	RoB	Applicability
Bont, 2007	+	+	-	-	+	+	+	-	+
Bont, 2008	-	+	?	-	+	+	+	-	+
Ochoa-Gondar, 2011	+	?	?	-	?	+	+	-	?
Francis, 2012	-	+	-	-	+	+	+	-	+
Ochoa-Gondar, 2013	+	+	+	-	?	+	+	-	?
Bruyndonckx, 2018	+	+	+	-	+	+	?	-	?
Moore, 2019	+	+	+	-	+	+	?	-	?

Figure S4: figure of all PF effect estimates based on univariable analysis

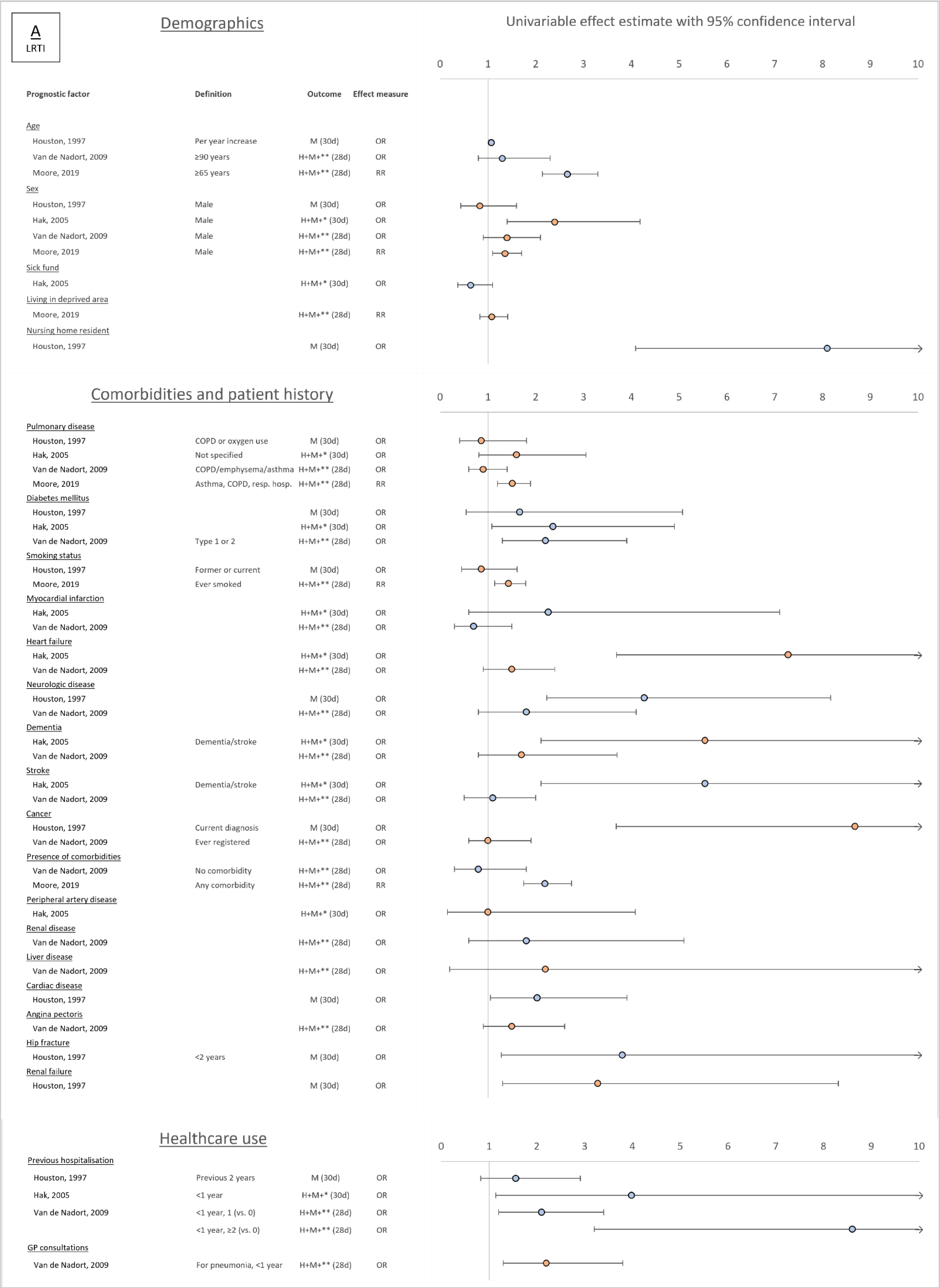

S4, continued

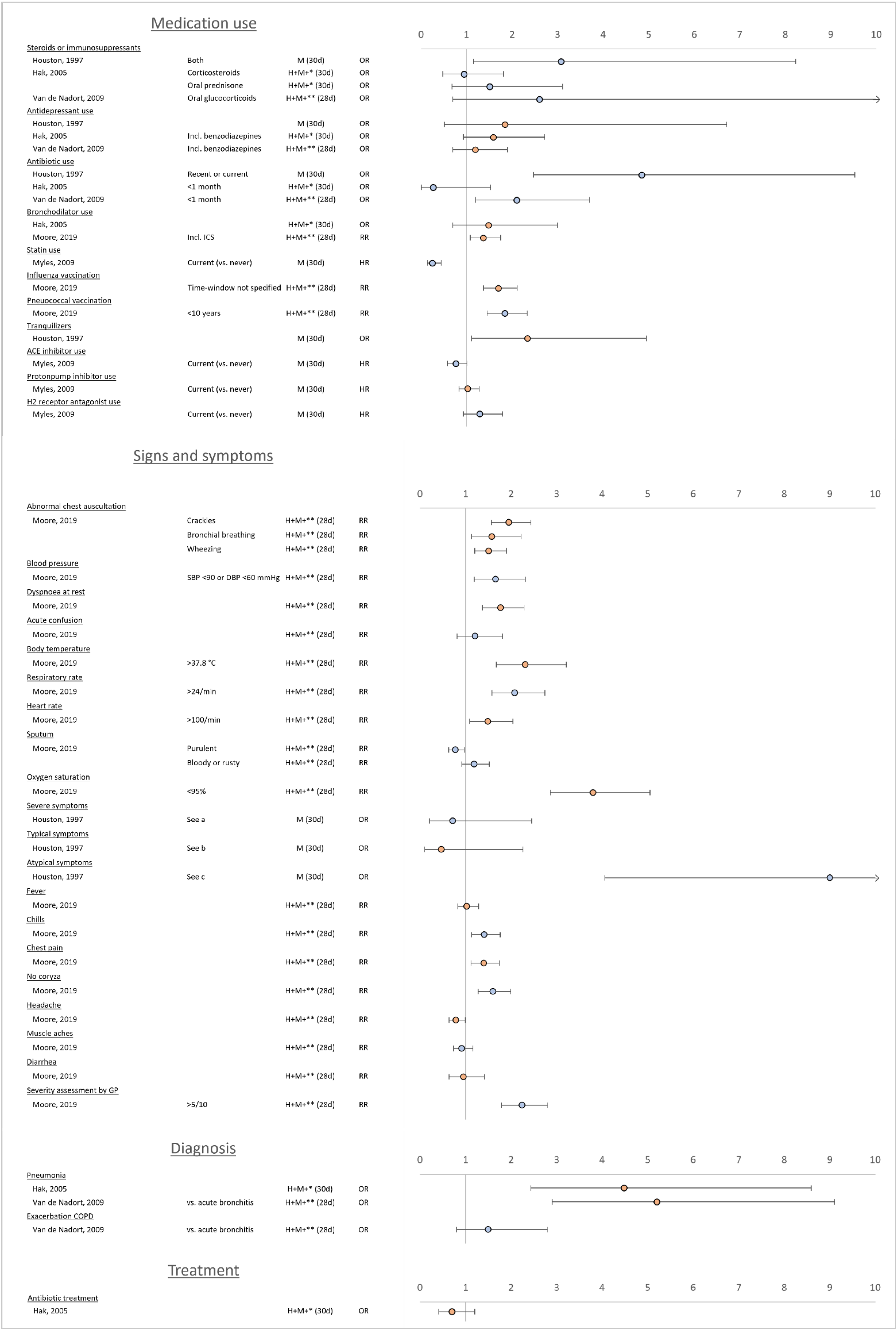

S4, continued

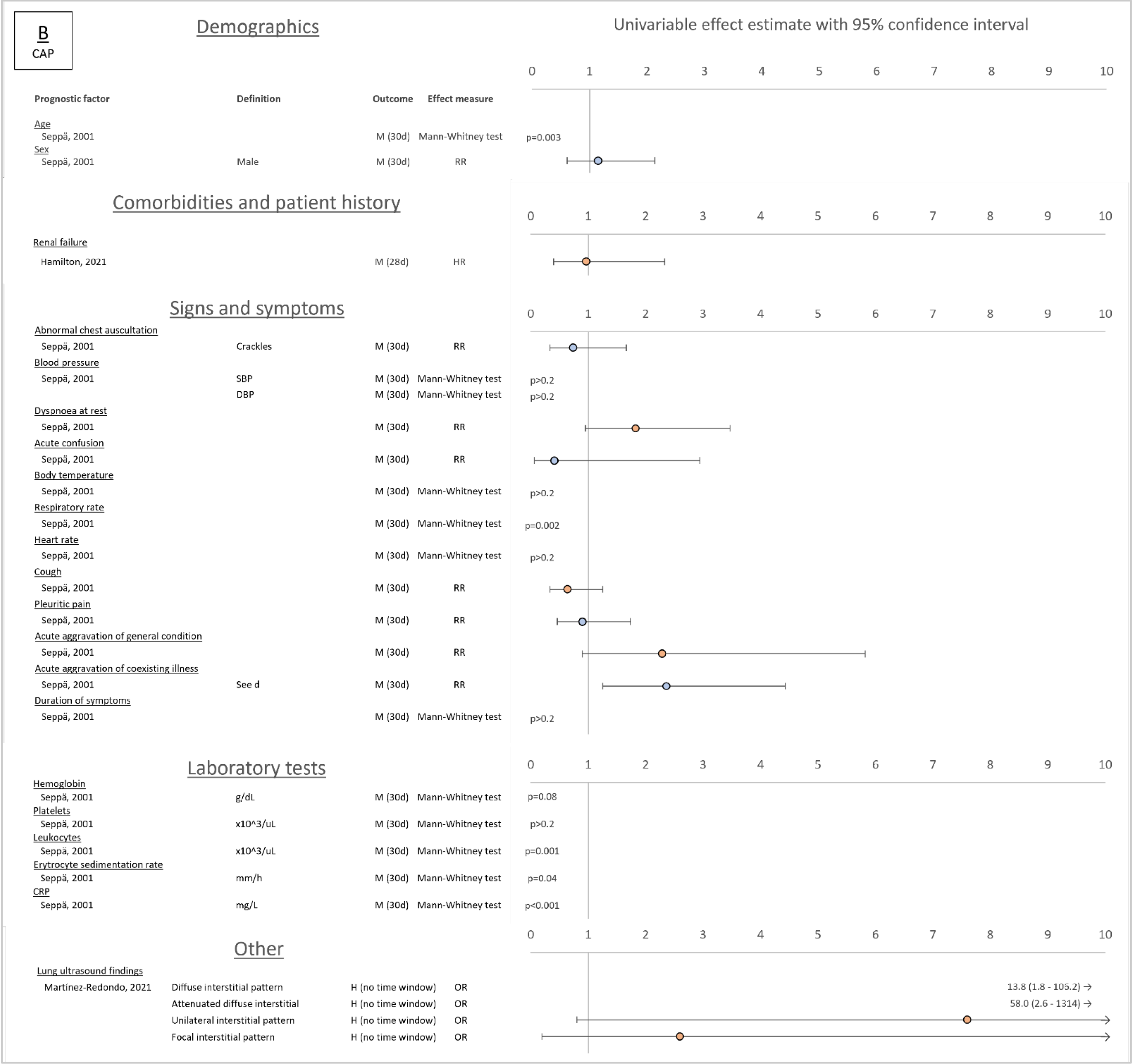

A. Univariable analyses of prognostic factors from studies on LRTI patients. B. Univariable analysis of prognostic factors from studies on pneumonia patients.
* Composite outcome also includes dysregulation of diabetes, stroke, heart failure, MI.
** Composite outcome also includes late onset pneumonia.
a: rigor, hemoptysis, pleuritic pain.
b: chills, cough, congestion, fever, dyspnea, sputum production.
c: poor eating, confusion, lethargy.
d: impairment of glucose balance in diabetic patients, deterioration of congestive heart failure.
Abbreviations: LRTI, lower respiratory tract infection; H, hospitalisation; M, mortality; OR, odds ratio; RR, risk ratio; HR, hazard ratio; COPD, chronic obstructive pulmonary disease; GP, general practitioner; ICS, inhalation corticosteroids; ACE, angiotensin converting enzyme; SBP, systolic blood pressure; DBP, diastolic blood pressure; min, minute; g, gram; dL, deciliter; uL, microliter; mm, millimeter; h, hour; L, liter.

Table S5. Absolute risks according to absence and presence of individual (categorical) prognostic factors

**Study**	Houston, 1997	Seppä, 2001	Hak, 2005	Winchester, 2009		Van de Nadort, 2009	Myles, 2009	Millett, 2015	Moore, 2019	Hamilton, 2021	Martínez-Redondo, 2021
Absolute risks provided or calculable?	No	Yes	Yes	Yes		Yes	Yes	Yes	Yes	No	Yes
Outcome definition	Mortality <30 days	Mortality <30 days	Composite including mortality <30 days	Hosp. <3 months	Mortality <3 months	Hosp. or mortality <30 days	Mortality <30 days	Hosp. <28 days	Composite including hosp. or mortality <30 days	Mortality <28 days	Hosp.
Baseline risk of outcome	10.7 %	4.0 %	14.3%	0.8 %	1.4 %	12.7 %	24.6 %	76.5 %	1.1 %	8.9 %	23.0 %
**Prognostic factor**	**Absolute risk according to prognostic factor (present/absent)**										
Demographics											
Male sex		4.3/3.7 %	20.2/9.5 %	0.8/0.7 %	1.5/1.3 %	14.2/11.8 %		79.5/73.8 %	1.3/0.9 %		
Sick fund			12.3/18.1 %								
Living in deprived area									1.2/1.1 %		
Medical history											
Heart failure			46.7/10.7 %			16.4/10.8 %		79.6/75.5 %			
Dementia/<br>stroke			45.0/12.9 %			17.0/12.4 %<br>12.0/12.8 %		66.8/78.2 %			
Myocardial infarct.			26.7/13.9 %			11.5/12.8 %		83.2/75.4 %			
Periph. arterial dis.			14.3/14.3 %					82.7/75.5 %			
Diabetes mellitus			26.2/13.1 %			23.1/10.8 %		82.6/75.2 %			
Prior hospitalisation			38.5/13.6 %			36.0/10.1 %					
Smoking				0.6/0.8 %	0.9/1.7 %				13.1/0.9 %		
Alcohol				0.7/0.8 %	1.1/2.3 %						
Asthma/COPD			19.7/13.3 %	0.8/0.8 %	0.7/1.5 %	12.3/12.9 %		83.4/71.8 %	1.5/1.0 %		
All. rhinitis				0.7/0.8 %	0.5/1.5 %						
Malignancies						11.9/12.8 %		80.7/75.6 %			
Angina pectoris						18.2/11.4 %		81.5/75.2 %			
Neurological dis.						18.0/12.3 %		73.1/76.8 %			
Renal disease						19.0/12.5 %		88.6/73.8 %			
Liver disease						25.0/12.6 %		85.5/76.4 %			
Connective tissue dis.								81.9/75.9 %			

Peptic ulcer							81.1/76.0 %	
Hemiplegia							76.4/76.5 %	
Leukaemia/lymphoma							85.0/76.2	
Cerebrovascular dis.							74.5/77.2 %	
Terminal illness							67.1/76.9 %	
Medication use								
Antidepressant/ benzodiazepines		18.1/12.2 %			15.4/11.6 %			
Corticosteroids		13.8/14.4 %						
Oral prednisone		19.2/13.6 %			37.5/11.7 %		83.0/75.4 %	
Immunosuppressants							85.3/76.3 %	
ICS							84.7/74.6 %	
Bronchodilator		19.0/13.6 %						1.4/1.0 %
Recent antibiotics		4.5/14.8 %	2.8/0.7 %	3.4/1.4 %	24.2/11.8 %		74.8/77.2 %	
Vaccination								
Influenza			0.9/0.4 %	2.4/0.8 %			76.2/69.7 %	1.5/0.9 %
Pneumococcal			1.0/0.7 %	2.6/1.1 %			81.3/33.6 %	1.8/1.0 %
Statin use						6.8/25.5 %		
ACEi use						18.6/25.1 %		
PPI use						26.1/24.4 %		
H2 antagonist						31.7/24.3 %		
Presenting signs and symptoms								
Cough	3.6/5.6 %							
Dyspnoea	5.3/2.9 %							1.3/0.8 %
Pleuritic pain	3.8/4.2 %							
Confusion	1.8/4.4 %							1.3/1.1 %
Aggravation of								
General condition	3.7/4.7 %							
Coexisting illness	7.6/3.2 %							
Abn. auscultation	3.7/5.0%							
Fever								1.1/1.1 %
Chills								1.4/1.0 %
Chest pain								1.4/1.0 %
No coryza								1.4/0.9 %
Muscle aches								1.1/1.2 %
Diarrhoea								1.1/1.1 %
Purulent sputum								1.0/1.3 %
Bloody sputum								1.6/1.1 %
Severity assessment >5/10								1.7/0.7 %
Respiratory rate >24/min								2.1/1.0 %

Temperature >37.8				2.4/0/1 %	
Pulse >100/min				1.6/1.1 %	
O_2 saturation <95 %				3.5/0.9 %	
SBP<90 or DBP<60 mmHg				1.8/1.1 %	
Crackles				15.7/0.8 %	
Bronchial breathing				1.7/1.1 %	
Wheezing				1.5/1.0 %	
LRTI diagnosis					
Pneumonia	22.6/6.1 %	27.3/8.0 %			
Exacerbation COPD		9.9/14.5 %			
Acute bronchitis		5.8/16.5 %			
Frailty factors					
Recent carer			79.6/76.3 %		
Visual impairment			78.8/75.3 %		
Self-care			79.7/76.4 %		
Anxious/depressed			76.0/76.5 %		
Bedsore/ulcer			59.7/77.0 %		
Mobility issues			79.0/76.3 %		
Tired			74.4/76.6 %		
Low weight/nutrition			75.1/76.7 %		
Incontinence			71.7/77.0		
History of falling			76.4/76.5 %		
Lung ultrasound					
Abnormal					28.2/3.33 %

Abbreviations: NR, not reported; COPD, chronic obstructive pulmonary disease; ICS, inhaled corticosteroids; ACEi, angiotensin converting enzyme inhibitor; PPI, protonpump inhibitor; H2, histamine-2; SBP, systolic blood pressure; DBP, diastolic blood pressure; LRTI, lower respiratory tract infection.

Figure S6: figure of all PF effect estimates based on multivariable effect estimates

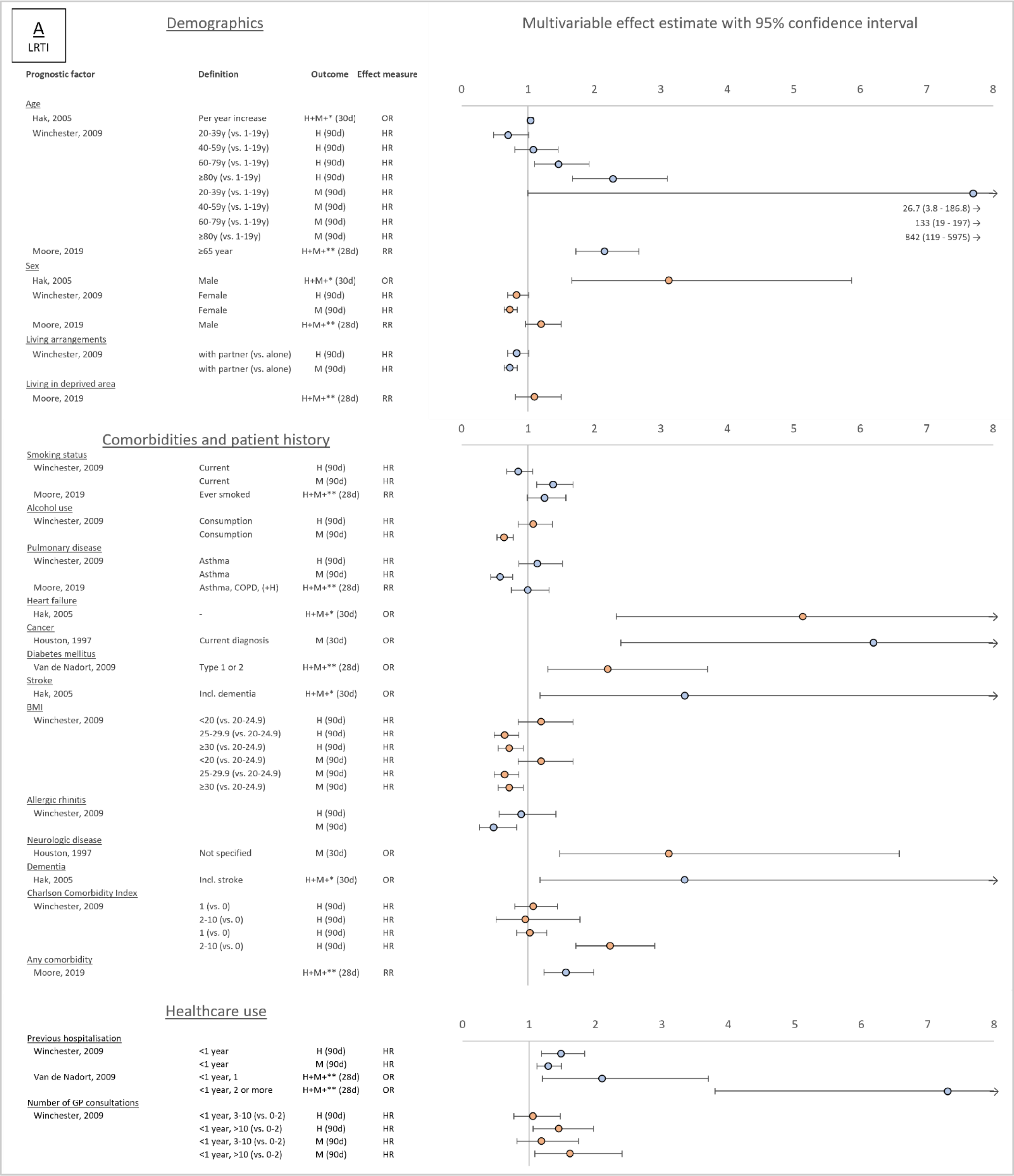

S6, continued

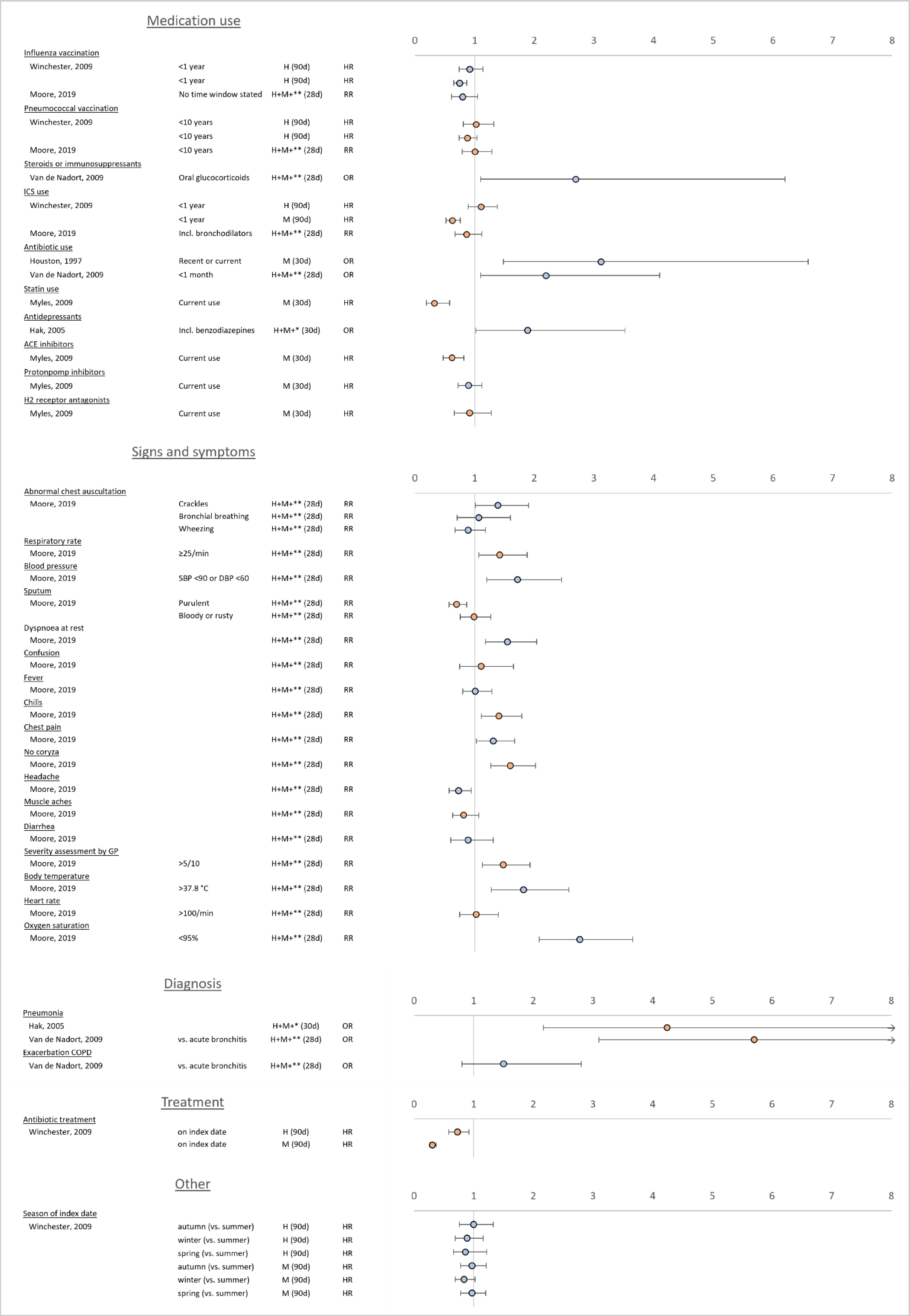

S6, continued

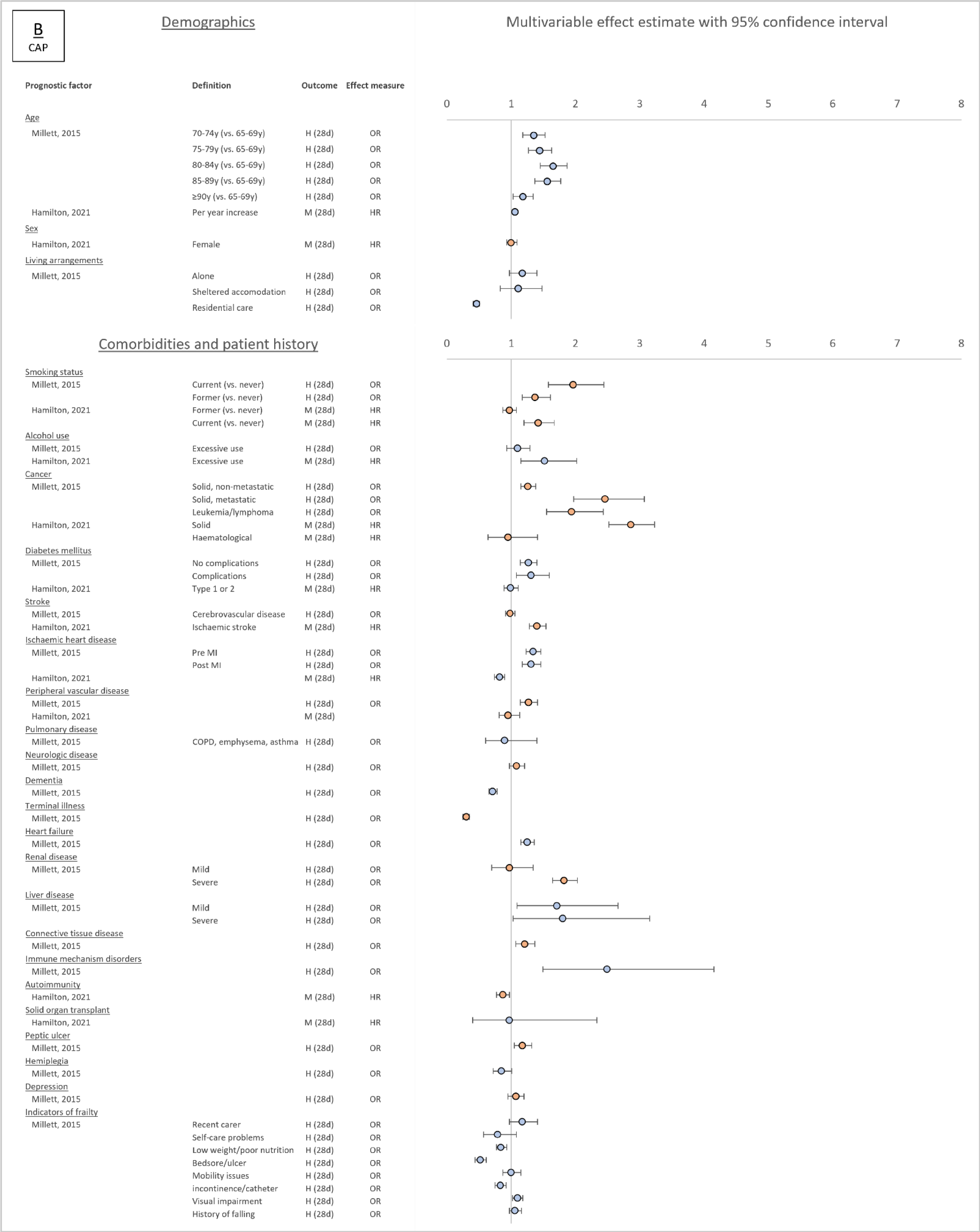

S6, continued

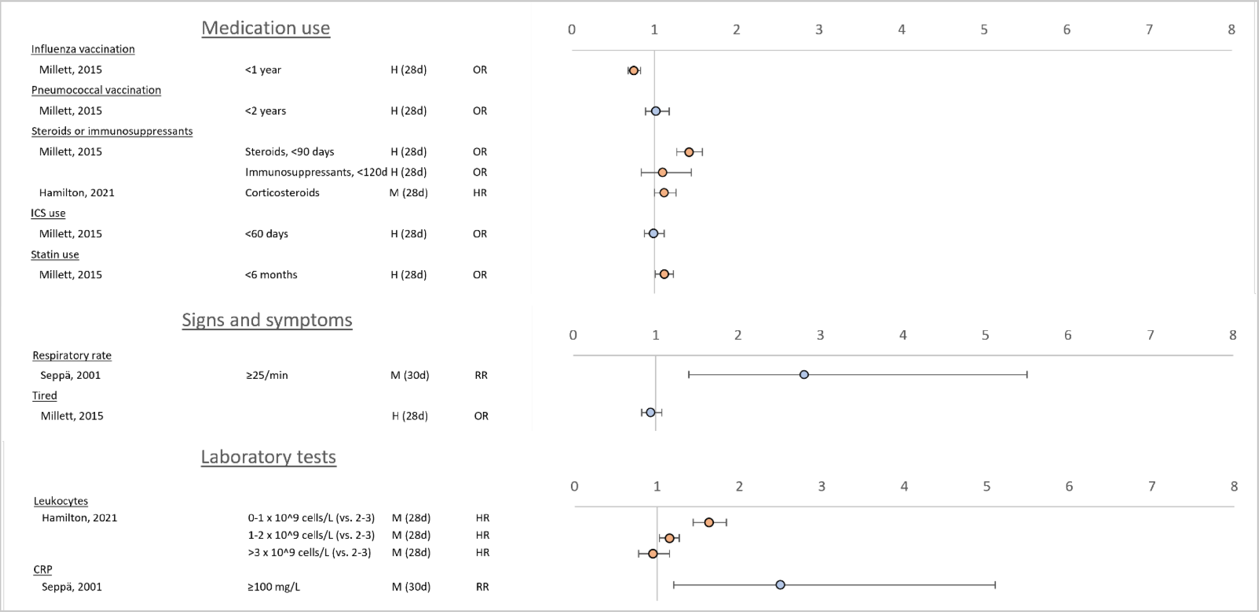

A. Multivariable analyses of prognostic factors from studies on LRTI patients. B. Multivariable analysis of prognostic factors from studies on pneumonia patients.
* Composite outcome also includes dysregulation of diabetes, stroke, heart failure, MI.
** Composite outcome also includes late onset pneumonia.
Abbreviations: LRTI, lower respiratory tract infection; H, hospitalisation; M, mortality; OR, odds ratio; RR, risk ratio; HR, hazard ratio; COPD, chronic obstructive pulmonary disease; BMI, body mass index; MI, myocardial infarction; GP, general practitioner; ICS, inhalation corticosteroids; ACE, angiotensin converting enzyme; SBP, systolic blood pressure; DBP, diastolic blood pressure; min, minute; L, liter; mg, milligram.

Table S7: Rating of the quality of evidence on promising prognostic factors based on the GRADE framework

Prognostic factor	GRADE criteria							Quality of evidence
	Study design	Risk of bias	Inconsistency	Indirectness	Imprecision	Publication bias	Upgrading factors	
Age	Cohort (high quality)	-2	0	0	0	0	0	Low
Sex	Cohort (high quality)	-2	0	-1	-1	0	0	Very low
Smoking status	Cohort (high quality)	-2	-1	-1	-1	0	0	Very low
Diabetes	Cohort (high quality)	-2	0	-1	0	0	0	Very low
Stroke	Cohort (high quality)	-2	-1	-1	-1	0	0	Very low
Cancer	Cohort (high quality)	-2	0	-1	0	0	0	Very low
Heart failure	Cohort (high quality)	-2	0	-1	0	0	0	Very low
Previous hospitalisation	Cohort (high quality)	-2	0	0	0	0	0	Low
Systemic corticosteroids	Cohort (high quality)	-2	0	-1	-1	0	0	Very low
Influenza vaccination	Cohort (high quality)	-2	0	0	-1	0	0	Very low
Recent antibiotic use	Cohort (high quality)	-2	0	0	-1	0	0	Very low
Respiratory rate	Cohort (high quality)	-2	0	-1	0	0	0	Very low
Diagnosis of pneumonia	Cohort (high quality)	-2	0	0	0	0	0	Low

Certainty of evidence			
High	Moderate	Low	Very low
4	3	2	1

Application of the GRADE framework to rate the quality of evidence was based on prognostic research-specific guidance (Foroutan et al., 2020).

Abbreviations: GRADE, grading of recommendations, assessment, development, and evaluations.

Table S8: Overview of prediction models included in the synthesis

Prediction model	Predictors included in model	Predictor weight
Bont, 2007 (new)	- Diagnosis (either acute bronchitis, COPD exacerbation, or pneumonia)<br>- Age ≥80 years<br>- Congestive heart failure<br>- Diabetes<br>- Oral glucocorticoid use<br>- Hospitalisations in previous year (0, 1, or ≥2)<br>- Antibiotic use in previous month	0, 2, or 4<br>2<br>1<br>2<br>3<br>0, 2, or 3<br>2
CRB(-65)	- Confusion<br>- Respiratory rate ≥30/minute<br>- Blood pressure (SBP ≤90mmHg or DBP ≤60mmHg)<br>(- Age ≥65 years)	1<br>1<br>1<br>1
CURB(-65)	- Predictors of CRB(-65)<br>- Blood urea nitrogen >7 mmol/L	0-4<br>1
PSI (stage 1)	- Age >50 years<br>- Altered mental status<br>- Pulse >125/minute<br>- Respiratory rate >30/minute<br>- SBP <90mmHg<br>- Temperature <35°C or ≥40°C<br>- Neoplastic disease<br>- Congestive heart failure<br>- Cerebrovascular disease<br>- Renal disease<br>- Liver disease	Any versus none
CORB-75 (new)	- Confusion<br>- Peripheral oxygen saturation ≤90%<br>- Respiratory rate ≥30/minute<br>- Blood pressure (SBP ≤90mmHg or DBP ≤60mmHg)<br>- Age ≥75 years	1<br>1<br>1<br>1<br>1
RISSC85 (new)	- Risk of poor outcome, grouped by country (A: Spain, B: Belgium, the Netherlands, Poland, UK, C: Germany)<br>- Interference in daily activities (some versus severe)<br>- Number of years stopped smoking (>45 years)<br>- Severe sputum<br>- Presence of crackles<br>- Diastolic blood pressure (<85mmHg)	A = 0, B/C = 1<br><br>1<br>1<br>1<br>1<br>1
Moore, 2019 (new)	- Oxygen saturation <95%<br>- Age >65 years<br>- Blood pressure (SBP <90mmHg or DBP <60mmHg)<br>- Temperature >37.8°C<br>- Any comorbidity (cardiovascular, cerebrovascular or lung comorbidities)<br>- No coryza<br>- Severity assessment >5/10 by GP<br>- Chest pain	1<br>1<br>1<br>1<br>1<br><br>1<br>1<br>1

Abbreviations: COPD, chronic obstructive pulmonary disease; SBP, systolic blood pressure; DBP, diastolic blood pressure; GP, general practitioner.

Table S9: Rating of the quality of evidence on prediction models based on the GRADE framework

Prediction model	GRADE criteria							Quality of evidence
	Study design	Risk of bias	Inconsistency	Indirectness	Imprecision	Publication bias	Upgrading factors	
Bont, 2007	Cohort (high quality)	-2	0	0	-1	0	0	Very low
CRB	Cohort (high quality)	-2	0	-1	-1	0	0	Very low
CRB-65	Cohort (high quality)	-2	0	0	0	0	0	Low
CURB	Cohort (high quality)	-2	0	-1	-1	0	0	Very low
CURB-65	Cohort (high quality)	-2	0	-1	-1	0	0	Very low
PSI	Cohort (high quality)	-2	0	-1	-1	0	0	Very low
CORB-75	Cohort (high quality)	-2	0	-1	-1	0	0	Very low
RISCC85	Cohort (high quality)	-2	0	-1	-1	0	0	Very low
Moore, 2019	Cohort (high quality)	-2	0	-1	-1	0	0	Very low

Certainty of evidence			
High	Moderate	Low	Very low
4	3	2	1

Application of the GRADE framework to rate the quality of evidence was based on prognostic research-specific guidance (Brozek et al., 2021).

Abbreviations: GRADE, grading of recommendations, assessment, development, and evaluations.
