# Supplementary material for: Prognostic factors and prediction models for hospitalisation and all-cause mortality in adults presenting to primary care with a lower respiratory tract infection: a systematic review
Source: BMJ Open. 2024 Mar 23;14(3):e075475. doi: 10.1136/bmjopen-2023-075475 (PMC10961536; doi:10.1136/bmjopen-2023-075475)
Supplement: Author's manuscript [file bmjopen-2023-075475.draft_revisions.pdf]

BMJ Open is committed to open peer review. As part of this commitment we make the peer review history of every article we publish publicly available.

When an article is published we post the peer reviewers' comments and the authors' responses online. We also post the versions of the paper that were used during peer review. These are the versions that the peer review comments apply to.

The versions of the paper that follow are the versions that were submitted during the peer review process. They are not the versions of record or the final published versions. They should not be cited or distributed as the published version of this manuscript.

BMJ Open is an open access journal and the full, final, typeset and author-corrected version of record of the manuscript is available on our site with no access controls, subscription charges or pay-per-view fees (http://bmjopen.bmj.com).

If you have any questions on BMJ Open's open peer review process please email info.bmjopen@bmj.com

BMJ Open

Prognostic factors and prediction models for hospitalisation and all-cause mortality in adults presenting to primary care with a lower respiratory tract infection: a systematic review

Journal:	*BMJ Open*
Manuscript ID	bmjopen-2023-075475
Article Type:	Original research
Date Submitted by the Author:	10-May-2023
Complete List of Authors:	Rijk, Merijn; University Medical Centre Utrecht, Julius Center for Health Sciences and Primary Care<br>Platteel, Tamara ; University Medical Centre Utrecht, Julius Center for Health Sciences and Primary Care<br>van den Berg, Teun; University Medical Centre Utrecht, Julius Center for Health Sciences and Primary Care<br>Geersing, Geert-Jan; Julius Center for Health Sciences and Primary Care, Department of General Practice<br>Little, Paul; University of Southampton, Primary Care and Population Science;<br>Rutten, Frans; Julius Centre for Health Sciences and Primary Care, University Medical Centre Utrecht, Department of General Practice<br>van Smeden, Maarten; University Medical Center Utrecht, Julius Center for Health Sciences and Primary Care<br>Venekamp , Roderick ; University Medical Center, Utrecht, Julius Center for Health Sciences and Primary Care
Keywords:	Respiratory infections < THORACIC MEDICINE, Primary Care < Primary Health Care, Prognosis, Systematic Review
Note: The following files were submitted by the author for peer review, but cannot be converted to PDF. You must view these files (e.g. movies) online.	
Supplementary data.docx	

SCHOLARONE™
Manuscripts

I, the Submitting Author has the right to grant and does grant on behalf of all authors of the Work (as defined in the below author licence), an exclusive licence and/or a non-exclusive licence for contributions from authors who are: i) UK Crown employees; ii) where BMJ has agreed a CC-BY licence shall apply, and/or iii) in accordance with the terms applicable for US Federal Government officers or employees acting as part of their official duties; on a worldwide, perpetual, irrevocable, royalty-free basis to BMJ Publishing Group Ltd ("BMJ") its licensees and where the relevant Journal is co-owned by BMJ to the co-owners of the Journal, to publish the Work in this journal and any other BMJ products and to exploit all rights, as set out in our licence.

The Submitting Author accepts and understands that any supply made under these terms is made by BMJ to the Submitting Author unless you are acting as an employee on behalf of your employer or a postgraduate student of an affiliated institution which is paying any applicable article publishing charge ("APC") for Open Access articles. Where the Submitting Author wishes to make the Work available on an Open Access basis (and intends to pay the relevant APC), the terms of reuse of such Open Access shall be governed by a Creative Commons licence – details of these licences and which Creative Commons licence will apply to this Work are set out in our licence referred to above.

Other than as permitted in any relevant BMJ Author's Self Archiving Policies, I confirm this Work has not been accepted for publication elsewhere, is not being considered for publication elsewhere and does not duplicate material already published. I confirm all authors consent to publication of this Work and authorise the granting of this licence.

For peer review only

Title page

Title

Prognostic factors and prediction models for hospitalisation and all-cause mortality in adults presenting to primary care with a lower respiratory tract infection: a systematic review

Authors

Merijn H. Rijk[1], MD; Tamara N. Platteel[1], MD PhD; Teun M. C. van den Berg[1], MD; Geert-Jan Geersing[1], MD PhD; Paul Little[2], MD PhD; Frans H. Rutten[1], MD PhD; Maarten van Smeden[3], PhD; Roderick P. Venekamp[1], MD PhD

Affiliations

[1] Department of General Practice & Nursing Science, Julius Center for Health Sciences and Primary Care, University Medical Center Utrecht, Utrecht University, Utrecht, the Netherlands

[2] Primary Care Research Centre, Primary Care Population Sciences and Medical Education Unit, University of Southampton, Southampton, United Kingdom

[3] Department of Epidemiology & Health Economics, Julius Center for Health Sciences and Primary Care, University Medical Center Utrecht, Utrecht University, Utrecht, the Netherlands

MvS and RPV contributed equally to this work.

Corresponding author

Merijn H. Rijk, MD

For peer review only - http://bmjopen.bmj.com/site/about/guidelines.xhtml

Department of General Practice & Nursing Science, Julius Center for Health Sciences and Primary Care, University Medical Center Utrecht, Utrecht University, Utrecht, the Netherlands

Universiteitsweg 100, 3584 CG Utrecht

Phone: 0031 88 75 681 81

Email: m.h.rijk@umcutrecht.nl

For peer review only

Prior presentation(s)

None

Word count

Abstract: 298

Main text: 2930

Number of tables, figures, and appendices

Tables: 2

Figures: 3

Appendices: 6

For peer review only - http://bmjopen.bmj.com/site/about/guidelines.xhtml

Abstract

Objective To identify and synthesize relevant existing prognostic factors (PF) and prediction models (PM) for hospitalisation and all-cause mortality within 90 days in primary care patients with acute lower respiratory tract infections (LRTI).

Design Systematic review.

Methods Systematic searches of MEDLINE, Embase, and the Cochrane Library were performed in June 2022. All PF and PM studies on risk of hospitalisation or all-cause mortality within 90 days in adult primary care LRTI patients were included. Risk of bias was assessed using the QUIPS and PROBAST tools for PF and PM studies, respectively. Results of included PF and PM studies were descriptively summarised.

Results Of 2,799 unique records identified, 16 were included: nine PF studies, six PM studies, and one combination of both. Risk of bias was judged high for all studies, mainly due to limitations in the analysis domain. Based on reported multivariable associations in PF studies, age, sex, current smoking, diabetes, a history of stroke, cancer, or heart failure, previous hospitalisation, influenza vaccination (negative association), corticosteroid use, recent antibiotic use, respiratory rate ≥25/minute, and diagnosis of pneumonia were identified as most promising candidate predictors. One newly developed PM was externally validated (c statistic 0.74, 95% confidence interval (CI) 0.71 to 0.78) whereas the hospital-derived CRB-65 was externally validated in primary care in five studies (c statistic ranging from 0.72 [95% CI 0.63 to 0.81] to 0.79 [95% CI 0.65 to 0.92]). None of the PM studies reported measures of model calibration.

Conclusions Implementation of existing models for individualised risk prediction of 90-day hospitalisation or mortality in primary care LRTI patients in everyday practice is hampered by incomplete assessment of model performance. The identified candidate predictors provide useful information for clinicians and warrant consideration when developing or updating PMs using state-of-the-art development and validation techniques.

PROSPERO registration number CRD42022341233

For peer review only - http://bmjopen.bmj.com/site/about/guidelines.xhtml

Strengths and limitations of this study

- This systematic review provides a comprehensive overview of available evidence on the prognosis of adults presenting to primary care with lower respiratory tract infections, by covering both prognostic factors and prediction models for hospitalisation and all-cause mortality.
- A broad systematic search was conducted, with incorporation of a validated prognostic filter to ensure all relevant prognostic literature has been identified.
- Due to substantial heterogeneity in domain, predictor, and outcomes definitions, meta-analysis of prognostic factor effect estimates and prediction model performance was not performed.

For peer review only

For peer review only - http://bmjopen.bmj.com/site/about/guidelines.xhtml

Keywords

Lower respiratory tract infections; primary care; general practice; prognosis; prognostic factor; prediction model; systematic review

Abbreviations

LRTI	Lower respiratory tract infections
CAP	Community-acquired pneumonia
GP	General practitioner
PSI	Pneumonia severity index
PF	Prognostic factor
PM	Prediction model
CHARMS	Checklist for Critical Appraisal and Data Extraction for Systematic Reviews of Prediction Modelling Studies
QUIPS	Quality in Prognostic Studies
PROBAST	Prediction Model Risk Of Bias Assessment Tool
CI	Confidence interval
O/E	Observed over expected
COVID-19	Coronavirus disease 2019
NICE	National Institute for Health and Care Excellence
ATS	American Thoracic Society
IDSA	Infectious Diseases Society of America

For peer review only

Introduction

Acute lower respiratory tract infections (LRTI) are common in primary care.(1,2) Uncomplicated LRTI episodes generally have a favourable natural course in otherwise healthy adults, and antibiotics confer only little benefit in terms of earlier symptom resolution, both overall and in higher-risk subgroups of patients.(3,4) Additionally, observational evidence suggests that antibiotics do not substantially reduce subsequent hospital admission or death for adults with uncomplicated LRTI, and such adverse outcomes occur in fewer than 1% of patients.(5) Within the clinical spectrum of LRTI, the risk of hospitalisation and death is far more pronounced among adults with community-acquired pneumonia (CAP).(6,7) However, in patients presenting with LRTI symptoms to their general practitioner (GP), differentiating CAP (or presence of bacterial pathogens) from virally induced LRTI symptoms is notoriously challenging(8,9), leaving clinicians with uncertainties how to best manage these patients.

Identification of LRTI patients at highest risk of complications, such as hospitalisation and death, could help GPs identify those in whom close follow-up or early (antibiotic) treatment is warranted. Currently, several scores exist to assist physicians in estimating risk of adverse outcomes in LRTI patients, such as the Pneumonia Severity Index (PSI) and CURB-65 (a scoring rule including confusion, blood urea nitrogen, respiratory rate, blood pressure, and age ≥65 years).(10,11) However, these prediction rules have been developed in secondary care and the inclusion of laboratory and/or radiographic features hampers applicability to outpatients. In addition, an overview of primary care-specific prognostic factors (PF) and prediction models (PM) for adverse outcomes in LRTI patients is currently lacking. We therefore aimed to identify and synthesize existing PFs and PMs for hospitalisation and mortality within 90 days in adult patients presenting to primary care with LRTI.

For peer review only

Methods

The protocol for this review was registered in PROSPERO (CRD42022341233) and the review was reported according to the PRISMA reporting guideline for systematic reviews.(12)

Searches and study selection

The electronic databases of MEDLINE, Embase, and the Cochrane Library were searched on 27 June 2022 for eligible studies. Keywords for 'respiratory tract infection', 'hospitalisation', 'death', 'adults' and 'primary care' were combined with a filter to identify relevant prognostic studies (see S1 for full search string).(13) Eligibility criteria were based on PICOTS guidance; we included all studies on adults with LRTI (patients) in which prognostic factors or prediction models (index) for 90-day hospitalisation or all-cause mortality (outcome) were assessed on the day of diagnosis (timing) in primary care (setting).(14,15) Non-English articles or those without full-text availability were excluded.

After de-duplication, two review authors (MHR, TMCB) independently reviewed titles/abstracts of unique records retrieved from the electronic databases against the eligibility criteria. Next, the same authors independently reviewed the full text of potentially relevant articles for eligibility. Any disagreements were resolved by discussion and, where necessary, by consulting a third reviewer (TNP). To increase the yield of potentially eligible articles, reference lists of the included articles were reviewed for eligibility.

Data collection

Data from eligible PF and PM studies were extracted by MHR using standardised forms based on the Checklist for Critical Appraisal and Data Extraction for Systematic Reviews of PF studies (CHARMS-PF) and PM studies (CHARMS), respectively.(14,16) Main outcomes of interest were hospitalisation or death within 90 days after initial LRTI consultation. For PF studies we extracted odds ratios, hazard ratios, or risk ratios with accompanying 95% confidence intervals (CI) from both univariable and multivariable analyses. For those studies that did not report

For peer review only - http://bmjopen.bmj.com/site/about/guidelines.xhtml

such estimates, these associations were calculated from crude data using Rothman's Episheet version 15 November 2021 wherever possible. For PM studies, model performance estimates in terms of discrimination (c statistic with accompanying 95% CI) and calibration (intercept and slope) were extracted. If calibration measures were absent, the total observed over expected (O/E) ratio with accompanying 95% CI of patients with the outcome was calculated wherever possible as a proxy of overall model calibration.(15) To this end, we used data on the distribution of subjects and outcomes across different PM risk strata and the corresponding predicted risks of the strata as reported in the original model development study. These calculations were performed in R version 4.2.2 using the 'metamisc' package.

Other data extraction items included study population, candidate predictors, sample size, (handling of) missing data, analysis (modelling method and/or model development and validation method), and interpretation of results. Where needed, corresponding authors of individual studies were contacted to request additional data.

Assessment of risk of bias and applicability

Risk of bias assessment was performed by two authors (MHR, TNP) independently using the Quality in Prognostic Studies tool (QUIPS, assessing study participation, study attrition, PF measurement, outcome measurement, and statistical analysis) and the Prediction Model Risk Of Bias Assessment Tool (PROBAST, covering the domains of participants, predictors, outcome, and analysis) for PF and PM studies, respectively.(17,18) We excluded the 'study confounding' item from the QUIPS, since confounding is not applicable to prognostic research. Applicability of included articles was assessed based on the PICOTS criteria.

Synthesis methods

Due to substantial heterogeneity in domain, predictor, and outcome definition, combining PF effect estimates or PM performance measures in meta-analyses was considered inappropriate and results were therefore descriptively summarised, stratified according to diagnosis (i.e.

For peer review only - http://bmjopen.bmj.com/site/about/guidelines.xhtml

LRTI versus pneumonia). Forest plots were created to visually summarise (i) univariable and multivariable effect estimates of PFs, and (ii) discrimination and calibration performance measures of PMs. PFs were categorised into either demographics, patient history, healthcare use, medication use, signs and symptoms, laboratory tests, or diagnosis. Promising candidate predictors were identified based on reported results of multivariable analysis in PF studies.

For peer review only

For peer review only - http://bmjopen.bmj.com/site/about/guidelines.xhtml

Results

Study selection

The search strategy yielded a total of 3,191 records. Removing duplicates left 2,799 unique records (Figure 1). After screening title and abstract, 34 studies remained for full-text review. Of these, 19 were excluded (Figure 1). One additional publication was retrieved from screening reference lists(19), leaving 16 publications for inclusion: nine PF studies(19–27), six PM studies(28–33), and one combination of PM and PF(34).

Study characteristics

The PF studies were conducted between 1997-2021 of which most (7/10) used a retrospective cohort design (Table 1). The study population in terms of age (minimum age ranging from 1-80 years) and LRTI definition (ranging from suspected LRTI to diagnosis of CAP), as well as the hospitalisation rate (0.7 to 76.5%) varied substantially across studies. Mortality was used as outcome in four studies, hospitalisation in three studies, and a composite of both in one study. Two studies also included deterioration of existing illness and late-onset pneumonia in addition to hospitalisation and mortality in their outcome(21,34), while the timing of outcome assessment was unknown in one study(27).

The PM studies were conducted between 2000-2013 and the majority (4/7) used a prospective cohort design. In total, nine different PMs could be included in the synthesis (Table 2). Four studies developed a new prediction model(28,30,33,34) and five externally validated existing models(29–33), of which the CRB-65 was validated in all five studies. Clinical heterogeneity was mostly attributed to age (minimum age ranging from 16-65 years) and LRTI definition (ranging from suspected LRTI to radiographically confirmed CAP). Hospitalisation rates varied between 0.5 and 86.9%. All PM studies used binary outcomes. Mortality was used as outcome in three studies, hospitalisation in one study, and a composite of both in one study. One study also included re-consultation with new or worsened symptoms in addition to

For peer review only - http://bmjopen.bmj.com/site/about/guidelines.xhtml

hospitalisation in their outcome(33), while another study included late-onset pneumonia in addition to hospitalisation and mortality.(34)

Risk of bias assessment

Overall risk of bias was judged high for all studies (Figures S2 and S3). For PF studies, this was mostly due to issues in the statistical analysis domains. For instance, five studies selected predictors for multivariable analysis based on univariable associations(19–23), and two included multiple episodes per patient but did not perform recurrent event analysis.(23,25) Furthermore, in some studies an adequate definition of LRTI or PFs was lacking, and methods to account for missing data were mostly judged inadequate or not described.

Similarly, risk of bias due to the issues in the analysis domain was judged high for all PM studies. In all model development studies continuous predictors were dichotomised, and handling of missing data was judged inadequate or not reported. None of the PM studies reported any evaluation of model calibration.

Prognostic factors

Three PF studies did not report estimates of univariable associations of PFs, and these could also not be calculated from the reported data.(22,25,26) The remaining PF studies reported univariable analysis of 69 PFs, of which sex, age, and history of pulmonary disease were analysed most frequently (Figure S4).

Estimates of multivariable associations were reported for 74 PFs (Figure S5), of which 21 were analysed in at least two studies. The number of covariates per multivariable analysis ranged from 3 to 29, and a common set of covariates across studies was lacking. Age was analysed in five studies, and all found increasing age to be associated with poor outcome.(21,22,25,26,34) Apart from age, PFs that were identified as promising based on

For peer review only - http://bmjopen.bmj.com/site/about/guidelines.xhtml

For peer review only

multiple multivariable analyses were sex (five studies), current smoking (four studies), diabetes (three studies), a history of stroke (three studies), cancer (three studies), or heart failure (two studies), hospitalisation in the previous year (two studies), current corticosteroid use (three studies), antibiotic use in the previous month (three studies), a respiratory rate ≥25/minute (two studies), and diagnosis of pneumonia (two studies) (Figure 2). Having received an influenza vaccination in the previous year was the only promising candidate predictor estimated to have a negative association, based on multivariable analyses from three studies.

Prediction models

Several PMs included predictors that were also identified as promising candidate predictors based on PF studies (Table S6). Sex, current smoking, and influenza vaccination were the only promising candidate predictors that were not incorporated in any of the PMs. Predictors that were not identified as promising PFs but were incorporated in PMs include history of lung, renal or liver disease, extent to which complaints interfere with daily activities, sputum or coryza, chest pain, confusion, presence of crackles, heart rate, blood pressure, oxygen saturation, body temperature, blood urea nitrogen, and an overall GP severity assessment score.

Four studies developed a new PM (three for use in LRTI and one for CAP patients), in which the number of events per predictor ranged from 3.3-13.7.(28,32–34) C statistics ranged from 0.63 (95% CI 0.61-0.67) to 0.82 (95% CI 0.75-0.88) after (internal or external) model validation (Figure 3). One study performed external validation(28), whereas other studies only conducted internal(-external) validation.(32–34) None reported measures of model calibration, while a total O/E ratio could not be calculated for any model.

All five PM validation studies externally validated the CRB-65.(29–33) C statistics for 30-day mortality in CAP patients (range outcome events: 11 to 80) ranged from 0.72 (95% CI 0.66-0.78) to 0.79 (95% CI 0.65-0.92)(29,30,32) (Figure 3). The c statistic for 28-day re-

For peer review only - http://bmjopen.bmj.com/site/about/guidelines.xhtml

consultation or hospitalisation in LRTI patients was substantially lower: 0.53 (95% CI 0.51-0.56).(33) None reported measures of model calibration. A total O/E ratio could be calculated for the CRB-65 in four studies, ranging from 0.67 (95% CI 0.26-1.08) to 1.42 (95% CI 1.13-1.71).

Other validated PMs were PSI, CURB-65, CURB, and CRB. C statistics for 28-day re-consultation or hospitalisation in LRTI patients ranged from 0.51 (95% CI 0.50-0.54) to 0.53 (95% CI 0.50-0.55).(33) Calibration measures were not reported and total O/E ratios could not be calculated. C statistics of PSI and CURB-65 for 30-day mortality in CAP patients were 0.73 (95% CI 0.67-0.79) and 0.67 (95% CI 0.61-0.74), respectively.(30) Model calibration measures were not reported, while the total O/E ratios were estimated at 1.46 (95% CI 1.16-1.76) and 1.92 (95% CI 1.53-2.32), respectively.

For peer review only

Discussion

Summary of main findings

This systematic review summarised evidence on PFs and PMs for hospitalisation and mortality within 90 days in patients presenting to primary care with LRTI. The risk of bias of included studies was judged high, mainly due to issues in the analysis domains. Of all PFs analysed, thirteen were identified as most promising; age, sex, current smoking, diabetes, a history of stroke, cancer, or heart failure, hospitalisation in the previous year, current corticosteroid use, antibiotic use in the previous month, a respiratory rate ≥25/minute, and diagnosis of pneumonia were associated with poor outcome whereas seasonal influenza vaccination was estimated to have a negative association. Most of these promising candidate predictors have also been incorporated in the identified PMs. The secondary care-derived CRB-65, predicting mortality in CAP patients, and a newly developed PM by Bont et al.(28) predicting hospitalisation and mortality in elderly LRTI patients both showed promising results after external validation. However, none assessed model calibration, leaving the precision of predicted risks unknown.

Comparison with existing literature

During the coronavirus disease 2019 (COVID-19) pandemic, many studies have focused on PFs associated with poor outcomes in COVID-19 patients, and some were similar to those identified in our review of all-cause LRTI patients. A recent, field-wide systematic review and meta-analysis of PFs for adverse outcomes in COVID-19 patients found cancer, insulin use, and smoking – among other factors – to be associated with mortality, and age with both hospitalisation and mortality.(35) Another community-based systematic review of European COVID-19 patients found male sex, heart failure, and diabetes to be associated with hospitalisation, whereas stroke, heart failure, and neurologic disease were associated with increased mortality risk.(36)

For peer review only - http://bmjopen.bmj.com/site/about/guidelines.xhtml

Some of the identified PMs have been incorporated in LRTI guidelines – mainly those focusing on CAP – as additional tool to aid patient management decisions. The CAP guideline issued by the American Thoracic Society (ATS) and the Infectious Diseases Society of America (IDSA) supports the use of PSI or CURB-65 – in addition to clinical judgement – to determine the appropriate level of care, i.e. inpatient versus outpatient management.(37,38) Although this guideline applies to all levels of health care, the value of the suggested models for GPs is limited since models' laboratory and radiographic features are not routinely available in primary care. The UK National Institute for Health and Care Excellence (NICE) guideline on pneumonia advocates the use of CRB-65; it recommends GPs to consider hospital admission in patients with a score of ≥1.(39) Guidelines vary with regard to the incorporation of individual factors that are associated with poor outcome. The ATS/IDSA guideline and the Dutch primary care guideline both list tachypnoea and confusion as indicators of disease severity, while the Dutch guideline also emphasised the importance of PFs such as age, history of heart failure, and diabetes.(37,40) The NICE guideline does not explicitly highlight individual factors associated with poor outcome in adult patients.(39)

Similar to our findings, a review of the performance of CRB-65 for predicting 30-day mortality among CAP patients in community and hospital settings concluded that this model has been insufficiently validated for use in primary care.(41) Although the population of this review slightly differed from ours by also including patients who visited the emergency department, a similarly low number of outcome events was found. Contrary to the slight overprediction reported in this review, our synthesis of more recent studies suggested underprediction of risks based on total O/E ratios. However, in absence of proper assessment of model calibration the true precision of predicted risks remains to be elucidated.

Strengths and limitations

For peer review only - http://bmjopen.bmj.com/site/about/guidelines.xhtml

By covering both PFs and PMs in a single review, we have provided a comprehensive overview of available prognostic literature of primary care LRTI patients. We prospectively registered our review protocol, conducted a broad search with incorporation of a validated prognostic filter, and thoroughly reviewed citations of eligible studies to assure all relevant literature was included in the synthesis.

Some limitations should, however, be acknowledged. First, our findings should be interpreted with caution since risk of bias was judged high for all included studies. Second, due to substantial heterogeneity in domain, predictor, and outcome definitions between studies, we refrained from conducting meta-analyses of PF effect estimates or PM performance measures, which makes direct comparison of study results difficult. It is plausible that PF effect estimates for poor outcome vary according to age, diagnosis, and disease severity (a priori risk of event); for example, half of the participants in Hak et al.(21) had either pneumonia or an exacerbation of chronic obstructive pulmonary disease, whereas Moore et al.(34) had fewer than 2% of such patients. Consistency in effect estimates would have further facilitated direct comparison of PFs, but converting the various reported estimates to odds ratios appeared not possible. Albeit our synthesis of PFs provides some useful information for clinicians, it merely informs future research aimed at developing or updating PMs. Third, we excluded most studies on specific respiratory pathogens such as SARS-CoV-2 and influenza since these included patients with both upper and lower respiratory tract infections and did not report stratified results. As such, our results are not applicable to these specific patient populations. Lastly, PM building techniques were generally suboptimal which increased risk of bias, and none of the identified PM studies assessed model calibration. In contrast to discrimination, calibration generally receives little attention when assessing PM performance, whereas well discriminating but poorly calibrated models can lead to imprecise, misleading predictions.(42) Total O/E ratios calculations provided some insight into mean model calibration – the most basic level in the hierarchy of calibration assessment – but this is insufficient to provide guidance on the safe use of a PM in clinical practice.(43)

For peer review only

Implications for research and clinical practice

Based on our synthesis, implementation of existing PMs for individualised risk prediction of 90-day hospitalisation or all-cause mortality in primary care LRTI patients in everyday practice is hampered by incomplete assessment of model performance. The identified candidate predictors provide useful information for clinicians about factors associated with poor outcome in primary care LRTI patients and warrant consideration when developing or updating PMs using state-of-the-art development and appropriate validation techniques.

For peer review only - http://bmjopen.bmj.com/site/about/guidelines.xhtml

Declarations

Author contributions

MHR, TNP, MvS, and RPV were involved in the conceptualisation and design of the study. MHR, TMCB, and TNP conducted the study selection procedure. Data of eligible studies was extracted by MHR. MHR, TNP, and MvS performed the risk of bias assessment. MHR wrote the first draft of the manuscript, which was initially reviewed by TNP, MvS, and RVP. All authors read and approved the final manuscript.

Funding

This study was funded by ZonMw (grant number 08391052110003). The funder played no role in study design, data collection, analysis and interpretation of data, or the writing of this manuscript.

Competing interests

All authors declare no financial or non-financial competing interests.

Data availability

Study materials (e.g. template data collection forms, data extracted from included studies) are available from the corresponding author upon reasonable request.

For peer review only

For peer review only - http://bmjopen.bmj.com/site/about/guidelines.xhtml

References

1. Bergmann M, Haasenritter J, Beidatsch D, Schwarm S, Hörner K, Bösner S, et al. Prevalence, aetiologies and prognosis of the symptom cough in primary care: a systematic review and meta-analysis. BMC Fam Pract. 2021;22(1):1–19.

2. Hak E, Rovers MM, Kuyvenhoven MM, Schellevis FG, Verheij TJM. Incidence of GP-diagnosed respiratory tract infections according to age, gender and high-risk co-morbidity: The Second Dutch National Survey of General Practice. Fam Pract. 2006;23(3):291–4.

3. Little P, Stuart B, Moore M, Coenen S, Butler CC, Godycki-Cwirko M, et al. Amoxicillin for acute lower-respiratory-tract infection in primary care when pneumonia is not suspected: A 12-country, randomised, placebo-controlled trial. Lancet Infect Dis. 2013;13(2):123–9.

4. Moore M, Stuart B, Coenen S, Butler CC, Goossens H, Verheij TJM, et al. Amoxicillin for acute lower respiratory tract infection in primary care: Subgroup analysis of potential high-risk groups. British Journal of General Practice. 2014;64(619):75–80.

5. Little P, Stuart B, Smith S, Thompson MJ, Knox K, Van Den Bruel A, et al. Antibiotic prescription strategies and adverse outcome for uncomplicated lower respiratory tract infections: Prospective cough complication cohort (3C) study. BMJ (Online). 2017;357.

6. Welte T, Torres A, Nathwani D. Clinical and economic burden of community-acquired pneumonia among adults in Europe. Thorax. 2012;67(1):71–9.

7. McLaughlin JM, Khan FL, Thoburn EA, Isturiz RE, Swerdlow DL. Rates of hospitalization for community-acquired pneumonia among US adults: A systematic review. Vaccine. 2020;38(4):741–51.

8. Teepe J, Broekhuizen BDL, Loens K, Lammens C, Ieven M, Goossens H, et al. Predicting the presence of bacterial pathogens in the airways of primary care patients with acute cough. CMAJ. 2017;189(2):E50–5.

9. Minnaard MC, De Groot JAH, Hopstaken RM, Schierenberg A, De Wit NJ, Reitsma JB, et al. The added value of C-reactive protein measurement in diagnosing pneumonia in primary care: A meta-analysis of individual patient data. CMAJ. 2017;189(2):E56–63.

10. Lim WS, Van Der Eerden MM, Laing R, Boersma WG, Karalus N, Town GI, et al. Defining community acquired pneumonia severity on presentation to hospital: An international derivation and validation study. Thorax. 2003;58(5):377–82.

11. Fine M, Auble T, Yealy D, Hanusa B, Weissfeld L, Singer D, et al. A prediction rule to identify low-risk patients with community-acquired pneumonia. New England Journal of Medicine. 1997;336(4):243–50.

12. Page MJ, McKenzie JE, Bossuyt PM, Boutron I, Hoffmann TC, Mulrow CD, et al. The PRISMA 2020 statement: An updated guideline for reporting systematic reviews. The BMJ. 2021;372.

13. Geersing GJ, Bouwmeester W, Zuithoff P, Spijker R, Leeflang M, Moons K. Search filters for finding prognostic and diagnostic prediction studies in medline to enhance systematic reviews. PLoS One. 2012;7(2):3–8.

For peer review only

14. Moons KGM, de Groot JAH, Bouwmeester W, Vergouwe Y, Mallett S, Altman DG, et al. Critical Appraisal and Data Extraction for Systematic Reviews of Prediction Modelling Studies: The CHARMS Checklist. PLoS Med. 2014;11(10).

15. Debray TPA, Damen JAAG, Snell KIE, Ensor J, Hooft L, Reitsma JB, et al. A guide to systematic review and meta-analysis of prediction model performance. BMJ (Online). 2017;356.

16. Riley RD, Moons KGM, Snell KIE, Ensor J, Hooft L, Altman DG, et al. A guide to systematic review and meta-analysis of prognostic factor studies. BMJ (Online). 2019;364.

17. Hayden JA, van der Windt DA, Cartwright JL, Côté P, Bombardier C. Assessing Bias in Studies of Prognostic Factors. Ann Intern Med. 2013;158:280–6.

18. Wolff RF, Moons KGM, Riley RD, Whiting PF, Westwood M, Collins GS, et al. PROBAST: A tool to assess the risk of bias and applicability of prediction model studies. Ann Intern Med. 2019;170(1):51–8.

19. Houston MS, Silverstein MD, Suman VJ. Risk Factors for 30-Day Mortality in Elderly Patients With Lower Respiratory Tract Infection: Community-Based Study. Arch Intern Med. 1997;157:2190–5.

20. Seppä Y, Bloigu A, Honkanen PO, Miettinen L, Syrjälä H. Severity assessment of lower respiratory tract infection in elderly patients in primary care. Arch Intern Med. 2001;161(22):2709–13.

21. Hak E, Bont J, Hoes AW, Verheij TJM. Prognostic factors for serious morbidity and mortality from community-acquired lower respiratory tract infections among the elderly in primary care. Fam Pract. 2005;22(4):375–80.

22. Winchester CC, Macfarlane T v., Thomas M, Price D. Antibiotic prescribing and outcomes of lower respiratory tract infection in UK primary care. Chest. 2009;135(5):1163–72.

23. van de Nadort C, Smeets HM, Bont J, Zuithoff NPA, Hak E, Verheij TJM. Prognosis of primary care patients aged 80 years and older with lower respiratory tract infection. British Journal of General Practice. 2009;59(561):261–5.

24. Myles P, Hubbard R, Gibson J, Pogson Z, Smith C, McKeever T. The impact of statins, ace inhibitors and gastric acid suppressants on pneumonia mortality in a UK general practice population cohort. Pharmacoepidemiol Drug Saf. 2009;18(May):697–703.

25. Millett ERC, de Stavola BL, Quint JK, Smeeth L, Thomas SL. Risk factors for hospital admission in the 28 days following a community-acquired pneumonia diagnosis in older adults, and their contribution to increasing hospitalisation rates over time: A cohort study. BMJ Open. 2015;5(12).

26. Hamilton F, Arnold D, Payne R. Association of prior lymphopenia with mortality in pneumonia: A cohort study in UK primary care. British Journal of General Practice. 2021;71(703):E148–56.

27. Martínez-Redondo J, Comas C, Salud JP, Crespo-Pons M, García-Serrano C, Bravo MO, et al. The risk of hospitalization in COVID-19 patients can be predicted by lung ultrasound in primary care. Int J Environ Res Public Health. 2021;18(11).

For peer review only - http://bmjopen.bmj.com/site/about/guidelines.xhtml

28. Bont J, Hak E, Hoes AW, Schipper M, Schellevis FG, Verheij TJM. A prediction rule for elderly primary-care patients with lower respiratory tract infections. European Respiratory Journal. 2007;29(5):969–75.

29. Bont J, Hak E, Hoes AW, Macfarlane JT, Verheij TJ. Predicting death in elderly patients with community-acquired pneumonia: a prospective validation study reevaluating the CRB-65 severity assessment tool. Arch Intern Med. 2008;168(13):1465–8.

30. Ochoa-Gondar O, Vila-Corcoles A, Rodriguez-Blanco T, Ramos F, de Diego C, Salsench E, et al. Comparison of three predictive rules for assessing severity in elderly patients with CAP. Int J Clin Pract. 2011;65(11):1165–72.

31. Francis NA, Cals JW, Butler CC, Hood K, Verheij T, Little P, et al. Severity assessment for lower respiratory tract infections: Potential use and validity of the CRB-65 in primary care. Primary Care Respiratory Journal. 2012;21(1):65–70.

32. Ochoa-Gondar O, Vila-Corcoles A, Rodriguez-Blanco T, Hospital I, Salsench E, Ansa X, et al. Validation of the CORB75 (confusion, oxygen saturation, respiratory rate, blood pressure, and age ≥75 years) as a simpler pneumonia severity rule. Infection. 2013;42(2):371–8.

33. Bruyndonckx R, Hens N, Verheij TJM, Aerts M, Ieven M, Butler CC, et al. Development of a prediction tool for patients presenting with acute cough in primary care: A prognostic study spanning six European countries. British Journal of General Practice. 2018;68(670):e342–50.

34. Moore M, Stuart B, Lown M, van den Bruel A, Smith S, Knox K, et al. Predictors of adverse outcomes in uncomplicated lower respiratory tract infections. Ann Fam Med. 2019;17(3):231–8.

35. Bellou V, Tzoulaki I, van Smeden M, Moons KGM, Evangelou E, Belbasis L. Prognostic factors for adverse outcomes in patients with COVID-19: A field-wide systematic review and meta-analysis. Vol. 59, European Respiratory Journal. European Respiratory Society; 2022.

36. Vardavas CI, Mathioudakis AG, Nikitara K, Stamatelopoulos K, Georgiopoulos G, Phalkey R, et al. Prognostic factors for mortality, intensive care unit and hospital admission due to SARS-CoV-2: a systematic review and metaanalysis of cohort studies in Europe. Vol. 31, European Respiratory Review. European Respiratory Society; 2022.

37. Metlay JP, Waterer GW, Long AC, Anzueto A, Brozek J, Crothers K, et al. Diagnosis and treatment of adults with community-acquired pneumonia. Am J Respir Crit Care Med. 2019 Oct 1;200(7):E45–67.

38. Armstrong CAFP. Community-Acquired Pneumonia: Updated Recommendations from the ATS and IDSA. Am Fam Physician. 2020;102(2):121–4.

39. National Institute for Health and Care Excellence (NICE). Pneumonia in adults: diagnosis and management (Clinical guideline 191) [Internet]. 2014. Available from: www.nice.org.uk/guidance/cg191

40. Verheij T, Hopstaken R, Prins J, Salomé P, Bindels P, Ponsioen B, et al. NHG-Standaard Acuut hoesten [Internet]. Nederlands Huisartsen Genootschap. 2020 [cited 2022 May 25]. Available from: https://richtlijnen.nhg.org/standaarden/acuut-hoesten#volledige-tekst

41. McNally M, Curtain J, O'Brien KK, Dimitrov BD, Fahey T. Validity of British Thoracic Society guidance (the CRB-65 rule) for predicting the severity of pneumonia in general practice:

For peer review only - http://bmjopen.bmj.com/site/about/guidelines.xhtml

Systematic review and meta-analysis. British Journal of General Practice. 2010;60(579):423–33.

42. van Calster B, McLernon DJ, van Smeden M, Wynants L, Steyerberg EW, Bossuyt P, et al. Calibration: The Achilles heel of predictive analytics. BMC Med. 2019 Dec 16;17(1).

43. van Calster B, Nieboer D, Vergouwe Y, de Cock B, Pencina MJ, Steyerberg EW. A calibration hierarchy for risk models was defined: From utopia to empirical data. J Clin Epidemiol. 2016 Jun 1;74:167–76.

For peer review only

For peer review only - http://bmjopen.bmj.com/site/about/guidelines.xhtml

Tables

Table 1. Study characteristics of included prognostic factor studies

Study	*Source of data*	*Country*	*Setting*	*Study population*	*Participants, n*	*Included episodes*	*Age, mean (SD) or median (IQR)*	*Male sex, %*	*Number of candidate predictors*	*Outcome[a]*	*Hospitalisation, n (%)*	*Mortality, n (%)*	*Total outcome events, n (%)*	*EPP*
Houston, 1997	Cohort, retrospective	US	Community-based	Elderly (≥65 years) LRTI patients	413	413	NR	38.0	19	30-day all-cause mortality	NR	44 (10.7)	44 (10.7)	2.3
Seppä, 2001	Cohort, prospective	FIN	Primary care	Elderly (≥65 years) patients with LRTI suggestive of pneumonia	950	950	Survival: 76 (IQR 71-81), death: 80 (IQR 73-86)	52	20	LRTI-related mortality within 30 days	NR	38 (4.0)	38 (4.0)	1.9
Hak, 2005	Cohort, retrospective	NL	Primary care	Patients ≥60 years with an LRTI	455	455	75 (SD 8.6)	45	18	Composite of: i) Dysregulation of diabetes, stroke, heart failure, MI, ii) hospitalisation, iii) all-cause mortality, all within 30 days	21 (4.6)	24 (5.3)	65 (14.3)	3.6
Winchester, 2009	Cohort, retrospective	UK	Primary care	Patients ≥1 year with an LRTI	151,088	151,088	54 (IQR 37)	42.9	17	Respiratory infection-related admission or mortality within 3 months	1,147 (0.8)	2,126 (1.4)	NR	67[b]
Van de Nadort, 2009	Cohort, retrospective	NL	Primary care	Elderly (≥80 years) LRTI patients	509	860	85.2 (no SD)	37.7	20	Hospitalisation or death within 30 days	NR	51 (5.9)	109 (12.7)	5.5
Myles, 2009	Cohort, retrospective	UK	Primary care	Patients ≥40 years with an LRTI	NR	3,681	NR	NR	4	All-cause mortality within 30 days	NR	905 (24.6)	905 (24.6)	226
Millett, 2015	Cohort, retrospective	UK	Primary care	Elderly (≥65 years) CAP patients	39,211	43,576	81 (IQR 75-87)	47	40	Hospital admission within 28 days	33,321 (76.5)	NR	33,321 (76.5)	833

For peer review only - http://bmjopen.bmj.com/site/about/guidelines.xhtml

Moore, 2019	Cohort, prospective	UK	Primary care	Patients ≥16 years with acute cough attributed to LRTI	28,846	28,846	NR	40.7	29	LRTI-related hospitalisation, mortality or late-onset pneumonia within 2-30 days	196 (0.7)	29 (0.1)	325 (1.1)	11.2
Hamilton, 2021	Cohort, retrospective	UK	Primary care	Patients ≥50 years with pneumonia and history of lymphocyte test	28,556	28,556	76 (IQR 66-85)	49.5	14	28-day mortality	NR	2,544 (8.9)	2,544 (8.9)	182
Martínez-Redondo, 2021	Cohort, prospective	ESP	Primary care	Adults (≥18 years) with a COVID-19 pneumonia	161	161	53.0 (no SD)	50.9	4	Hospital admission, no time window reported	37 (23.0)	NR	37 (23.0)	9.3

Abbreviations: US, United States; FIN, Finland; NL, the Netherlands; UK, Unites Kingdom; ESP, Spain; LRTI, lower respiratory tract infection; CAP, community-acquired pneumonia; COVID-19, coronavirus disease 2019; SD, standard deviation; IQR, interquartile range; NR, not reported; MI, myocardial infarction; EPP, events per predictor.

[a] Time window of outcome is defined as days from first consultation within LRTI episode.

For peer review only - http://bmjopen.bmj.com/site/about/guidelines.xhtml

Table 2. Study characteristics of included prediction model studies

Study	*Type of study*	*Model(s)*	*Source of data*	*Country*	*Setting*	*Study population*	*Participants, n*	*Age, mean (SD)*	*Male sex, %*	*Outcome[c]*	*Hospitalisation, n (%)*	*Mortality, n (%)*	*Total outcome events, n (%)*	*EPP[d]*	*Method of validation*
Bont, 2007	D+V	New	Cohort, retrospective	NL	Primary care	Elderly (≥65) LRTI patients	D: 3,166, V: 2,465	75.5 (no SD)	D: 45, V: NR	Hospitalisation or all-cause mortality within 30 days	NR	D: 76 (2.4), V: 59 (2.4)	D: 274 (8.7), V: 178 (7.2)	13.7	Internal (random split-sample) and external
Bont, 2008	V	CRB-65	Cohort, prospective	NL	Primary care	Elderly (≥65) CAP patients	314	77.3 (no SD)	46	Mortality within 30 days	47 (15)	11 (3.5)	11 (3.5)	-	External
Ochoa-Gondar, 2011	V	PSI, CURB-65, CRB-65	Cohort, retrospective	ESP	Population-based	Elderly (≥65) patients with radiographically confirmed CAP	590	77.4 (7.6)	63.2	Mortality within 30 days	513 (86.9)	80 (13.6)	80 (13.6)	-	External
Francis, 2012	V	CRB-65	Cohort, prospective	13 countries[a]	Primary care	Adults (≥18) presenting with acute cough or suspected LRTI	339	49.3 (16.5)	NR	Hospitalisation within 28 days	10 (2.9)	NR	10 (2.9)	-	External
Ochoa-Gondar, 2013	D+V	CORB-75 (new), CRB-65	Cohort, retrospective	ESP	Population-based	Elderly (≥65) patients with radiographically confirmed CAP	D: 350, V: 260	78.1 (7.7)	D: 62.6, V: 59.2	Mortality within 30 days	D: 282 (80.6), V: 206 (79.2)	D: 46 (13.1), V: 34 (13.1)	D: 46 (13.1), V: 34 (13.1)	3.3	Internal-external (temporal, CORB-75), external (CRB-65)
Bruyndonckx, 2018	D (RISCC85) + V (all models)	RISCC85 (new), PSI (stage I), CRB, CRB-65, CURB, CURB-65	Cohort, prospective	6 countries[b]	Primary care	Adults (≥18) with symptoms suggestive of LRTI	2,604	50 (17)	40	Re-consultation with new/worsened symptoms or hospitalisation within 28 days	11 (0.5)	0 (0)	521 (20.0)	6.4	Internal (random split sample, RISCC85), external (other models)
Moore, 2019	D+V	New	Cohort, prospective	UK	Primary care	Patients ≥16 years with acute cough attributed to LRTI	28,846	NR	40.7	Hospitalisation, mortality, or clinical diagnosis of pneumonia within 2-30 days	196 (0.7)	29 (0.1)	325 (1.1)	11.2	Internal (bootstrapping)

[a] Belgium, Finland, Germany, Hungary, Italy, the Netherlands, Norway, Poland, Slovakia, Spain, Sweden, the United Kingdom.

For peer review only - http://bmjopen.bmj.com/site/about/guidelines.xhtml

[b] Belgium, Germany, the Netherlands, Poland, Spain, the United Kingdom.
[c] Time window of outcome is defined as days from first consultation.
[d] Only provided for newly developed prediction models and based on derivation cohort.
Abbreviations: D, development; V, validation; NL, the Netherlands; ESP, Spain; UK, United Kingdom; LRTI, lower respiratory tract infection; CAP, community acquired pneumonia; SD, standard deviation; NR, not reported; EPP, events per predictor.

For peer review only

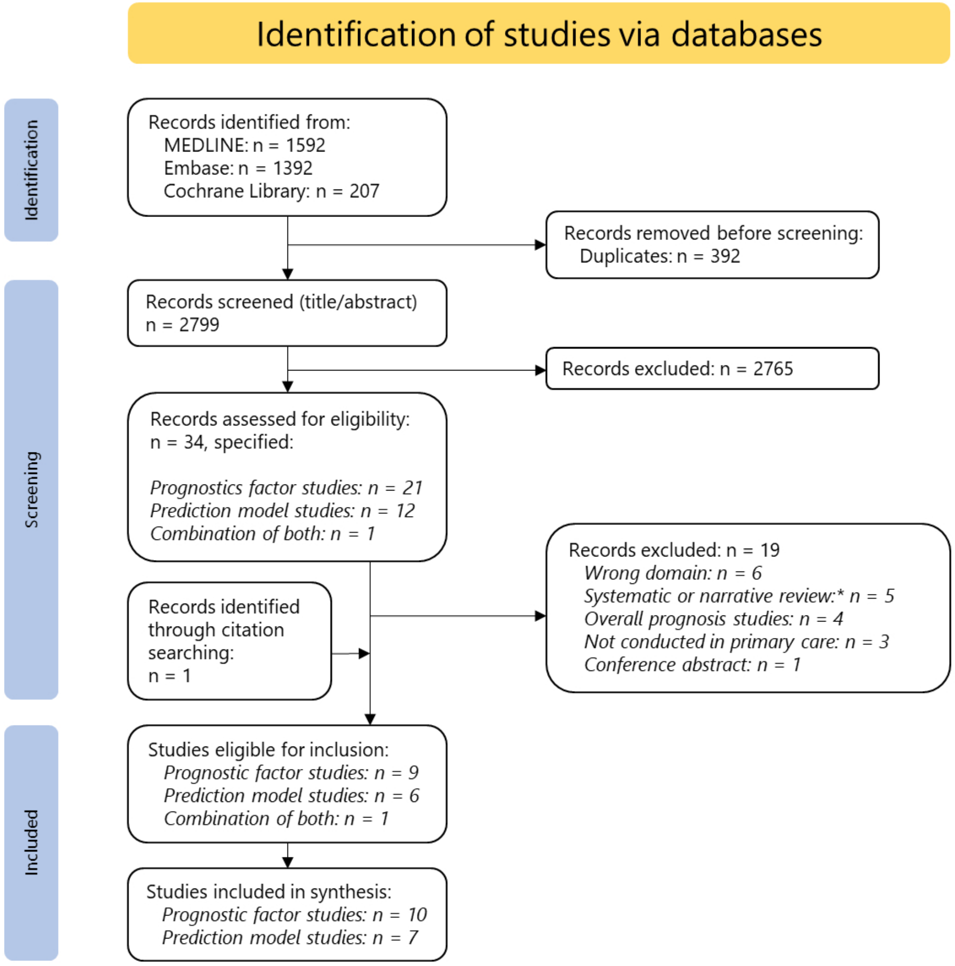

Figure 1. Flow diagram of search results.

Legend:
* Systematic reviews have been screened for eligible primary studies.

184x182mm (150 x 150 DPI)

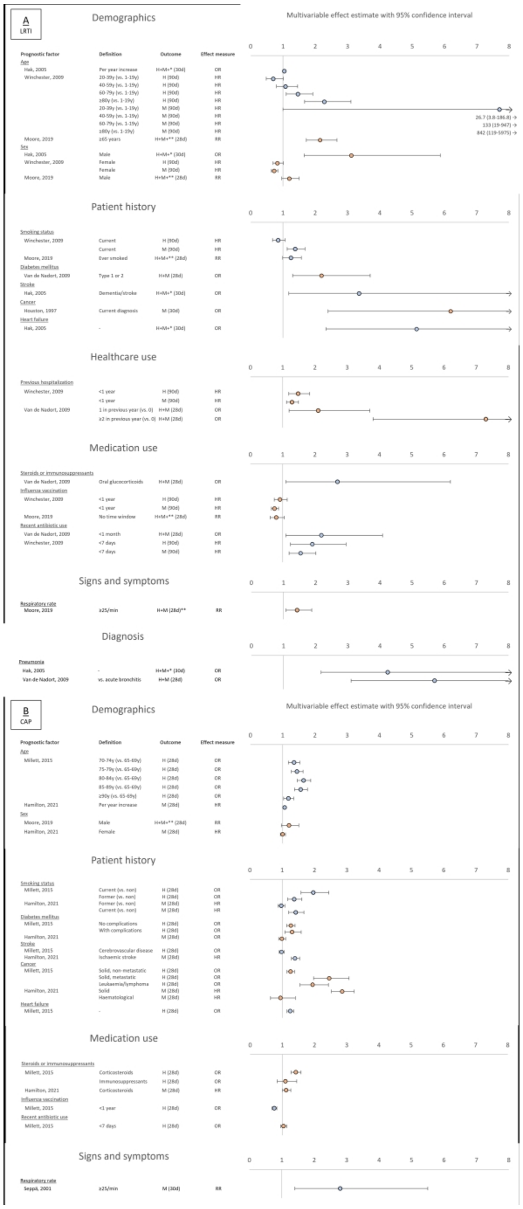

Figure 2. Overview of most promising candidate predictors for hospitalisation or mortality within 90 days based on multivariable analysis

Legend:
A. Promising candidate predictors based on studies including LRTI patients. B. Promising candidate predictors based on studies including community-acquired pneumonia patients.
* Composite outcome also includes dysregulation of diabetes, stroke, heart failure, MI.
** Composite outcome also includes late onset pneumonia.
Abbreviations: LRTI, lower respiratory tract infection; CAP, community-acquired pneumonia; H, hospitalisation; M, mortality; OR, odds ratio; HR, hazard ratio; RR, risk ratio; min, minute.

233x540mm (300 x 300 DPI)

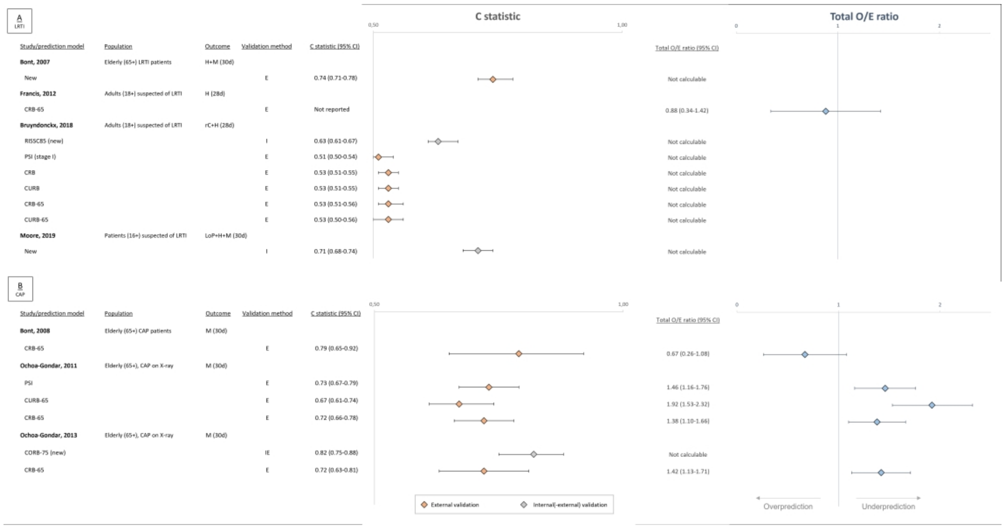

Figure 3. Overview of discrimination and calibration performance measures of the included prediction models

Legend:
A. Performance of prediction models evaluated in LRTI patients. B. Performance of prediction models evaluated in community-acquired pneumonia patients.
Abbreviations: LRTI, lower respiratory tract infection; CAP, community-acquired pneumonia; H, hospitalisation; M, mortality; rC, reconsultation; LoP, late onset pneumonia; E, external; IE, internal-external; I, internal; CI, confidence interval; O/E, observed over expected.

557x290mm (330 x 330 DPI)

PRISMA 2020 Checklist

Section and Topic	Item #	Checklist item	Location where item is reported
**TITLE**			
Title	1	Identify the report as a systematic review.	1
**ABSTRACT**			
Abstract	2	See the PRISMA 2020 for Abstracts checklist.	3
**INTRODUCTION**			
Rationale	3	Describe the rationale for the review in the context of existing knowledge.	6
Objectives	4	Provide an explicit statement of the objective(s) or question(s) the review addresses.	6
**METHODS**			
Eligibility criteria	5	Specify the inclusion and exclusion criteria for the review and how studies were grouped for the syntheses.	7
Information sources	6	Specify all databases, registers, websites, organisations, reference lists and other sources searched or consulted to identify studies. Specify the date when each source was last searched or consulted.	7
Search strategy	7	Present the full search strategies for all databases, registers and websites, including any filters and limits used.	S1
Selection process	8	Specify the methods used to decide whether a study met the inclusion criteria of the review, including how many reviewers screened each record and each report retrieved, whether they worked independently, and if applicable, details of automation tools used in the process.	7
Data collection process	9	Specify the methods used to collect data from reports, including how many reviewers collected data from each report, whether they worked independently, any processes for obtaining or confirming data from study investigators, and if applicable, details of automation tools used in the process.	7, 8
Data items	10a	List and define all outcomes for which data were sought. Specify whether all results that were compatible with each outcome domain in each study were sought (e.g. for all measures, time points, analyses), and if not, the methods used to decide which results to collect.	7, 8
	10b	List and define all other variables for which data were sought (e.g. participant and intervention characteristics, funding sources). Describe any assumptions made about any missing or unclear information.	8
Study risk of bias assessment	11	Specify the methods used to assess risk of bias in the included studies, including details of the tool(s) used, how many reviewers assessed each study and whether they worked independently, and if applicable, details of automation tools used in the process.	8
Effect measures	12	Specify for each outcome the effect measure(s) (e.g. risk ratio, mean difference) used in the synthesis or presentation of results.	7, 8
Synthesis methods	13a	Describe the processes used to decide which studies were eligible for each synthesis (e.g. tabulating the study intervention characteristics and comparing against the planned groups for each synthesis (item #5)).	7, 8
	13b	Describe any methods required to prepare the data for presentation or synthesis, such as handling of missing summary statistics, or data conversions.	7, 8
	13c	Describe any methods used to tabulate or visually display results of individual studies and syntheses.	8, 9
	13d	Describe any methods used to synthesize results and provide a rationale for the choice(s). If meta-analysis was performed, describe the model(s), method(s) to identify the presence and extent of statistical heterogeneity, and software package(s) used.	8, 9
	13e	Describe any methods used to explore possible causes of heterogeneity among study results (e.g. subgroup analysis, meta-regression).	N/A
	13f	Describe any sensitivity analyses conducted to assess robustness of the synthesized results.	N/A
Reporting bias assessment	14	Describe any methods used to assess risk of bias due to missing results in a synthesis (arising from reporting biases).	N/A
Certainty	15	Describe any methods used to assess certainty (or confidence) in the body of evidence for an outcome.	N/A

For peer review only - http://bmjopen.bmj.com/site/about/guidelines.xhtml

PRISMA 2020 Checklist

Section and Topic	Item #	Checklist item	Location where item is reported
assessment			
**RESULTS**			
Study selection	16a	Describe the results of the search and selection process, from the number of records identified in the search to the number of studies included in the review, ideally using a flow diagram.	10, Figure 1
	16b	Cite studies that might appear to meet the inclusion criteria, but which were excluded, and explain why they were excluded.	N/A
Study characteristics	17	Cite each included study and present its characteristics.	10, Table 2-3
Risk of bias in studies	18	Present assessments of risk of bias for each included study.	11, Figure S2-3
Results of individual studies	19	For all outcomes, present, for each study: (a) summary statistics for each group (where appropriate) and (b) an effect estimate and its precision (e.g. confidence/credible interval), ideally using structured tables or plots.	11-13, Figure 2-3, Figure S4-5
Results of syntheses	20a	For each synthesis, briefly summarise the characteristics and risk of bias among contributing studies.	11
	20b	Present results of all statistical syntheses conducted. If meta-analysis was done, present for each the summary estimate and its precision (e.g. confidence/credible interval) and measures of statistical heterogeneity. If comparing groups, describe the direction of the effect.	11-13, Figure 2-3, Figure S4-5
	20c	Present results of all investigations of possible causes of heterogeneity among study results.	N/A
	20d	Present results of all sensitivity analyses conducted to assess the robustness of the synthesized results.	N/A
Reporting biases	21	Present assessments of risk of bias due to missing results (arising from reporting biases) for each synthesis assessed.	N/A
Certainty of evidence	22	Present assessments of certainty (or confidence) in the body of evidence for each outcome assessed.	N/A
**DISCUSSION**			
Discussion	23a	Provide a general interpretation of the results in the context of other evidence.	14, 15
	23b	Discuss any limitations of the evidence included in the review.	16
	23c	Discuss any limitations of the review processes used.	16
	23d	Discuss implications of the results for practice, policy, and future research.	17
**OTHER INFORMATION**			
Registration and protocol	24a	Provide registration information for the review, including register name and registration number, or state that the review was not registered.	3, 7
	24b	Indicate where the review protocol can be accessed, or state that a protocol was not prepared.	3, 7
	24c	Describe and explain any amendments to information provided at registration or in the protocol.	N/A
Support	25	Describe sources of financial or non-financial support for the review, and the role of the funders or sponsors in the review.	18
Competing interests	26	Declare any competing interests of review authors.	18
Availability of data, code and other materials	27	Report which of the following are publicly available and where they can be found: template data collection forms; data extracted from included studies; data used for all analyses; analytic code; any other materials used in the review.	18

For peer review only
For peer review only - http://bmjopen.bmj.com/site/about/guidelines.xhtml

PRISMA 2020 Checklist

From: Page MJ, McKenzie JE, Bossuyt PM, Boutron I, Hoffmann TC, Mulrow CD, et al. The PRISMA 2020 statement: an updated guideline for reporting systematic reviews. BMJ 2021;372:n71. doi: 10.1136/bmj.n71

For more information, visit: http://www.prisma-statement.org/

For peer review only

PROSPERO
International prospective register of systematic reviews

National Institute for Health Research

University of York
Centre for Reviews and Dissemination

Systematic review

A list of fields that can be edited in an update can be found here

1. * Review title.

Give the title of the review in English

Prognosis of lower respiratory tract infections in adults presenting to primary care: a systematic review of prognostic factors and prediction models

2. Original language title.

For reviews in languages other than English, give the title in the original language. This will be displayed with the English language title.

3. * Anticipated or actual start date.

Give the date the systematic review started or is expected to start.

17/06/2022

4. * Anticipated completion date. [1 change]

Give the date by which the review is expected to be completed.

01/04/2023

5. * Stage of review at time of this submission. [3 changes]

This field uses answers to initial screening questions. It cannot be edited until after registration.

Tick the boxes to show which review tasks have been started and which have been completed.

Update this field each time any amendments are made to a published record.

The review has not yet started: No

For peer review only

PROSPERO
International prospective register of systematic reviews

National Institute for Health Research

Review stage	Started	Completed
Preliminary searches	Yes	Yes
Piloting of the study selection process	Yes	Yes
Formal screening of search results against eligibility criteria	Yes	Yes
Data extraction	Yes	Yes
Risk of bias (quality) assessment	Yes	Yes
Data analysis	Yes	Yes

Provide any other relevant information about the stage of the review here.

6. * Named contact.

The named contact is the guarantor for the accuracy of the information in the register record. This may be any member of the review team.

Merijn Rijk

Email salutation (e.g. "Dr Smith" or "Joanne") for correspondence:

Merijn Rijk

7. * Named contact email.

Give the electronic email address of the named contact.

m.h.rijk@umcutrecht.nl

8. Named contact address

Give the full institutional/organisational postal address for the named contact.

P.O. Box 85500, 3508 GA Utrecht

9. Named contact phone number.

Give the telephone number for the named contact, including international dialling code.

0031647424873

10. * Organisational affiliation of the review.

Full title of the organisational affiliations for this review and website address if available. This field may be

For peer review only

completed as 'None' if the review is not affiliated to any organisation.

Julius Center for Health Sciences and Primary Care, UMC Utrecht

Organisation web address:

11. * Review team members and their organisational affiliations. [1 change]

Give the personal details and the organisational affiliations of each member of the review team. Affiliation refers to groups or organisations to which review team members belong. **NOTE: email and country now MUST be entered for each person, unless you are amending a published record.**

Mr Merijn Rijk. Julius Center for Health Sciences and Primary Care, UMC Utrecht
Mr Teun van den Berg. Julius Center for Health Sciences and Primary Care, UMC Utrecht
Dr Tamara Platteel. Julius Center for Health Sciences and Primary Care, UMC Utrecht
Dr Roderick Venekamp. Julius Center for Health Sciences and Primary Care, UMC Utrecht
Dr Maarten van Smeden. Julius Center for Health Sciences and Primary Care, UMC Utrecht
Dr Geert-Jan Geersing. Julius Center for Health Sciences and Primary Care, UMC Utrecht
Professor Frans Rutten. Julius Center for Health Sciences and Primary Care, UMC Utrecht
Dr Monika Hollander. Julius Center for Health Sciences and Primary Care, UMC Utrecht
Professor Paul Little. University of Southampton
Dr Bert Dalmolen.

12. * Funding sources/sponsors.

Details of the individuals, organizations, groups, companies or other legal entities who have funded or sponsored the review.

The Netherlands Organisation for Health Research and Development (ZonMw), Huisartsgeneeskunde 2021,

no. 08391052110003

Grant number(s)

State the funder, grant or award number and the date of award

ZonMw, General Practice and Elderly Care grant, round of 2021

13. * Conflicts of interest.

List actual or perceived conflicts of interest (financial or academic).

None

14. Collaborators.

Give the name and affiliation of any individuals or organisations who are working on the review but who are not listed as review team members. **NOTE: email and country must be completed for each person, unless you are amending a published record.**

For peer review only

PROSPERO
International prospective register of systematic reviews
National Institute for Health Research

15. * Review question. [1. change]

State the review question(s) clearly and precisely. It may be appropriate to break very broad questions down into a series of related more specific questions. Questions may be framed or refined using PI(E)COS or similar where relevant.

1) What are prognostic factors for a complicated trajectory (i.e. hospitalization or death) within 90 days in adult patients with lower respiratory tract infections (LRTI) presenting to primary care?

P: adult patients with LRTI

I: any patient or disease specific predictor

C: -

O: Hospitalisation or death

T1: prognostic factor present at first consultation of LRTI episode

T2: outcome within 90 days

S: primary care

2) What prediction models have been developed to estimate individual risk of hospitalization and/or death within 90 days in adult patients with a LRTI presenting to primary care?

P: adult patients with LRTI

I: any developed prediction model

C: -

O: Hospitalisation or death

T1: prediction at first consultation of LRTI episode

T2: outcome within 90 days

S: primary care

16. * Searches.

State the sources that will be searched (e.g. Medline). Give the search dates, and any restrictions (e.g. language or publication date). Do NOT enter the full search strategy (it may be provided as a link or attachment below.)

For peer review only

MEDLINE, Embase, and Cochrane Library. Restrictions: no restrictions in search strategy.

17. URL to search strategy.

Upload a file with your search strategy, or an example of a search strategy for a specific database, (including the keywords) in pdf or word format. In doing so you are consenting to the file being made publicly accessible. Or provide a URL or link to the strategy. Do NOT provide links to your search **results**.

https://www.crd.york.ac.uk/PROSPEROFILES/341233_STRATEGY_20220628.pdf

Alternatively, upload your search strategy to CRD in pdf format. Please note that by doing so you are consenting to the file being made publicly accessible.

Do not make this file publicly available until the review is complete

18. * Condition or domain being studied.

Give a short description of the disease, condition or healthcare domain being studied in your systematic review.

Prognosis of lower respiratory tract infections in adults presenting to primary care

19. * Participants/population.

Specify the participants or populations being studied in the review. The preferred format includes details of both inclusion and exclusion criteria.

Adults with lower respiratory tract infections presenting to primary care

20. * Intervention(s), exposure(s).

Give full and clear descriptions or definitions of the interventions or the exposures to be reviewed. The preferred format includes details of both inclusion and exclusion criteria.

For review question 1): any patient or disease specific prognostic factor
For review question 2): any developed or updated prediction model applicable to a primary care setting

21. * Comparator(s)/control.

Where relevant, give details of the alternatives against which the intervention/exposure will be compared (e.g. another intervention or a non-exposed control group). The preferred format includes details of both inclusion and exclusion criteria.

Not relevant

22. * Types of study to be included.

Give details of the study designs (e.g. RCT) that are eligible for inclusion in the review. The preferred format includes both inclusion and exclusion criteria. If there are no restrictions on the types of study, this should be stated.

For review question 1): prognostic factor studies
For review question 2): model development and validation studies

For peer review only

PROSPERO
International prospective register of systematic reviews

National Institute for Health Research

23. Context.

Give summary details of the setting or other relevant characteristics, which help define the inclusion or exclusion criteria.

For review question 1):

- Prognostic factor studies conducted within (or applicable to) a primary care setting

For review question 2):

- Model development and/or validation studies conducted within (or applicable to) a primary care setting

- Validation or updating of secondary care derived prediction models in primary care

24. * Main outcome(s).

Give the pre-specified main (most important) outcomes of the review, including details of how the outcome is defined and measured and when these measurement are made, if these are part of the review inclusion criteria.

Hospitalisation or (all-cause) death within 90 days after initial presentation to primary care

Measures of effect

Please specify the effect measure(s) for you main outcome(s) e.g. relative risks, odds ratios, risk difference, and/or 'number needed to treat.

For review question 1): prognostic effect measures (odds ratios and hazard ratios), both unadjusted and adjusted

For review question 2): data on discrimination (C statistic) and calibration (O:E ratio, intercept, slope)

25. * Additional outcome(s).

List the pre-specified additional outcomes of the review, with a similar level of detail to that required for main outcomes. Where there are no additional outcomes please state 'None' or 'Not applicable' as appropriate to the review

Not applicable

Measures of effect

Please specify the effect measure(s) for you additional outcome(s) e.g. relative risks, odds ratios, risk difference, and/or 'number needed to treat.

26. * Data extraction (selection and coding).

Describe how studies will be selected for inclusion. State what data will be extracted or obtained. State how this will be done and recorded.

Study selection:

1) Clinical domain, outcome, and setting match PICOTS, study design: prognostic factor study or prognostic model development or validation study

For peer review only

- Exclusion: full-text not available, language other than English or Dutch

2) Screening for eligible studies by two researchers, independently and blinded. Screening process: 1) title/abstract, 2) full-text, 3) backward and forward citation searching of included studies.

3) Disagreements resolved through discussion and, if necessary, after consultation of third researcher (supervisor)

4) Software system: de-duplication of yielded studies and title/abstract screening using Rayyan (online application)

Data extraction:

1) Data extraction based on CHARMS (prediction models) and CHARMS-PF (prognostic factor studies) checklists

2) Extraction of data by two researchers: extraction of data by researcher 1, check of extracted data by researcher 2

3) Disagreements will be resolved through discussion and, if neccesary, after consultation of a third researcher (supervisor)

4) Missing data on measurements of effect will be either calculated from raw data if possible or requested from researchers of the primary study

For peer review only

27. * Risk of bias (quality) assessment.

State which characteristics of the studies will be assessed and/or any formal risk of bias/quality assessment tools that will be used.

For risk of bias assessment the following formal tools will be used:

- For review question 1): QUIPS

- For review question 2): PROBAST

28. * Strategy for data synthesis.

[1 change]

Describe the methods you plan to use to synthesise data. This **must not be generic text** but should be **specific to your review** and describe how the proposed approach will be applied to your data. If meta-analysis is planned, describe the models to be used, methods to explore statistical heterogeneity, and software package to be used.

For review question 1):

PROSPERO
International prospective register of systematic reviews

National Institute for Health Research

Data synthesis will not be performed for prognostic factors due to 1) the broad search for 'any' prognostic factor and 2) the anticipated heterogeneity of the domain and prognostic factor definition. Effect measures (unadjusted and adjusted) of prognostic factors will be summarised in a forest plot.

For review question 2):

Data synthesis will not be performed. Prediction models will be evaluated and compared based on:

- Risk of bias in the development (using PROBAST tool)

- Applicability to the clinical patient and setting (PICOTS and review question)

- Performance of the model (based on the performance measures mentioned in field #24)

- Method of validation (internal, internal-external, external).

Based on these criteria conclusions will be drawn with regard to the usefulness of the prediction models to clinical practice. This will provide guidance for clinicians to choose the appropriate tool (if any of the prediction models proves to be robust) for prediction of hospitalization or death in LRTI patients.

29. * Analysis of subgroups or subsets.

State any planned investigation of ‘subgroups’. Be clear and specific about which type of study or participant will be included in each group or covariate investigated. State the planned analytic approach.

Studies for both review questions will be stratified with regard to data collection on (possible) Coronavirus disease 2019 (COVID-19) patients, to assess any COVID-19 specific effect on results:

- Non-COVID-19 data (data collection up to and including 2019 and data on (explicitly stated) non-COVID-19 patients collected from 2020 onwards)

- COVID-19 data (data collection from 2020 onwards on (possible) COVID-19 patients)

30. * Type and method of review.

Select the type of review, review method and health area from the lists below.

Type of review

Cost effectiveness

No

Diagnostic

No

Epidemiologic

No

Individual patient data (IPD) meta-analysis

No

Intervention

No

Living systematic review

No

Meta-analysis

No

Methodology

No

Narrative synthesis

No

Network meta-analysis

No

Pre-clinical

No

Prevention

No

Prognostic

Yes

Prospective meta-analysis (PMA)

No

Review of reviews

No

Service delivery

No

Synthesis of qualitative studies

No

Systematic review

Yes

Other

No

Health area of the review

Alcohol/substance misuse/abuse

No

Blood and immune system

For peer review only

No

Cancer

No

Cardiovascular

No

Care of the elderly

No

Child health

No

Complementary therapies

No

COVID-19

Yes

For COVID-19 registrations please tick all categories that apply. Doing so will enable your record to appear in area-specific searches

Chinese medicine
Diagnosis
Epidemiological
Genetics
Health impacts
Immunity
Long COVID
Mental health
PPE
Prognosis
Public health intervention
Rehabilitation
Service delivery
Transmission
Treatments
Vaccines
Other

Crime and justice

No

Dental

No

Digestive system

No

Ear, nose and throat

No

For peer review only

Education
No

Endocrine and metabolic disorders
No

Eye disorders
No

General interest
No

Genetics
No

Health inequalities/health equity
No

Infections and infestations
Yes

International development
No

Mental health and behavioural conditions
No

Musculoskeletal
No

Neurological
No

Nursing
No

Obstetrics and gynaecology
No

Oral health
No

Palliative care
No

Perioperative care
No

Physiotherapy
No

Pregnancy and childbirth
No

Public health (including social determinants of health)

For peer review only

No

Rehabilitation

No

Respiratory disorders

No

Service delivery

No

Skin disorders

No

Social care

No

Surgery

No

Tropical Medicine

No

Urological

No

Wounds, injuries and accidents

No

Violence and abuse

No

31. Language.

Select each language individually to add it to the list below, use the bin icon to remove any added in error.

English

There is not an English language summary

32. * Country.

Select the country in which the review is being carried out. For multi-national collaborations select all the countries involved.

Netherlands

33. Other registration details.

Name any other organisation where the systematic review title or protocol is registered (e.g. Campbell, or The Joanna Briggs Institute) together with any unique identification number assigned by them. If extracted data will be stored and made available through a repository such as the Systematic Review Data Repository (SRDR), details and a link should be included here. If none, leave blank.

For peer review only

PROSPERO
International prospective register of systematic reviews

National Institute for Health Research

34. Reference and/or URL for published protocol.

If the protocol for this review is published provide details (authors, title and journal details, preferably in Vancouver format)

Add web link to the published protocol.

Or, upload your published protocol here in pdf format. Note that the upload will be publicly accessible.

No I do not make this file publicly available until the review is complete

Please note that the information required in the PROSPERO registration form must be completed in full even if access to a protocol is given.

35. Dissemination plans.

Do you intend to publish the review on completion?

Yes

Give brief details of plans for communicating review findings.?

36. Keywords.

Give words or phrases that best describe the review. Separate keywords with a semicolon or new line. Keywords help PROSPERO users find your review (keywords do not appear in the public record but are included in searches). Be as specific and precise as possible. Avoid acronyms and abbreviations unless these are in wide use.

Systematic review; prognosis; prognostic factor; prediction model; lower respiratory tract infection; adults; primary care; general practice; hospitalisation; mortality; complications.

37. Details of any existing review of the same topic by the same authors.

If you are registering an update of an existing review give details of the earlier versions and include a full bibliographic reference, if available.

38. * Current review status. [1. change]

Update review status when the review is completed and when it is published.New registrations must be ongoing so this field is not editable for initial submission.

Please provide anticipated publication date

For peer review only

PROSPERO
International prospective register of systematic reviews

Review_Completed_not_published

39. Any additional information. [1 change]

Provide any other information relevant to the registration of this review.

Additional information added after initial submission

- Included PICOTS components for review questions (field #15)

- Elaboration upon the assessment of prediction models and how these results help clinicians (GPs) to choose the appropriate tool (if any) for prediction of hospitalization or death in LRTI patients (field #28)

40. Details of final report/publication(s) or preprints if available.

Leave empty until publication details are available OR you have a link to a preprint (NOTE: this field is not editable for initial submission). List authors, title and journal details preferably in Vancouver format.

Give the link to the published review or preprint.

For peer review only

BMJ Open

Prognostic factors and prediction models for hospitalisation and all-cause mortality in adults presenting to primary care with a lower respiratory tract infection: a systematic review

Journal:	*BMJ Open*
Manuscript ID	bmjopen-2023-075475.R1
Article Type:	Original research
Date Submitted by the Author:	11-Dec-2023
Complete List of Authors:	Rijk, Merijn; University Medical Centre Utrecht, Julius Center for Health Sciences and Primary Care<br>Platteel, Tamara ; University Medical Centre Utrecht, Julius Center for Health Sciences and Primary Care<br>van den Berg, Teun; University Medical Centre Utrecht, Julius Center for Health Sciences and Primary Care<br>Geersing, Geert-Jan; Julius Center for Health Sciences and Primary Care, Department of General Practice<br>Little, Paul; University of Southampton, Primary Care and Population Science;<br>Rutten, Frans; Julius Centre for Health Sciences and Primary Care, University Medical Centre Utrecht, Department of General Practice<br>van Smeden, Maarten; University Medical Center Utrecht, Julius Center for Health Sciences and Primary Care<br>Venekamp , Roderick ; University Medical Center, Utrecht, Julius Center for Health Sciences and Primary Care
**Primary Subject Heading**:	Respiratory medicine
Secondary Subject Heading:	General practice / Family practice
Keywords:	Respiratory infections < THORACIC MEDICINE, Primary Care < Primary Health Care, Prognosis, Systematic Review

SCHOLARONE™
Manuscripts

I, the Submitting Author has the right to grant and does grant on behalf of all authors of the Work (as defined in the below author licence), an exclusive licence and/or a non-exclusive licence for contributions from authors who are: i) UK Crown employees; ii) where BMJ has agreed a CC-BY licence shall apply, and/or iii) in accordance with the terms applicable for US Federal Government officers or employees acting as part of their official duties; on a worldwide, perpetual, irrevocable, royalty-free basis to BMJ Publishing Group Ltd ("BMJ") its licensees and where the relevant Journal is co-owned by BMJ to the co-owners of the Journal, to publish the Work in this journal and any other BMJ products and to exploit all rights, as set out in our licence.

The Submitting Author accepts and understands that any supply made under these terms is made by BMJ to the Submitting Author unless you are acting as an employee on behalf of your employer or a postgraduate student of an affiliated institution which is paying any applicable article publishing charge ("APC") for Open Access articles. Where the Submitting Author wishes to make the Work available on an Open Access basis (and intends to pay the relevant APC), the terms of reuse of such Open Access shall be governed by a Creative Commons licence – details of these licences and which Creative Commons licence will apply to this Work are set out in our licence referred to above.

Other than as permitted in any relevant BMJ Author's Self Archiving Policies, I confirm this Work has not been accepted for publication elsewhere, is not being considered for publication elsewhere and does not duplicate material already published. I confirm all authors consent to publication of this Work and authorise the granting of this licence.

For peer review only

Title page

Title

Prognostic factors and prediction models for hospitalisation and all-cause mortality in adults presenting to primary care with a lower respiratory tract infection: a systematic review

Authors

Merijn H. Rijk[1], MD; Tamara N. Platteel[1], MD PhD; Teun M. C. van den Berg[1], MD; Geert-Jan Geersing[1], MD PhD; Paul Little[2], MD PhD; Frans H. Rutten[1], MD PhD; Maarten van Smeden[3]*, PhD; Roderick P. Venekamp[1]*, MD PhD

Affiliations

[1] Department of General Practice & Nursing Science, Julius Center for Health Sciences and Primary Care, University Medical Center Utrecht, Utrecht University, Utrecht, the Netherlands

[2] Primary Care Research Centre, Primary Care Population Sciences and Medical Education Unit, University of Southampton, Southampton, United Kingdom

[3] Department of Epidemiology & Health Economics, Julius Center for Health Sciences and Primary Care, University Medical Center Utrecht, Utrecht University, Utrecht, the Netherlands

* MvS and RPV contributed equally to this work.

Corresponding author

Merijn H. Rijk, MD

For peer review only - http://bmjopen.bmj.com/site/about/guidelines.xhtml

Department of General Practice & Nursing Science, Julius Center for Health Sciences and Primary Care, University Medical Center Utrecht, Utrecht University, Utrecht, the Netherlands

Universiteitsweg 100, 3584 CG Utrecht

Phone: 0031 88 75 681 81

Email: m.h.rijk@umcutrecht.nl

For peer review only

Prior presentation(s)

None

Word count

Abstract: 300

Main text: 3385

Number of tables, figures, and appendices

Tables: 2

Figures: 3

Appendices: 7

For peer review only - http://bmjopen.bmj.com/site/about/guidelines.xhtml

Abstract

Objective To identify and synthesize relevant existing prognostic factors (PF) and prediction models (PM) for hospitalisation and all-cause mortality within 90 days in primary care patients with acute lower respiratory tract infections (LRTI).

Design Systematic review.

Methods Systematic searches of MEDLINE, Embase, and the Cochrane Library were performed. All PF and PM studies on risk of hospitalisation or all-cause mortality within 90 days in adult primary care LRTI patients were included. Risk of bias was assessed using the QUIPS and PROBAST tools for PF and PM studies, respectively. Results of included PF and PM studies were descriptively summarised.

Results Of 2,799 unique records identified, 16 were included: nine PF studies, six PM studies, and one combination of both. Risk of bias was judged high for all studies, mainly due to limitations in the analysis domain. Based on reported multivariable associations in PF studies, increasing age, sex, current smoking, diabetes, a history of stroke, cancer, or heart failure, previous hospitalisation, influenza vaccination (negative association), current use of systemic corticosteroids, recent antibiotic use, respiratory rate ≥25/minute, and diagnosis of pneumonia were identified as most promising candidate predictors. One newly developed PM was externally validated (c statistic 0.74, 95% confidence interval [CI] 0.71 to 0.78) whereas the previously hospital-derived CRB-65 was externally validated in primary care in five studies (c statistic ranging from 0.72 [95% CI 0.63 to 0.81] to 0.79 [95% CI 0.65 to 0.92]). None of the PM studies reported measures of model calibration.

Conclusions Implementation of existing models for individualised risk prediction of 90-day hospitalisation or mortality in primary care LRTI patients in everyday practice is hampered by incomplete assessment of model performance. The identified candidate predictors provide useful information for clinicians and warrant consideration when developing or updating PMs using state-of-the-art development and validation techniques.

PROSPERO registration number CRD42022341233

For peer review only - http://bmjopen.bmj.com/site/about/guidelines.xhtml

Strengths and limitations of this study

- Both prognostic factors and prediction models were covered in this comprehensive systematic review.
- The systematic search syntax included a validated prognostic search string to identify all relevant prognostic literature.
- Prevailing guidelines were followed for data extraction (CHARMS-PF and CHARMS), quality assessment (QUIPS, PROBAST, and GRADE) and reporting (PRISMA).
- Substantial between-study heterogeneity hampered pooling of the results in meta-analysis.

For peer review only

Keywords

Lower respiratory tract infections; primary care; general practice; prognosis; prognostic factor; prediction model; systematic review

Abbreviations

LRTI	Lower respiratory tract infections
CAP	Community-acquired pneumonia
GP	General practitioner
PSI	Pneumonia severity index
PF	Prognostic factor
PM	Prediction model
CHARMS	Checklist for Critical Appraisal and Data Extraction for Systematic Reviews of Prediction Modelling Studies
QUIPS	Quality in Prognostic Studies
PROBAST	Prediction Model Risk Of Bias Assessment Tool
GRADE	Grading of Recommendations, Assessment, Development, and Evaluations
CI	Confidence interval
O/E	Observed over expected
COVID-19	Coronavirus disease 2019
NICE	National Institute for Health and Care Excellence
ATS	American Thoracic Society
IDSA	Infectious Diseases Society of America

For peer review only

For peer review only - http://bmjopen.bmj.com/site/about/guidelines.xhtml

Introduction

Acute lower respiratory tract infections (LRTI) are common in primary care.(1,2) Uncomplicated LRTI episodes generally have a favourable natural course in otherwise healthy adults, and antibiotic treatment with amoxicillin confers only little benefit in terms of earlier symptom resolution, both overall and in higher-risk subgroups of patients.(3,4)(5) The clinical spectrum of LRTI patients, however, is heterogenous in terms of patient and disease specific characteristics, and thus the risk of hospitalisation and death varies substantially. For example, observational evidence suggests that adverse outcomes such as hospitalisation and death occur in fewer than 1% of patients with uncomplicated LRTI(5), whereas this risk is far more pronounced among those with community-acquired pneumonia (CAP).(6,7) However, diagnosing CAP in a primary care setting is notoriously challenging(8,9), leaving general practitioners (GP) with uncertainties how to identify higher-risk groups among those presenting with LRTI symptoms.

Nevertheless, identification of LRTI patients at highest risk of complications is pivotal as this might help GPs identify those in whom close follow-up or early (antibiotic) treatment is warranted. Currently, several scores exist to assist physicians in estimating risk of adverse outcomes in LRTI patients, such as the Pneumonia Severity Index (PSI) and CURB-65 (a prediction rule including confusion, blood urea nitrogen, respiratory rate, blood pressure, and age ≥65 years).(10,11) However, these prediction rules have been developed in secondary care and the inclusion of laboratory and/or radiographic features hampers applicability to outpatients. A modified version of the CURB-65 (CRB-65, not including blood urea nitrogen) has been proposed as primary care alternative, but its development in hospitalised patients raises uncertainty about its performance in outpatients.(10) An overview of existing primary care-specific prognostic factors (PF) and prediction models (PM) for adverse outcomes in LRTI patients could provide insight on (1) existing PFs or PMs suitable for use in clinical practice and (2) relevant PFs to include when developing a new or updating an existing model. We therefore aimed to synthesize existing knowledge on PFs and PMs for hospitalisation and mortality within 90 days in adult patients presenting to primary care with LRTI.

For peer review only - http://bmjopen.bmj.com/site/about/guidelines.xhtml

Methods

The protocol for this review was registered in PROSPERO (CRD42022341233) and the review was reported according to the PRISMA reporting guideline for systematic reviews.(12)

Searches and study selection

The electronic databases of MEDLINE, Embase, and the Cochrane Library were searched on 27 June 2022 for eligible studies. Keywords for 'respiratory tract infection', 'hospitalisation', 'death', 'adults' and 'primary care' were combined with a validated search string to identify relevant prognostic studies (Table S1).(13) Eligibility criteria were based on PICOTS guidance; we included all studies on adults with LRTI (patients) in which prognostic factors or prediction models (index) for 90-day hospitalisation or all-cause mortality (outcome) were assessed on the day of diagnosis (timing) in primary care (setting).(14,15) Non-English articles or those without full-text availability were excluded.

After de-duplication, two review authors (MHR, TMCB) independently reviewed titles/abstracts of unique records retrieved from the electronic databases against the eligibility criteria. Next, the same authors independently reviewed the full text of potentially relevant articles for eligibility. Any disagreements were resolved by discussion and, where necessary, by consulting a third reviewer (TNP). To increase the yield of potentially eligible articles, reference lists of the included articles were reviewed for eligibility. Studies assessing the prognostic value of PFs, developing a new PM, or externally validating a previously developed PM were eligible for inclusion.

Data collection

Data from eligible PF and PM studies were extracted by MHR using standardised forms based on the Checklist for Critical Appraisal and Data Extraction for Systematic Reviews of PF studies (CHARMS-PF) and PM studies (CHARMS), respectively.(14,16) Main outcomes of interest were hospitalisation or death within 90 days after initial LRTI consultation. For PF studies we

For peer review only - http://bmjopen.bmj.com/site/about/guidelines.xhtml

extracted odds ratios, hazard ratios, or risk ratios with accompanying 95% confidence intervals (CI) from both univariable and multivariable analyses. For those studies that did not report such estimates, these associations were calculated from crude data using Rothman's Episheet version 15 November 2021 wherever possible. For PM studies, model performance estimates in terms of discrimination (c statistic with accompanying 95% CI) and calibration (intercept and slope) were extracted. If calibration measures were absent, the total observed over expected (O/E) ratio with accompanying 95% CI of patients with the outcome was calculated wherever possible as a proxy of overall model calibration.(15) To this end, we used data on the distribution of subjects and outcomes across different PM risk strata and the corresponding predicted risks of the strata as reported in the original model development study. These calculations were performed in R version 4.2.2 using the 'metamisc' package.

Other data extraction items included study population, candidate predictors, sample size, (handling of) missing data, analysis (modelling method and/or model development and validation method), and interpretation of results. Where needed, corresponding authors of individual studies were contacted to request additional data.

Assessment of risk of bias and applicability

Risk of bias assessment was performed by two authors (MHR, TNP) independently using the Quality in Prognostic Studies tool (QUIPS, assessing study participation, study attrition, PF measurement, outcome measurement, and statistical analysis) and the Prediction Model Risk Of Bias Assessment Tool (PROBAST, covering the domains of participants, predictors, outcome, and analysis) for PF and PM studies, respectively.(17,18) We excluded the 'study confounding' item from the QUIPS, since confounding is not applicable to prognostic research. Applicability of included articles was assessed based on the PICOTS criteria.

Synthesis methods

For peer review only - http://bmjopen.bmj.com/site/about/guidelines.xhtml

Due to substantial heterogeneity in domain, predictor, and outcome definition, combining PF effect estimates or PM performance measures in meta-analyses was considered inappropriate and results were therefore descriptively summarised, stratified according to diagnosis (i.e. LRTI versus pneumonia). Forest plots were created to visually summarise (i) univariable and multivariable effect estimates of PFs, and (ii) discrimination and calibration performance measures of PMs. PFs were categorised into either demographics, patient history, healthcare use, medication use, signs and symptoms, laboratory tests, or diagnosis. Promising candidate predictors were identified based on reported results of multivariable analysis in PF studies. For each promising PF, we applied the grading of recommendations, assessment, development, and evaluations (GRADE) framework using prognostic research-specific guidance to rate the quality of evidence.(19)

Patient and public involvement

There was no patient or public involvement in this study.

For peer review only

Results

Study selection

The search strategy yielded a total of 3,191 records. Removing duplicates left 2,799 unique records (Figure 1). After screening title and abstract, 34 studies remained for full-text review. Of these, 19 were excluded (Figure 1). One additional publication was retrieved from screening reference lists(20), leaving 16 publications for inclusion: nine PF studies(20–28), six PM studies(29–34), and one combination of PM and PF(35).

Study characteristics

The PF studies were conducted between 1997-2021 of which most (7/10) used a retrospective cohort design (Table 1). The study population in terms of age (minimum age ranging from 1-80 years) and LRTI definition (ranging from suspected LRTI to diagnosis of CAP), as well as the hospitalisation rate (from 0.7% in LRTI patients to 76.5% CAP patients) varied substantially across studies. Mortality was used as outcome in four studies, hospitalisation in three studies, and a composite of both in one study. Two studies also included deterioration of existing illness and late-onset pneumonia in addition to hospitalisation and mortality in their outcome(22,35), while the timing of outcome assessment was unknown in one study(28).

The PM studies were conducted between 2000-2013 and the majority (4/7) used a prospective cohort design. In total, nine different PMs could be included in the synthesis: CRB, CRB-65, CURB, CURB-65, PSI, a model developed by Bont et al., RISSC85, CORB-75, and a model developed by Moore et al. (Table 2). Four studies developed a new prediction model(29,31,34,35) and five externally validated previously developed models(30–34), of which the CRB-65 was validated in all five studies. Clinical heterogeneity was mostly attributed to age (minimum age ranging from 16-65 years) and LRTI definition (ranging from suspected LRTI to radiographically confirmed CAP). Hospitalisation rates varied between 0.5% in LRTI patients and 86.9% in CAP patients. All PM studies used binary outcomes. Mortality was used as outcome in three studies, hospitalisation in one study, and a composite of both in one study.

For peer review only - http://bmjopen.bmj.com/site/about/guidelines.xhtml

One study also included re-consultation with new or worsened symptoms in addition to hospitalisation in their outcome(34), while another study included late-onset pneumonia in addition to hospitalisation and mortality.(35)

Risk of bias assessment

Overall risk of bias was judged high for all studies (Figures S2 and S3). For PF studies, this was mostly due to issues in the statistical analysis domains. For instance, five studies selected predictors for multivariable analysis based on univariable associations(20–24), and two included multiple episodes per patient but did not perform recurrent event analysis.(24,26) Furthermore, in some studies an adequate definition of LRTI or PFs was lacking, and methods to account for missing data were mostly judged inadequate or not described.

Similarly, risk of bias due to the issues in the analysis domain was judged high for all PM studies. In all model development studies continuous predictors were dichotomised, and handling of missing data was judged inadequate or not reported. None of the PM studies reported any evaluation of model calibration.

Prognostic factors

Three PF studies did not report estimates of univariable associations of PFs, and these could also not be calculated from the reported data.(23,26,27) The remaining PF studies reported univariable analysis of 69 PFs, of which sex, age, and history of pulmonary disease were analysed most frequently (Figure S4).

Estimates of multivariable associations were reported for 74 PFs (Figure S5), of which 21 were analysed in at least two studies. The number of covariates per multivariable analysis ranged from 3 to 29, and a common set of covariates across studies was lacking. Age was analysed in five studies, and all found increasing age to be associated with poor

For peer review only - http://bmjopen.bmj.com/site/about/guidelines.xhtml

outcome.(22,23,26,27,35) Apart from increasing age, PFs that were identified as promising based on multiple multivariable analyses were sex (significant association in two/five studies), current smoking (three/four studies), diabetes (two/three studies), a history of stroke (two/three studies), cancer (three/three studies), or heart failure (two/two studies), hospitalisation in the previous year (two/two studies), current use of systemic corticosteroids (two/three studies), antibiotic use in the previous month (two/three studies), a respiratory rate ≥25/minute (two/two studies), and diagnosis of pneumonia (two/two studies) (Figure 2). Having received an influenza vaccination in the previous year was the only promising candidate predictor estimated to have a negative association, based on a significant multivariable association in two/three studies. The quality of evidence was judged low for age, hospitalisation in the previous year, and diagnosis of pneumonia and very low for the remaining promising PFs (Table S6).

Prediction models

Several PMs included predictors that were also identified as promising candidate predictors based on PF studies (Table S7). Sex, current smoking, and influenza vaccination were the only promising candidate predictors that were not incorporated in any of the PMs. Predictors that were not identified as promising PFs but were incorporated in PMs include history of lung, renal or liver disease, extent to which complaints interfere with daily activities, sputum or coryza, chest pain, confusion, presence of crackles, heart rate, blood pressure, oxygen saturation, body temperature, blood urea nitrogen, and an overall GP severity assessment score.

Four studies developed a new PM (three for use in LRTI and one for CAP patients), in which the number of events per predictor ranged from 3.3-13.7.(29,33–35) C statistics ranged from 0.63 (95% CI 0.61-0.67) to 0.82 (95% CI 0.75-0.88) after (internal or external) model validation (Figure 3). One study performed external validation(29), whereas other studies only

For peer review only - http://bmjopen.bmj.com/site/about/guidelines.xhtml

conducted internal(-external) validation.(33–35) None reported measures of model calibration, while a total O/E ratio could not be calculated for any model.

All five PM validation studies externally validated the CRB-65.(30–34) C statistics for 30-day mortality in CAP patients (range outcome events: 11 to 80) ranged from 0.72 (95% CI 0.66-0.78) to 0.79 (95% CI 0.65-0.92)(30,31,33) (Figure 3). The c statistic for 28-day re-consultation or hospitalisation in LRTI patients was substantially lower: 0.53 (95% CI 0.51-0.56).(34) None reported measures of model calibration. A total O/E ratio could be calculated for the CRB-65 in four studies, ranging from 0.67 (95% CI 0.26-1.08) to 1.42 (95% CI 1.13-1.71).

Other validated PMs were PSI, CURB-65, CURB, and CRB. C statistics for 28-day re-consultation or hospitalisation in LRTI patients ranged from 0.51 (95% CI 0.50-0.54) to 0.53 (95% CI 0.50-0.55).(34) Calibration measures were not reported and total O/E ratios could not be calculated. C statistics of PSI and CURB-65 for 30-day mortality in CAP patients were 0.73 (95% CI 0.67-0.79) and 0.67 (95% CI 0.61-0.74), respectively.(31) Model calibration measures were not reported, while the total O/E ratios were estimated at 1.46 (95% CI 1.16-1.76) and 1.92 (95% CI 1.53-2.32), respectively.

For peer review only

For peer review only - http://bmjopen.bmj.com/site/about/guidelines.xhtml

Discussion

Summary of main findings

This systematic review summarised evidence on PFs and PMs for hospitalisation and mortality within 90 days in patients presenting to primary care with LRTI. The risk of bias of included studies was judged high, mainly due to issues in the analysis domains. Of all PFs analysed, thirteen were identified as most promising; increasing age, sex, current smoking, diabetes, a history of stroke, cancer, or heart failure, hospitalisation in the previous year, current use of systemic corticosteroids, antibiotic use in the previous month, a respiratory rate ≥25/minute, and diagnosis of pneumonia were associated with poor outcome whereas seasonal influenza vaccination was estimated to have a negative association. Most of these promising candidate predictors have also been incorporated in the identified PMs. The secondary care-derived CRB-65, predicting mortality in CAP patients, and a newly developed PM by Bont et al.(29) predicting hospitalisation and mortality in elderly LRTI patients both showed promising results after external validation. However, none assessed model calibration, leaving the precision of predicted risks unknown.

Comparison with existing literature

During the coronavirus disease 2019 (COVID-19) pandemic, many studies have focused on PFs associated with poor outcomes in COVID-19 patients, and some were similar to those identified in our review of all-cause LRTI patients. A recent, field-wide systematic review and meta-analysis of PFs for adverse outcomes in COVID-19 patients found cancer, insulin use, and smoking – among other factors – to be associated with mortality, and age with both hospitalisation and mortality.(36) Another community-based systematic review of European COVID-19 patients found male sex, heart failure, and diabetes to be associated with hospitalisation, whereas stroke, heart failure, and neurologic disease were associated with increased mortality risk.(37) Although we did not identify studies that assessed the prognostic

For peer review only - http://bmjopen.bmj.com/site/about/guidelines.xhtml

value of specific viral respiratory pathogens such as SARS-CoV-2, respiratory syncytial virus and influenza, it is likely that the specific pathogen is a relevant prognostic factor, and point-of-care tests for respiratory pathogens are increasingly available in the post-pandemic era. The potential usefulness of such point-of-care tests is supported by evidence of patients with a viral LRTI admitted to the emergency room in whom specific viral pathogens were associated with increased risk of hospitalisation.(38)

Some of the identified PMs have been incorporated in LRTI guidelines – mainly those focusing on CAP – as additional tool to aid patient management decisions. The CAP guideline issued by the American Thoracic Society (ATS) and the Infectious Diseases Society of America (IDSA) supports the use of PSI or CURB-65 – in addition to clinical judgement – to determine the appropriate level of care, i.e. inpatient versus outpatient management.(39,40) Although this guideline applies to all levels of health care, the value of the suggested models for GPs is limited since models' laboratory and radiographic features are not routinely available in primary care. The UK National Institute for Health and Care Excellence (NICE) guideline on pneumonia advocates the use of CRB-65; it recommends GPs to consider hospital admission in patients with a score of ≥1.(41) Guidelines vary with regard to the incorporation of individual factors that are associated with poor outcome. The ATS/IDSA guideline and the Dutch primary care guideline both list tachypnoea and confusion as indicators of disease severity, while the Dutch guideline also emphasised the importance of PFs such as age, history of heart failure, and diabetes.(39,42) The NICE guideline does not explicitly highlight individual factors associated with poor outcome in adult patients.(41)

Similar to our findings, a review of the performance of CRB-65 for predicting 30-day mortality among CAP patients in community and hospital settings concluded that this model has been insufficiently validated for use in primary care.(43) Although the population of this review slightly differed from ours by also including patients who visited the emergency department, a similarly low number of outcome events was found. Contrary to the slight overprediction reported in this review, our synthesis of more recent studies suggested

For peer review only

underprediction of risks based on total O/E ratios. However, in absence of proper assessment of model calibration the true precision of predicted risks remains to be elucidated.

Strengths and limitations

By covering both PFs and PMs in a single review, we have provided a comprehensive overview of available prognostic literature of primary care LRTI patients. We prospectively registered our review protocol, conducted a broad search with incorporation of a validated prognostic search string, and thoroughly reviewed citations of eligible studies to assure all relevant literature was included in the synthesis. By including various prognostic study designs, our synthesis provides a comprehensive overview of all prognostic evidence on primary care LRTI patients. Studies assessing the prognostic value of individual factors, developing a new or validating an existing model all provide insight on important PFs that could be used in future prognostic research, whereas studies validating a new or previously developed model such as CRB-65 provide insight on the suitability for its use in clinical practice.

Some limitations should, however, be acknowledged. First, our findings should be interpreted with caution since risk of bias was judged high for all included studies and the quality of the evidence was judged low to very low for the various promising PFs. Second, due to substantial heterogeneity in domain, predictor, and outcome definitions between studies, we refrained from conducting meta-analyses of PF effect estimates or PM performance measures, which makes direct comparison of study results difficult. It is plausible that PF effect estimates for poor outcome vary according to age, diagnosis, and disease severity (a priori risk of event). For example, half of the participants in Hak et al.(22) had either pneumonia or an exacerbation of chronic obstructive pulmonary disease, whereas Moore et al.(35) had fewer than 2% of such patients. Furthermore, one study included patients aged >1 year (median age of included participants: 54 years [interquartile range: 37]) but did not specifically report separate results for age strata.(23) Given the median age, we considered including this study on prognostic

For peer review only - http://bmjopen.bmj.com/site/about/guidelines.xhtml

factors in our review justified. Consistency in effect estimates would have further facilitated direct comparison of PFs, but converting the various reported estimates to odds ratios appeared not possible. Albeit our synthesis of PFs provides some useful information for clinicians, it merely informs future research aimed at developing or updating PMs. Third, we excluded most studies on patients with a specific respiratory pathogen (e.g. SARS-CoV-2 or influenza) since these included patients with both upper and lower respiratory tract infections and did not report stratified results for LRTI. As such, we were unable to present any respiratory pathogen-specific results regarding prognostic factors for poor outcome in patients presenting to primary care with LRTI. However, since GPs are typically unaware of the specific pathogen in LRTI patients, such results would have limited applicability to current clinical practice. Lastly, PM building techniques were generally suboptimal which increased risk of bias, and none of the identified PM studies assessed model calibration. In contrast to discrimination, calibration generally receives little attention when assessing PM performance, whereas well discriminating but poorly calibrated models can lead to imprecise, misleading predictions.(44) Total O/E ratios calculations provided some insight into mean model calibration – the most basic level in the hierarchy of calibration assessment – but this is insufficient to provide guidance on the safe use of a PM in clinical practice.(45)

Implications for research and clinical practice

Based on our synthesis, implementation of existing PMs for individualised risk prediction of 90-day hospitalisation or all-cause mortality in primary care LRTI patients in everyday practice is hampered by incomplete assessment of model performance. The identified candidate predictors provide useful information for clinicians about factors associated with poor outcome in primary care LRTI patients and warrant consideration when developing or updating PMs using state-of-the-art development and appropriate validation techniques.

For peer review only

Declarations

Author contributions

MHR, TNP, MvS, and RPV were involved in the conceptualisation and design of the study. MHR, TMCB, and TNP conducted the study selection procedure. Data of eligible studies was extracted by MHR. MHR, TNP, and MvS performed the risk of bias assessment. MHR wrote the first draft of the manuscript, which was initially reviewed by TNP, MvS, and RVP. MHR, TNP, TMCB, GJG, PL, FHR, MvS, and RVP reviewed and approved the final manuscript.

Ethical approval

This study did not involve human participants and ethical approval was therefore not required.

Funding

This study was funded by ZonMw (grant number 08391052110003). The funder played no role in study design, data collection, analysis and interpretation of data, or the writing of this manuscript.

Competing interests

All authors declare no financial or non-financial competing interests.

Patient and public involvement

There was no patient or public involvement in this study.

Data availability

For peer review only

Study materials (e.g. template data collection forms, data extracted from included studies) are available from the corresponding author upon reasonable request.

For peer review only

For peer review only - http://bmjopen.bmj.com/site/about/guidelines.xhtml

References

1. Bergmann M, Haasenritter J, Beidatsch D, Schwarm S, Hörner K, Bösner S, et al. Prevalence, aetiologies and prognosis of the symptom cough in primary care: a systematic review and meta-analysis. BMC Fam Pract. 2021;22(1):1–19.

2. Hak E, Rovers MM, Kuyvenhoven MM, Schellevis FG, Verheij TJM. Incidence of GP-diagnosed respiratory tract infections according to age, gender and high-risk co-morbidity: The Second Dutch National Survey of General Practice. Fam Pract. 2006;23(3):291–4.

3. Little P, Stuart B, Moore M, Coenen S, Butler CC, Godycki-Cwirko M, et al. Amoxicillin for acute lower-respiratory-tract infection in primary care when pneumonia is not suspected: A 12-country, randomised, placebo-controlled trial. Lancet Infect Dis. 2013;13(2):123–9.

4. Moore M, Stuart B, Coenen S, Butler CC, Goossens H, Verheij TJM, et al. Amoxicillin for acute lower respiratory tract infection in primary care: Subgroup analysis of potential high-risk groups. British Journal of General Practice. 2014;64(619):75–80.

5. Little P, Stuart B, Smith S, Thompson MJ, Knox K, Van Den Bruel A, et al. Antibiotic prescription strategies and adverse outcome for uncomplicated lower respiratory tract infections: Prospective cough complication cohort (3C) study. BMJ (Online). 2017;357.

6. Welte T, Torres A, Nathwani D. Clinical and economic burden of community-acquired pneumonia among adults in Europe. Thorax. 2012;67(1):71–9.

7. McLaughlin JM, Khan FL, Thoburn EA, Isturiz RE, Swerdlow DL. Rates of hospitalization for community-acquired pneumonia among US adults: A systematic review. Vaccine. 2020;38(4):741–51.

8. Teepe J, Broekhuizen BDL, Loens K, Lammens C, Ieven M, Goossens H, et al. Predicting the presence of bacterial pathogens in the airways of primary care patients with acute cough. CMAJ. 2017;189(2):E50–5.

9. Minnaard MC, De Groot JAH, Hopstaken RM, Schierenberg A, De Wit NJ, Reitsma JB, et al. The added value of C-reactive protein measurement in diagnosing pneumonia in primary care: A meta-analysis of individual patient data. CMAJ. 2017;189(2):E56–63.

10. Lim WS, Van Der Eerden MM, Laing R, Boersma WG, Karalus N, Town GI, et al. Defining community acquired pneumonia severity on presentation to hospital: An international derivation and validation study. Thorax. 2003;58(5):377–82.

11. Fine M, Auble T, Yealy D, Hanusa B, Weissfeld L, Singer D, et al. A prediction rule to identify low-risk patients with community-acquired pneumonia. New England Journal of Medicine. 1997;336(4):243–50.

12. Page MJ, McKenzie JE, Bossuyt PM, Boutron I, Hoffmann TC, Mulrow CD, et al. The PRISMA 2020 statement: An updated guideline for reporting systematic reviews. The BMJ. 2021;372.

13. Geersing GJ, Bouwmeester W, Zuithoff P, Spijker R, Leeflang M, Moons K. Search filters for finding prognostic and diagnostic prediction studies in medline to enhance systematic reviews. PLoS One. 2012;7(2):3–8.

For peer review only - http://bmjopen.bmj.com/site/about/guidelines.xhtml

14. Moons KGM, de Groot JAH, Bouwmeester W, Vergouwe Y, Mallett S, Altman DG, et al. Critical Appraisal and Data Extraction for Systematic Reviews of Prediction Modelling Studies: The CHARMS Checklist. PLoS Med. 2014;11(10).

15. Debray TPA, Damen JAAG, Snell KIE, Ensor J, Hooft L, Reitsma JB, et al. A guide to systematic review and meta-analysis of prediction model performance. BMJ (Online). 2017;356.

16. Riley RD, Moons KGM, Snell KIE, Ensor J, Hooft L, Altman DG, et al. A guide to systematic review and meta-analysis of prognostic factor studies. BMJ (Online). 2019;364.

17. Hayden JA, van der Windt DA, Cartwright JL, Côté P, Bombardier C. Assessing Bias in Studies of Prognostic Factors. Ann Intern Med. 2013;158:280–6.

18. Wolff RF, Moons KGM, Riley RD, Whiting PF, Westwood M, Collins GS, et al. PROBAST: A tool to assess the risk of bias and applicability of prediction model studies. Ann Intern Med. 2019;170(1):51–8.

19. Foroutan F, Guyatt G, Zuk V, Vandvik PO, Alba AC, Mustafa R, et al. GRADE Guidelines 28: Use of GRADE for the assessment of evidence about prognostic factors: rating certainty in identification of groups of patients with different absolute risks. J Clin Epidemiol. 2020 May 1;121:62–70.

20. Houston MS, Silverstein MD, Suman VJ. Risk Factors for 30-Day Mortality in Elderly Patients With Lower Respiratory Tract Infection: Community-Based Study. Arch Intern Med. 1997;157:2190–5.

21. Seppä Y, Bloigu A, Honkanen PO, Miettinen L, Syrjälä H. Severity assessment of lower respiratory tract infection in elderly patients in primary care. Arch Intern Med. 2001;161(22):2709–13.

22. Hak E, Bont J, Hoes AW, Verheij TJM. Prognostic factors for serious morbidity and mortality from community-acquired lower respiratory tract infections among the elderly in primary care. Fam Pract. 2005;22(4):375–80.

23. Winchester CC, Macfarlane T V., Thomas M, Price D. Antibiotic prescribing and outcomes of lower respiratory tract infection in UK primary care. Chest. 2009;135(5):1163–72.

24. van de Nadort C, Smeets HM, Bont J, Zuithoff NPA, Hak E, Verheij TJM. Prognosis of primary care patients aged 80 years and older with lower respiratory tract infection. British Journal of General Practice. 2009;59(561):261–5.

25. Myles P, Hubbard R, Gibson J, Pogson Z, Smith C, McKeever T. The impact of statins, ace inhibitors and gastric acid suppressants on pneumonia mortality in a UK general practice population cohort. Pharmacoepidemiol Drug Saf. 2009;18(May):697–703.

26. Millett ERC, de Stavola BL, Quint JK, Smeeth L, Thomas SL. Risk factors for hospital admission in the 28 days following a community-acquired pneumonia diagnosis in older adults, and their contribution to increasing hospitalisation rates over time: A cohort study. BMJ Open. 2015;5(12).

27. Hamilton F, Arnold D, Payne R. Association of prior lymphopenia with mortality in pneumonia: A cohort study in UK primary care. British Journal of General Practice. 2021;71(703):E148–56.

For peer review only - http://bmjopen.bmj.com/site/about/guidelines.xhtml

28. Martínez-Redondo J, Comas C, Salud JP, Crespo-Pons M, García-Serrano C, Bravo MO, et al. The risk of hospitalization in COVID-19 patients can be predicted by lung ultrasound in primary care. Int J Environ Res Public Health. 2021;18(11).

29. Bont J, Hak E, Hoes AW, Schipper M, Schellevis FG, Verheij TJM. A prediction rule for elderly primary-care patients with lower respiratory tract infections. European Respiratory Journal. 2007;29(5):969–75.

30. Bont J, Hak E, Hoes AW, Macfarlane JT, Verheij TJ. Predicting death in elderly patients with community-acquired pneumonia: a prospective validation study reevaluating the CRB-65 severity assessment tool. Arch Intern Med. 2008;168(13):1465–8.

31. Ochoa-Gondar O, Vila-Corcoles A, Rodriguez-Blanco T, Ramos F, De Diego C, Salsench E, et al. Comparison of three predictive rules for assessing severity in elderly patients with CAP. Int J Clin Pract. 2011;65(11):1165–72.

32. Francis NA, Cals JW, Butler CC, Hood K, Verheij T, Little P, et al. Severity assessment for lower respiratory tract infections: Potential use and validity of the CRB-65 in primary care. Primary Care Respiratory Journal. 2012;21(1):65–70.

33. Ochoa-Gondar O, Vila-Corcoles A, Rodriguez-Blanco T, Hospital I, Salsench E, Ansa X, et al. Validation of the CORB75 (confusion, oxygen saturation, respiratory rate, blood pressure, and age ≥75 years) as a simpler pneumonia severity rule. Infection. 2013;42(2):371–8.

34. Bruyndonckx R, Hens N, Verheij TJM, Aerts M, Ieven M, Butler CC, et al. Development of a prediction tool for patients presenting with acute cough in primary care: A prognostic study spanning six European countries. British Journal of General Practice. 2018;68(670):e342–50.

35. Moore M, Stuart B, Lown M, Van Den Bruel A, Smith S, Knox K, et al. Predictors of adverse outcomes in uncomplicated lower respiratory tract infections. Ann Fam Med. 2019;17(3):231–8.

36. Bellou V, Tzoulaki I, van Smeden M, Moons KGM, Evangelou E, Belbasis L. Prognostic factors for adverse outcomes in patients with COVID-19: A field-wide systematic review and meta-analysis. Vol. 59, European Respiratory Journal. European Respiratory Society; 2022.

37. Vardavas CI, Mathioudakis AG, Nikitara K, Stamatelopoulos K, Georgiopoulos G, Phalkey R, et al. Prognostic factors for mortality, intensive care unit and hospital admission due to SARS-CoV-2: a systematic review and metaanalysis of cohort studies in Europe. Vol. 31, European Respiratory Review. European Respiratory Society; 2022.

38. Lemarie B, Boussaid G, Gault E, Prigent H, Beaune S, Moreau F, et al. Predictors of Hospitalization and Superinfection in Viral Respiratory Tract Infections Between Influenza and Paramyxoviruses: The SUPERFLUOUS Study. Journal of Infectious Diseases. 2022 Sep 15;226(6):1027–35.

39. Metlay JP, Waterer GW, Long AC, Anzueto A, Brozek J, Crothers K, et al. Diagnosis and treatment of adults with community-acquired pneumonia. Am J Respir Crit Care Med. 2019 Oct 1;200(7):E45–67.

40. Armstrong CAFP. Community-Acquired Pneumonia: Updated Recommendations from the ATS and IDSA. Am Fam Physician. 2020;102(2):121–4.

For peer review only - http://bmjopen.bmj.com/site/about/guidelines.xhtml

41. National Institute for Health and Care Excellence (NICE). Pneumonia in adults: diagnosis and management (Clinical guideline 191) [Internet]. 2014. Available from: www.nice.org.uk/guidance/cg191

42. Verheij T, Hopstaken R, Prins J, Salomé P, Bindels P, Ponsioen B, et al. Nederlands Huisartsen Genootschap. 2020 [cited 2022 May 25]. NHG-Standaard Acuut hoesten. Available from: https://richtlijnen.nhg.org/standaarden/acuut-hoesten#volledige-tekst

43. McNally M, Curtain J, O'Brien KK, Dimitrov BD, Fahey T. Validity of British Thoracic Society guidance (the CRB-65 rule) for predicting the severity of pneumonia in general practice: Systematic review and meta-analysis. British Journal of General Practice. 2010;60(579):423–33.

44. van Calster B, McLernon DJ, van Smeden M, Wynants L, Steyerberg EW, Bossuyt P, et al. Calibration: The Achilles heel of predictive analytics. BMC Med. 2019 Dec 16;17(1).

45. van Calster B, Nieboer D, Vergouwe Y, de Cock B, Pencina MJ, Steyerberg EW. A calibration hierarchy for risk models was defined: From utopia to empirical data. J Clin Epidemiol. 2016 Jun 1;74:167–76.

For peer review only

Tables

Table 1. Study characteristics of included prognostic factor studies

Study	*Source of data*	*Study population*	*Included episodes*	*Age, mean (SD) or median (IQR)*	*Outcome* [a]	*Hospitalisation, n (%)*	*Mortality, n (%)*	*Total outcome events, n (%)*
**Houston, 1997**	Community-based cohort, retrospective	Elderly (≥65 years) LRTI patients	413	NR	30-day all-cause mortality	NR	44 (10.7)	44 (10.7)
**Seppä, 2001**	Primary care cohort, prospective	Elderly (≥65 years) patients with LRTI suggestive of pneumonia	950	Survival: 76 (IQR 71-81), death: 80 (IQR 73-86)	LRTI-related mortality within 30 days	NR	38 (4.0)	38 (4.0)
**Hak, 2005**	Primary care cohort, retrospective	Patients ≥60 years with an LRTI	455	75 (SD 8.6)	Composite of: i) Dysregulation of diabetes, stroke, heart failure, MI, ii) hospitalisation, iii) all-cause mortality, all within 30 days	21 (4.6)	24 (5.3)	65 (14.3)
**Winchester, 2009**	Primary care cohort, retrospective	Patients ≥1 year with an LRTI	151,088	54 (IQR 37)	Respiratory infection-related admission or mortality within 3 months	1,147 (0.8)	2,126 (1.4)	NR
**Van de Nadort, 2009**	Primary care cohort, retrospective	Elderly (≥80 years) LRTI patients	860	85.2 (no SD)	Hospitalisation or death within 30 days	NR	51 (5.9)	109 (12.7)
**Myles, 2009**	Primary care cohort, retrospective	Patients ≥40 years with an LRTI	3,681	NR	All-cause mortality within 30 days	NR	905 (24.6)	905 (24.6)
**Millett, 2015**	Primary care cohort, retrospective	Elderly (≥65 years) CAP patients	43,576	81 (IQR 75-87)	Hospital admission within 28 days	33,321 (76.5)	NR	33,321 (76.5)

For peer review only - http://bmjopen.bmj.com/site/about/guidelines.xhtml

**Moore, 2019**	Primary care cohort, prospective	Patients ≥16 years with acute cough attributed to LRTI	28,846	NR	LRTI-related hospitalisation, mortality or late-onset pneumonia within 2-30 days	196 (0.7)	29 (0.1)	325 (1.1)
**Hamilton, 2021**	Primary care cohort, retrospective	Patients ≥50 years with pneumonia and history of lymphocyte test	28,556	76 (IQR 66-85)	28-day mortality	NR	2,544 (8.9)	2,544 (8.9)
**Martínez-Redondo, 2021**	Primary care cohort, prospective	Adults (≥18 years) with a COVID-19 pneumonia	161	53.0 (no SD)	Hospital admission, no time window reported	37 (23.0)	NR	37 (23.0)

Abbreviations: LRTI, lower respiratory tract infection; CAP, community-acquired pneumonia; COVID-19, coronavirus disease 2019; SD, standard deviation; IQR, interquartile range; NR, not reported; MI, myocardial infarction.

[a] Time window of outcome is defined as days from first consultation within LRTI episode.

For peer review only

For peer review only - http://bmjopen.bmj.com/site/about/guidelines.xhtml

Table 2. Study characteristics of included prediction model studies

Study	*Type of study*	*Model(s)*	*Source of data*	*Study population*	*Age, mean (SD)*	*Outcome [a]*	*Total outcome events, n (%)*	*Method of validation*
**Bont, 2007**	D+V	New	Primary care cohort, retrospective	Elderly (≥65) LRTI patients	75.5 (no SD)	Hospitalisation or all-cause mortality within 30 days	D: 274 (8.7), V: 178 (7.2)	Internal (random split-sample) and external
**Bont, 2008**	V	CRB-65	Primary care cohort, prospective	Elderly (≥65) CAP patients	77.3 (no SD)	Mortality within 30 days	11 (3.5)	External
**Ochoa-Gondar, 2011**	V	PSI, CURB-65, CRB-65	Population-based cohort, retrospective	Elderly (≥65) patients with radiographically confirmed CAP	77.4 (7.6)	Mortality within 30 days	80 (13.6)	External
**Francis, 2012**	V	CRB-65	Primary care cohort, prospective	Adults (≥18) presenting with acute cough or suspected LRTI	49.3 (16.5)	Hospitalisation within 28 days	10 (2.9)	External
**Ochoa-Gondar, 2013**	D+V	CORB-75 (new), CRB-65	Population-based cohort, retrospective	Elderly (≥65) patients with radiographically confirmed CAP	78.1 (7.7)	Mortality within 30 days	D: 46 (13.1), V: 34 (13.1)	Internal-external (temporal, CORB-75), external (CRB-65)
**Bruyndonckx, 2018**	D (RISCC85) + V (all models)	RISCC85 (new), PSI (stage I), CRB, CRB-65, CURB, CURB-65	Primary care cohort, prospective	Adults (≥18) with symptoms suggestive of LRTI	50 (17)	Re-consultation with new/worsened symptoms or hospitalisation within 28 days	521 (20.0)	Internal (random split sample, RISCC85), external (other models)
**Moore, 2019**	D+V	New	Primary care cohort, prospective	Patients ≥16 years with acute cough attributed to LRTI	NR	Hospitalisation, mortality, or clinical diagnosis of pneumonia within 2-30 days	325 (1.1)	Internal (bootstrapping)

For peer review only - http://bmjopen.bmj.com/site/about/guidelines.xhtml

[a] Time window of outcome is defined as days from first consultation.

Abbreviations: D, development; V, validation; LRTI, lower respiratory tract infection; CAP, community acquired pneumonia; SD, standard deviation; NR, not reported.

For peer review only

For peer review only - http://bmjopen.bmj.com/site/about/guidelines.xhtml

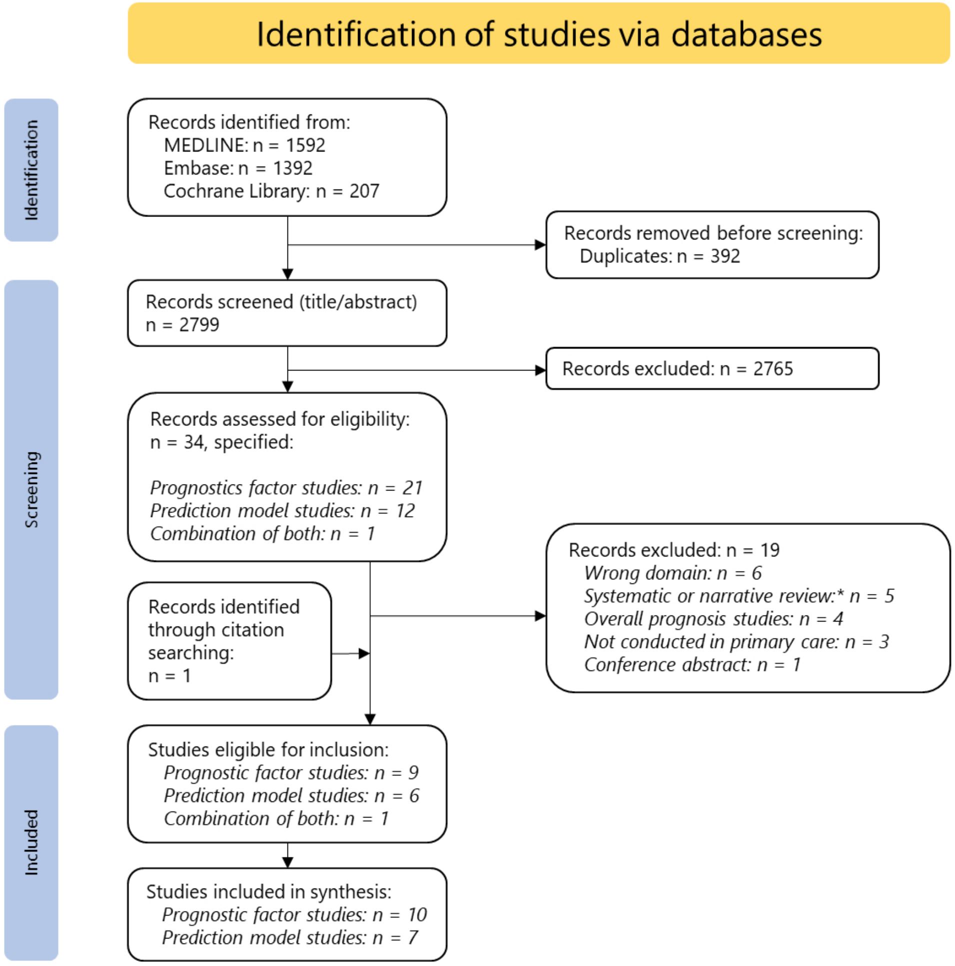

Figure 1. Flow diagram of search results.

Legend:
* Systematic reviews have been screened for eligible primary studies.

184x182mm (150 x 150 DPI)

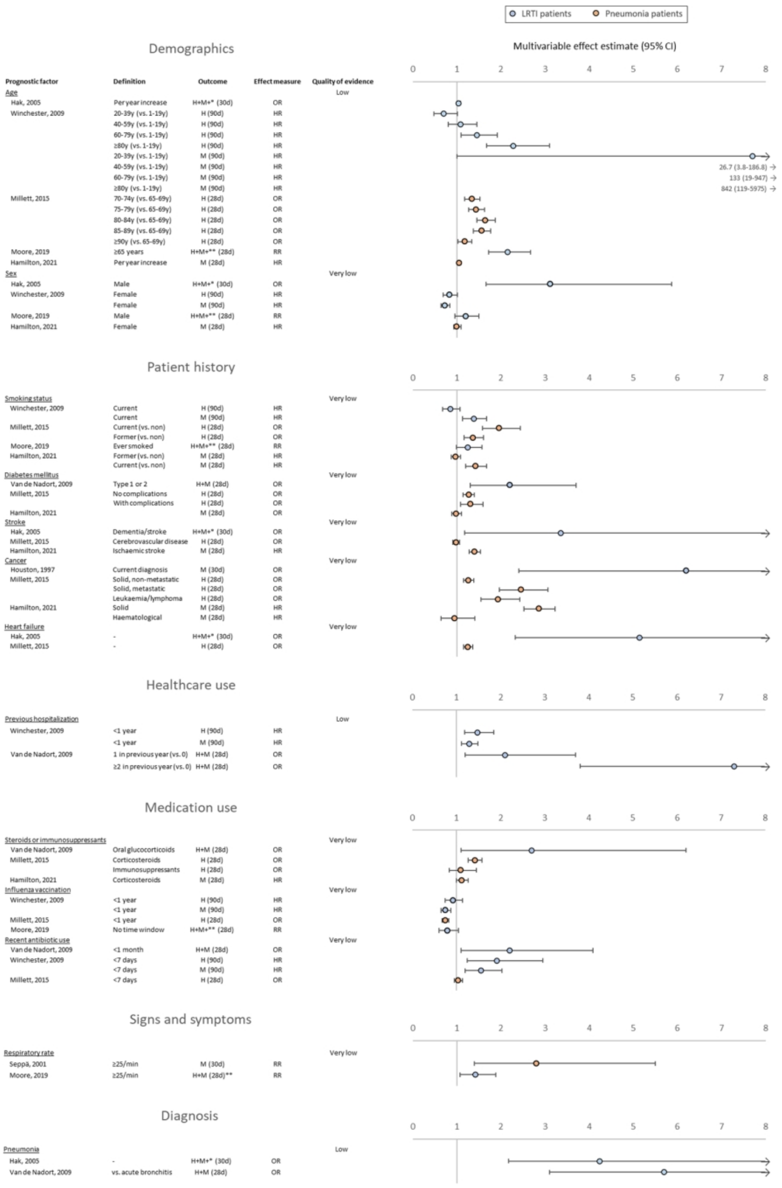

Figure 2. Overview of most promising candidate predictors for hospitalisation or mortality within 90 days based on multivariable analysis.

Legend:
- Blue coloured points: studies on LRTI patients
- Orange coloured points: studies on pneumonia patients

Quality of evidence: as judged based on the GRADE framework.
* Composite outcome also includes dysregulation of diabetes, stroke, heart failure, MI.
** Composite outcome also includes late onset pneumonia.
Abbreviations: LRTI, lower respiratory tract infection; CAP, community-acquired pneumonia; H, hospitalisation; M, mortality; OR, odds ratio; HR, hazard ratio; RR, risk ratio; min, minute.

133x196mm (300 x 300 DPI)

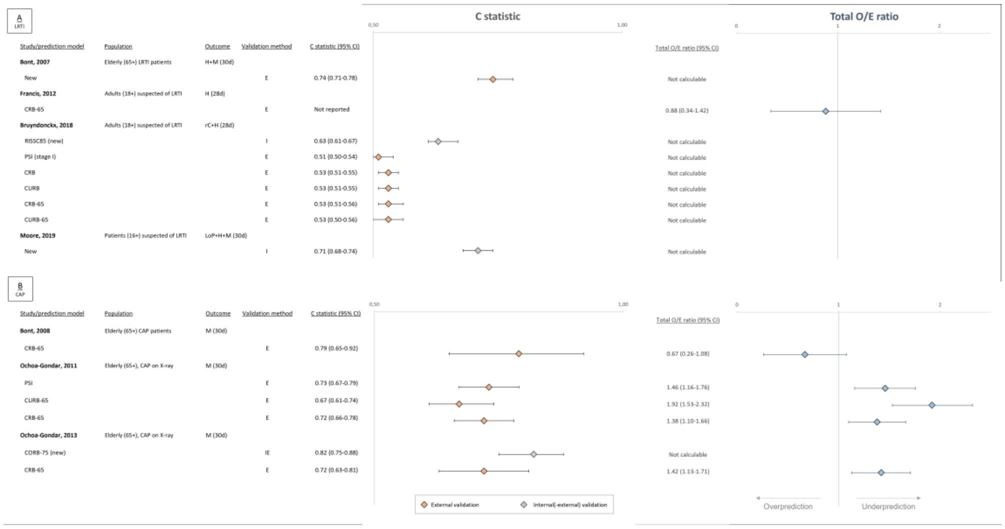

Figure 3. Overview of discrimination and calibration performance measures of the included prediction models

Legend:
A. Performance of prediction models evaluated in LRTI patients. B. Performance of prediction models evaluated in community-acquired pneumonia patients.
Abbreviations: LRTI, lower respiratory tract infection; CAP, community-acquired pneumonia; H, hospitalisation; M, mortality; rC, reconsultation; LoP, late onset pneumonia; E, external; IE, internal-external; I, internal; CI, confidence interval; O/E, observed over expected.

557x290mm (330 x 330 DPI)

Supplementary data

Table S1: search strategy

Search date: 27 June 2022		
**Pubmed**	**Embase**	**The Cochrane Library**
((((("RTI"[Title/Abstract] OR "respiratory tract infection*"[Title/Abstract] OR "respiratory infection*"[Title/Abstract] OR "pneumonia*"[Title/Abstract] OR "bronchitis"[Title/Abstract] OR "lung infection*"[Title/Abstract] OR "chest infection*"[Title/Abstract]) OR ("Respiratory Tract Infections"[Mesh])) AND (((Validat* OR Predict*[Title] OR Rule*) OR (Predict* AND (Outcome* OR Risk* OR Model*)) OR ((History OR Variable* OR Criteria OR Scor* OR Characteristic* OR Finding* OR Factor*) AND (Predict* OR Model* OR Decision* OR Identif* OR Prognos*)) OR (Decision* AND (Model* OR Clinical* OR Logistic Models)) OR (Prognostic AND (History OR Variable* OR Criteria OR Scor* OR Characteristic* OR Finding* OR Factor* OR Model*))) OR (“Stratification” OR “ROC Curve”[Mesh] OR “Discrimination” OR “Discriminate” OR “c-statistic” OR “c statistic” OR “Area under the curve” OR “AUC” OR “Calibration” OR “Indices” OR “Algorithm” OR “Multivariable”))) AND (("hospital*"[Title/Abstract] OR "complicat*"[Title/Abstract] OR "morbidity"[Title/Abstract] OR "death"[Title/Abstract] OR "mortality"[Title/Abstract] OR	(('rti':ti,ab,kw OR 'respiratory tract infection*':ti,ab,kw OR 'respiratory infection*':ti,ab,kw OR 'pneumonia*':ti,ab,kw OR 'bronchitis':ti,ab,kw OR 'lung infection*':ti,ab,kw OR 'chest infection*':ti,ab,kw) OR ('respiratory tract infection'/exp OR 'respiratory tract inflammation'/exp)) AND ((validat* OR predict*:ti OR rule* OR (predict* AND (outcome* OR risk* OR model*)) OR ((history OR variable* OR criteria OR scor* OR characteristic* OR finding* OR factor*) AND (predict* OR model* OR decision* OR identif* OR prognos*)) OR (decision* AND (model* OR clinical* OR logistic) AND models) OR (prognostic AND (history OR variable* OR criteria OR scor* OR characteristic* OR finding* OR factor* OR model*))) OR ('stratification' OR 'receiver operating characteristic'/exp OR 'discrimination' OR 'discriminate' OR 'c-statistic' OR 'c statistic' OR 'area under the curve' OR 'auc' OR 'calibration' OR 'indices' OR 'algorithm' OR 'multivariable')) AND (('hospital*':ti,ab,kw OR 'complicat*':ti,ab,kw OR 'morbidity':ti,ab,kw OR 'death':ti,ab,kw OR 'mortality':ti,ab,kw OR 'surviv*':ti,ab,kw) OR ('hospitalization'/exp OR 'hospital admission'/exp OR	(RTI OR respiratory tract infection OR respiratory infection OR pneumonia OR bronchitis OR lung infection OR chest infection) AND (hospitalisation OR hospitalization OR hospital OR complication OR complicated OR morbidity OR death OR mortality OR survival OR survive) AND (Primary care OR general practice OR general practitioner OR family practice OR GP) in Title Abstract Keyword

For peer review only

"surviv*"[Title/Abstract]) OR ("Mortality"[Mesh] OR "Hospitalization"[Mesh] OR "Death"[Mesh]))) AND (("Primary care"[Title/Abstract] OR "general practi*"[Title/Abstract] OR "family practice"[Title/Abstract] OR "GP"[Title/Abstract]) OR ("Primary Health Care"[Mesh] OR "Family Practice"[Mesh] OR "General Practice"[Mesh]))) NOT (("child*"[Title/Abstract] OR "pediatric*"[Title/Abstract] OR "paediatric*"[Title/Abstract] OR "infan*"[Title/Abstract] OR "adolescen*"[Title/Abstract] OR "young*"[Title/Abstract] OR “newborn*”[Title/Abstract]) OR ("Infant"[Mesh] OR "Child"[Mesh] OR "Adolescent"[Mesh] OR "Pediatrics"[Mesh] OR "Young Adult"[Mesh]))	'death'/exp OR 'mortality'/exp)) AND (('primary care':ti,ab,kw OR 'general practi*':ti,ab,kw OR 'family practice':ti,ab,kw OR 'gp':ti,ab,kw) OR ('primary medical care'/exp OR 'primary health care'/exp OR 'general practice'/exp)) NOT (('child*':ti,ab,kw OR 'pediatric*':ti,ab,kw OR 'paediatric*':ti,ab,kw OR 'infan*':ti,ab,kw OR 'adolescen*':ti,ab,kw OR 'young*':ti,ab,kw OR ‘newborn*’:ti,ab,kw) OR ('child'/exp OR 'adolescent'/exp OR 'pediatrics'/exp))	
Filters: none<br>Language restrictions: none<br>Publication date restrictions: none	Filters: none<br>Language restrictions: none<br>Publication date restrictions: none	Filters: none<br>Language restrictions: none<br>Publication date restrictions: none
Records identified: 1592	Records identified: 1392	Records identified: 207

For peer review only

Figure S2: Overall assessment of risk of bias and applicability of included studies

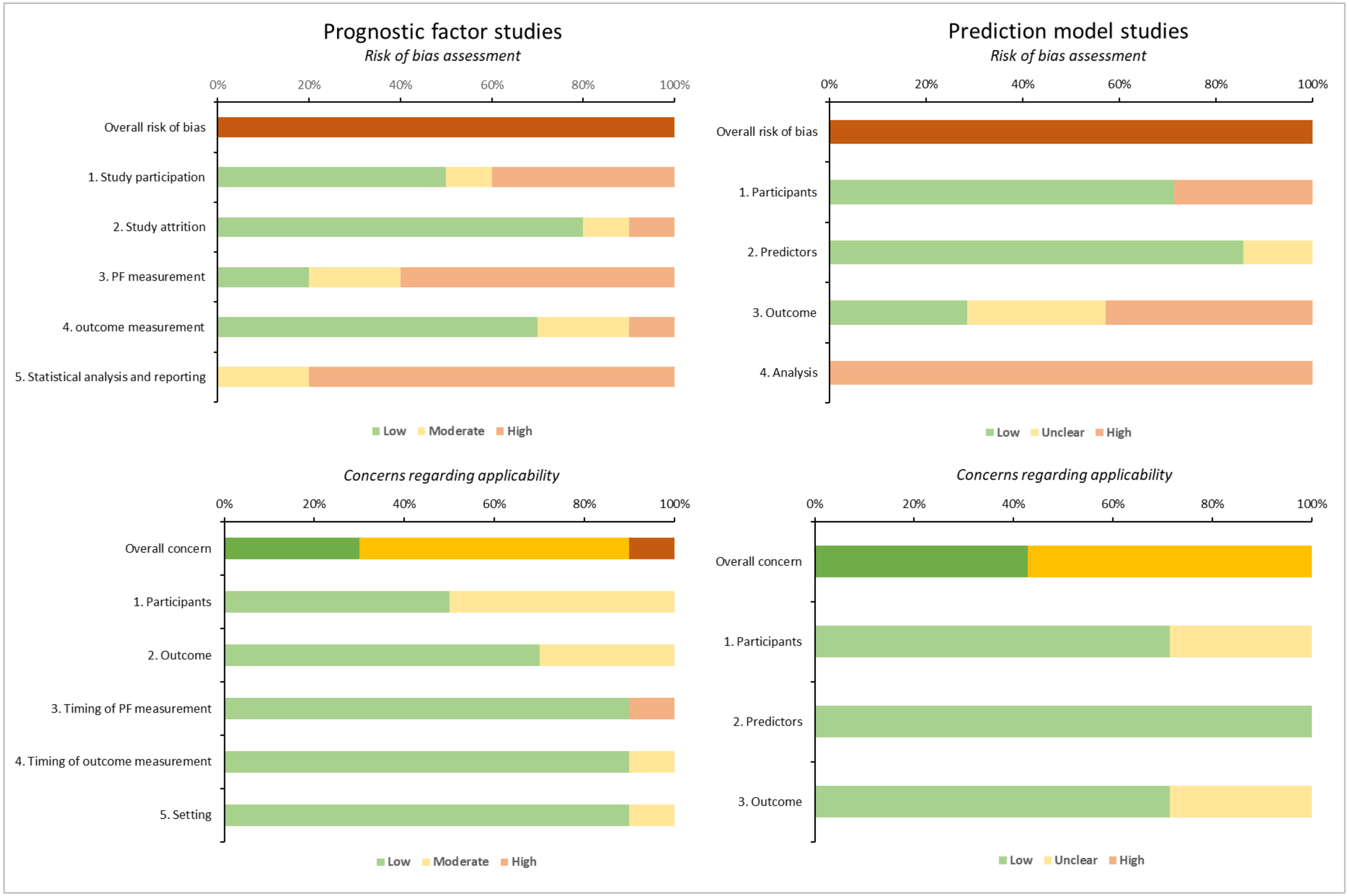

Figure S3: Risk of bias assessment and applicability per individual included study

QUIPS assessment of prognostic factor studies

Study	RoB					Applicability					Overall	
	Study participation	Study attrition	PF measurement	Outcome measurement	Statistical analysis and reporting	Participants	Outcome	Timing of PF measurement	Timing of outcome measurement	Setting	RoB	Applicability
Houston, 1997	±	+	-	+	-	+	+	+	+	±	-	±
Seppä, 2001	-	±	-	+	-	+	+	+	+	+	-	+
Hak, 2005	+	+	±	+	-	+	±	+	+	+	-	±
Myles, 2009	-	+	+	±	-	+	+	+	+	+	-	+
van de Nadort, 2009	+	+	+	±	-	±	+	+	+	+	-	±
Winchester, 2009	+	+	-	+	-	±	±	+	+	+	-	±
Millett, 2015	+	+	±	+	-	+	+	+	+	+	-	+
Moore, 2019	-	+	-	+	±	±	±	+	+	+	-	±
Hamilton, 2021	+	-	-	+	±	±	+	+	+	+	-	±
Martínez-Redondo, 2021	-	+	-	-	-	±	+	-	±	+	-	-

PROBAST assessment of prediction model studies

Study	RoB				Applicability			Overall	
	Participants	Predictors	Outcome	Analysis	Participants	Predictors	Outcome	RoB	Applicability
Bont, 2007	+	+	-	-	+	+	+	-	+
Bont, 2008	-	+	?	-	+	+	+	-	+
Ochoa-Gondar, 2011	+	?	?	-	?	+	+	-	?
Francis, 2012	-	+	-	-	+	+	+	-	+
Ochoa-Gondar, 2013	+	+	+	-	?	+	+	-	?
Bruyndonckx, 2018	+	+	+	-	+	+	?	-	?
Moore, 2019	+	+	+	-	+	+	?	-	?

Figure S4: figure of all PF effect estimates based on univariable analysis

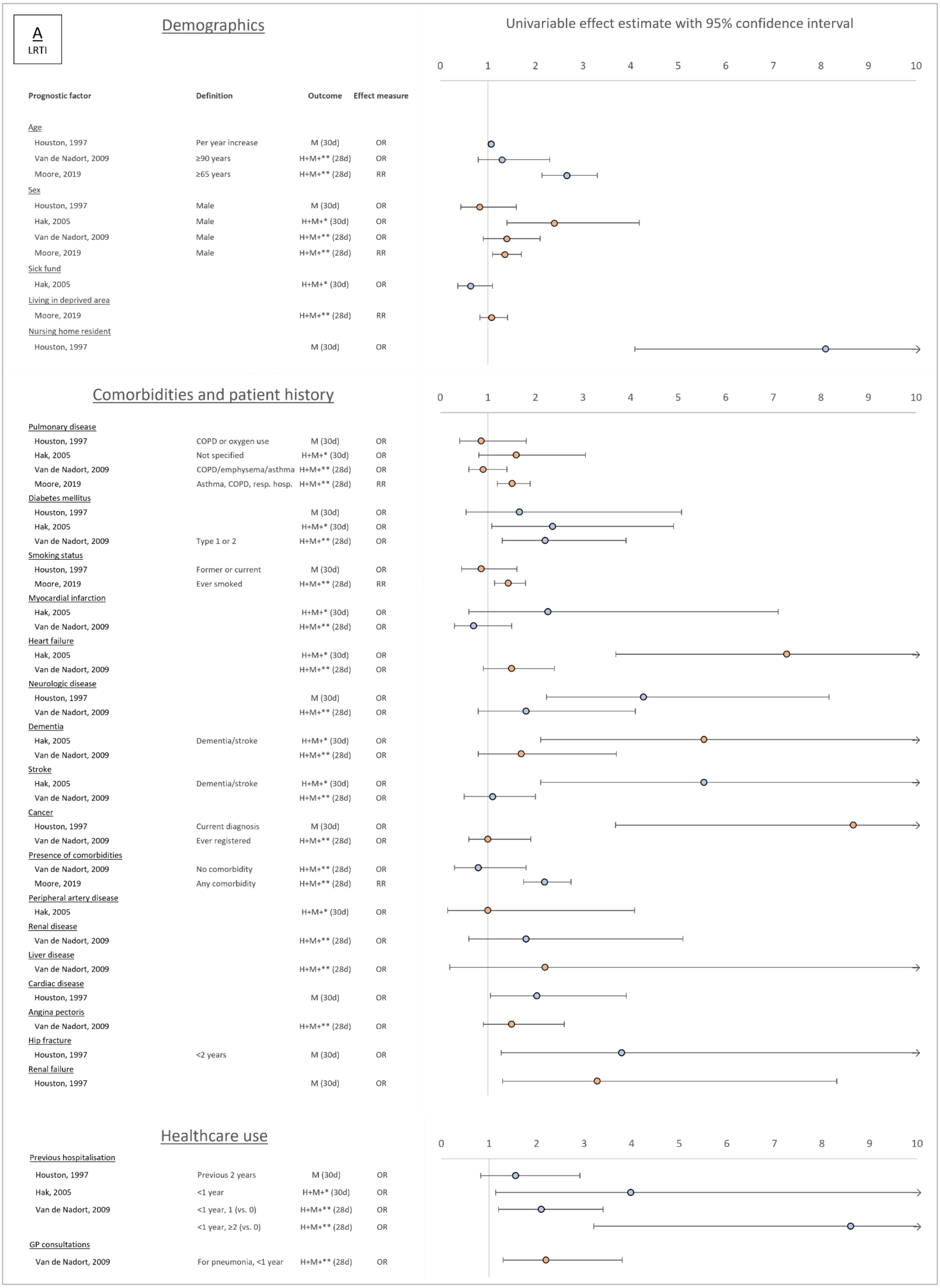

S4, continued

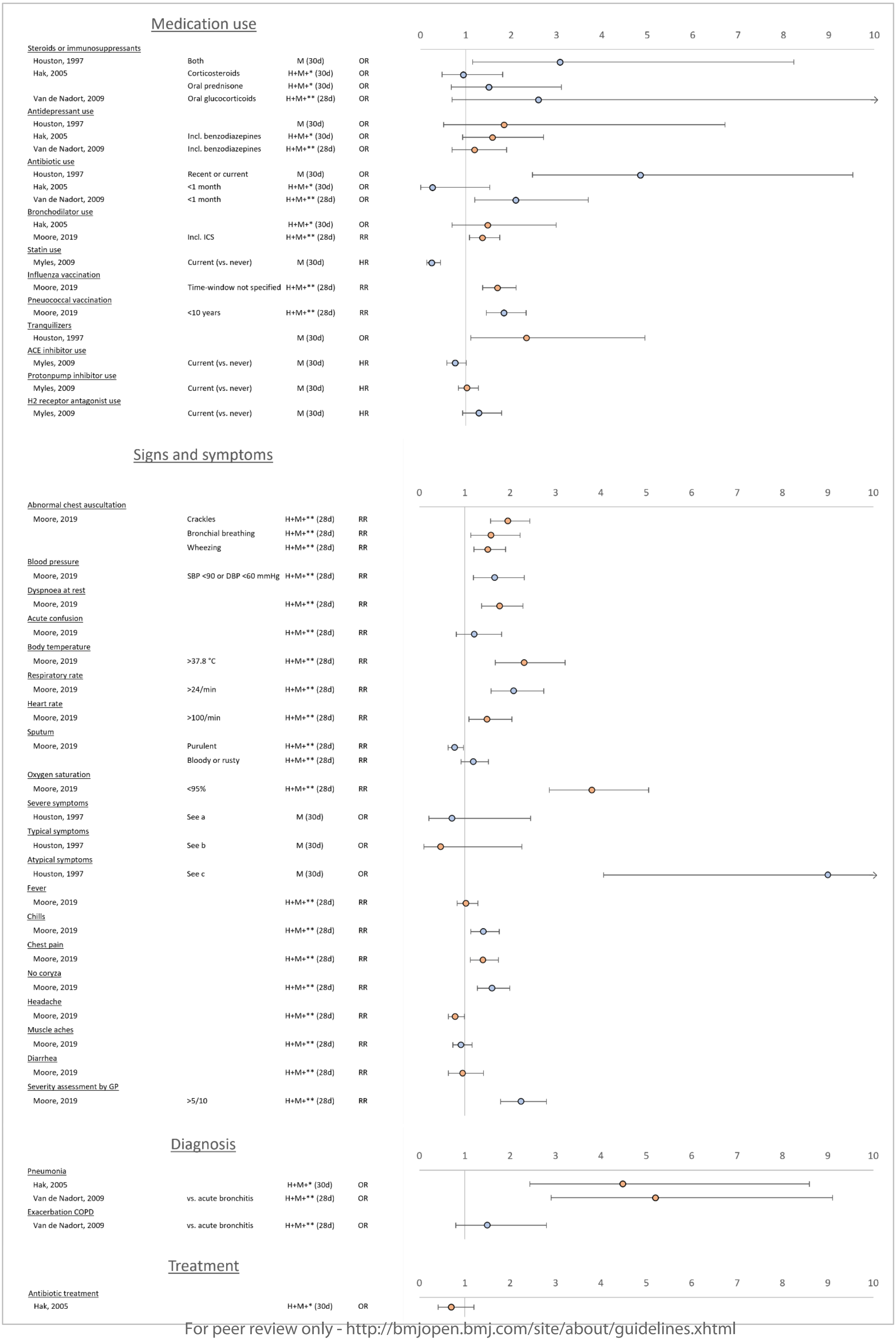

S4, continued

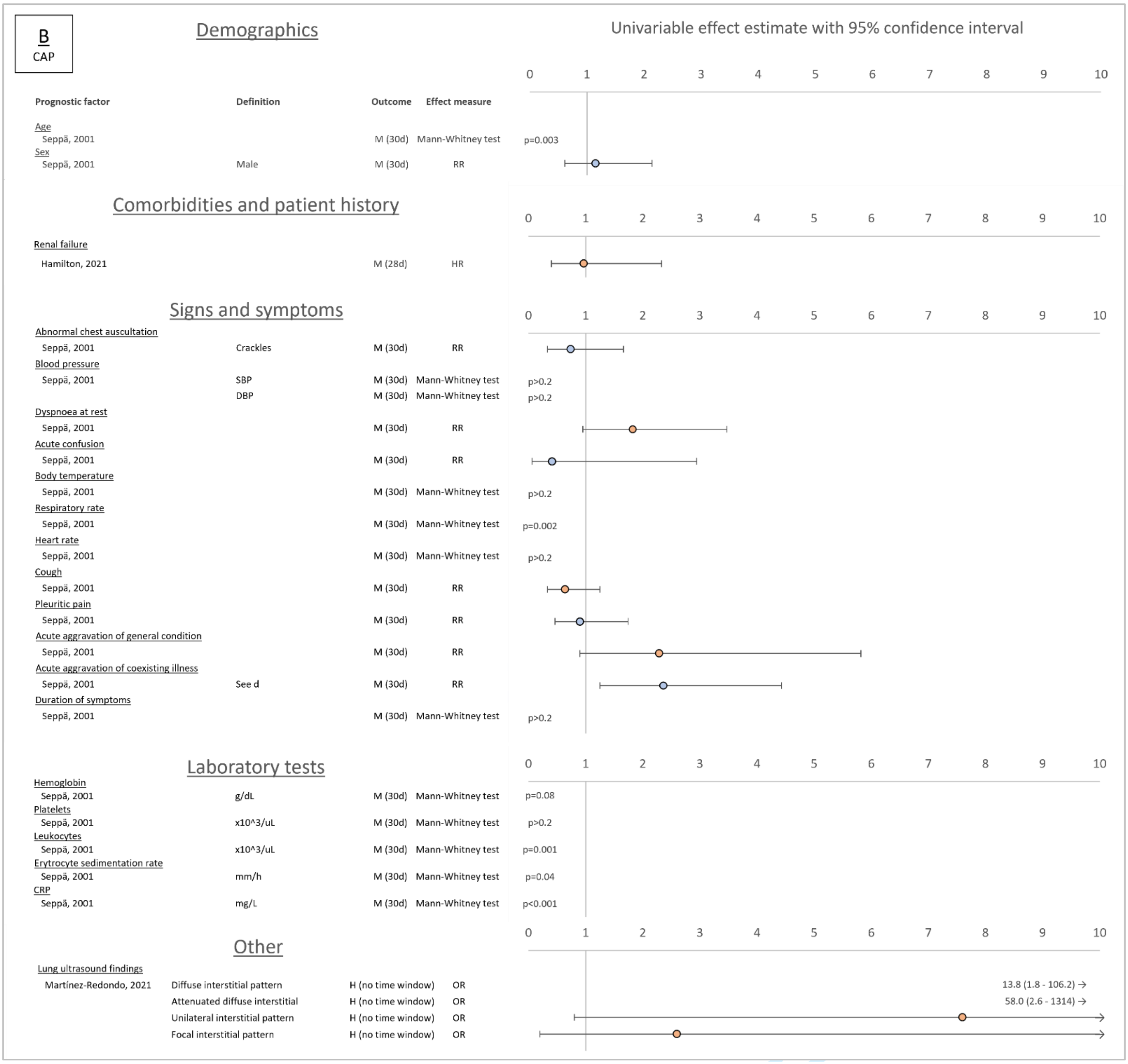

A. Univariable analyses of prognostic factors from studies on LRTI patients. B. Univariable analysis of prognostic factors from studies on pneumonia patients.
* Composite outcome also includes dysregulation of diabetes, stroke, heart failure, MI.
** Composite outcome also includes late onset pneumonia.
a: rigor, hemoptysis, pleuritic pain.
b: chills, cough, congestion, fever, dyspnea, sputum production.
c: poor eating, confusion, lethargy.
d: impairment of glucose balance in diabetic patients, deterioration of congestive heart failure.
Abbreviations: LRTI, lower respiratory tract infection; H, hospitalisation; M, mortality; OR, odds ratio; RR, risk ratio; HR, hazard ratio; COPD, chronic obstructive pulmonary disease; GP, general practitioner; ICS, inhalation corticosteroids; ACE, angiotensin converting enzyme; SBP, systolic blood pressure; DBP, diastolic blood pressure; min, minute; g, gram; dL, deciliter; uL, microliter; mm, millimeter; h, hour; L, liter.

S5: figure of all PF effect estimates based on multivariable effect estimates

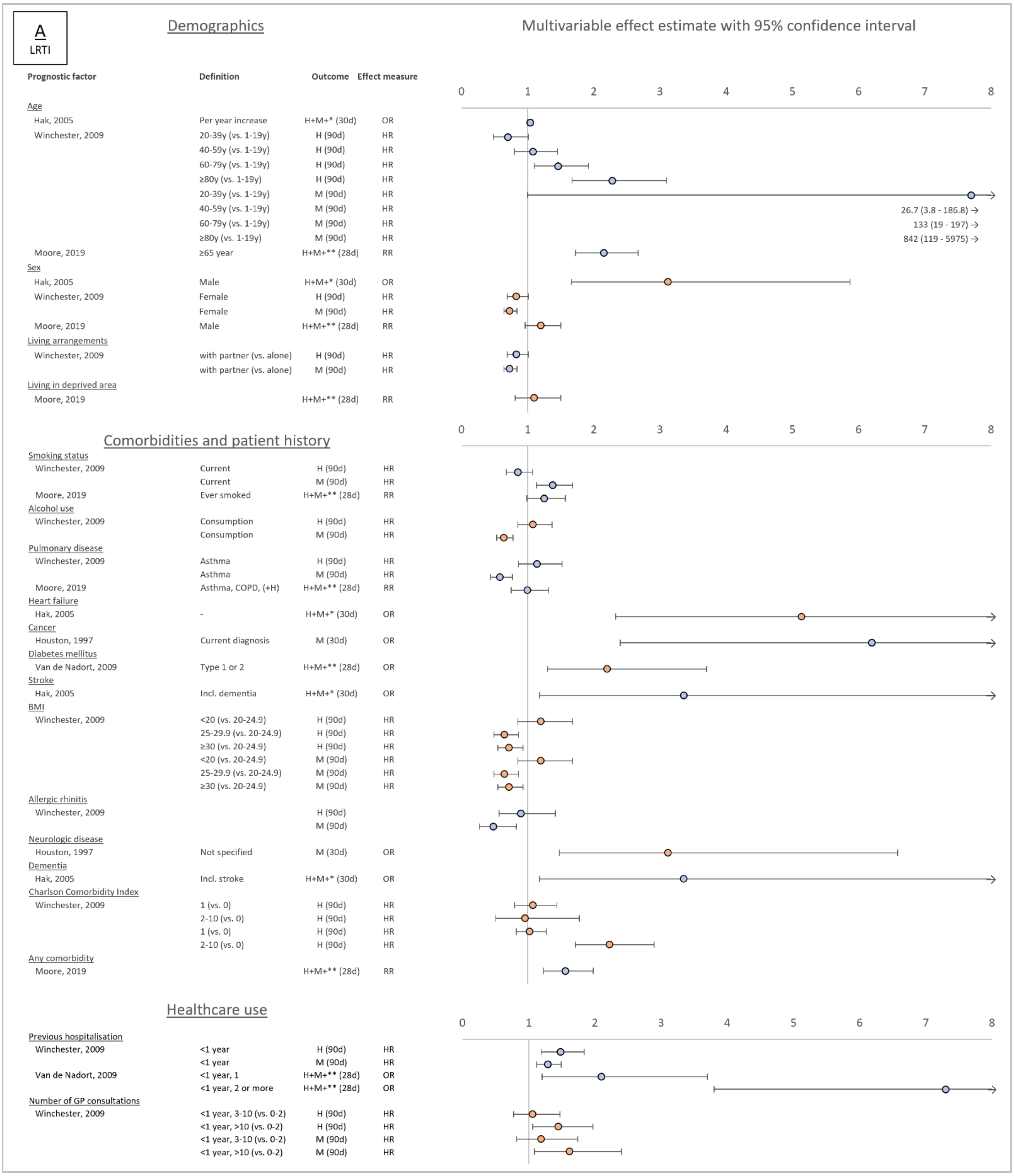

Figure S5, continued

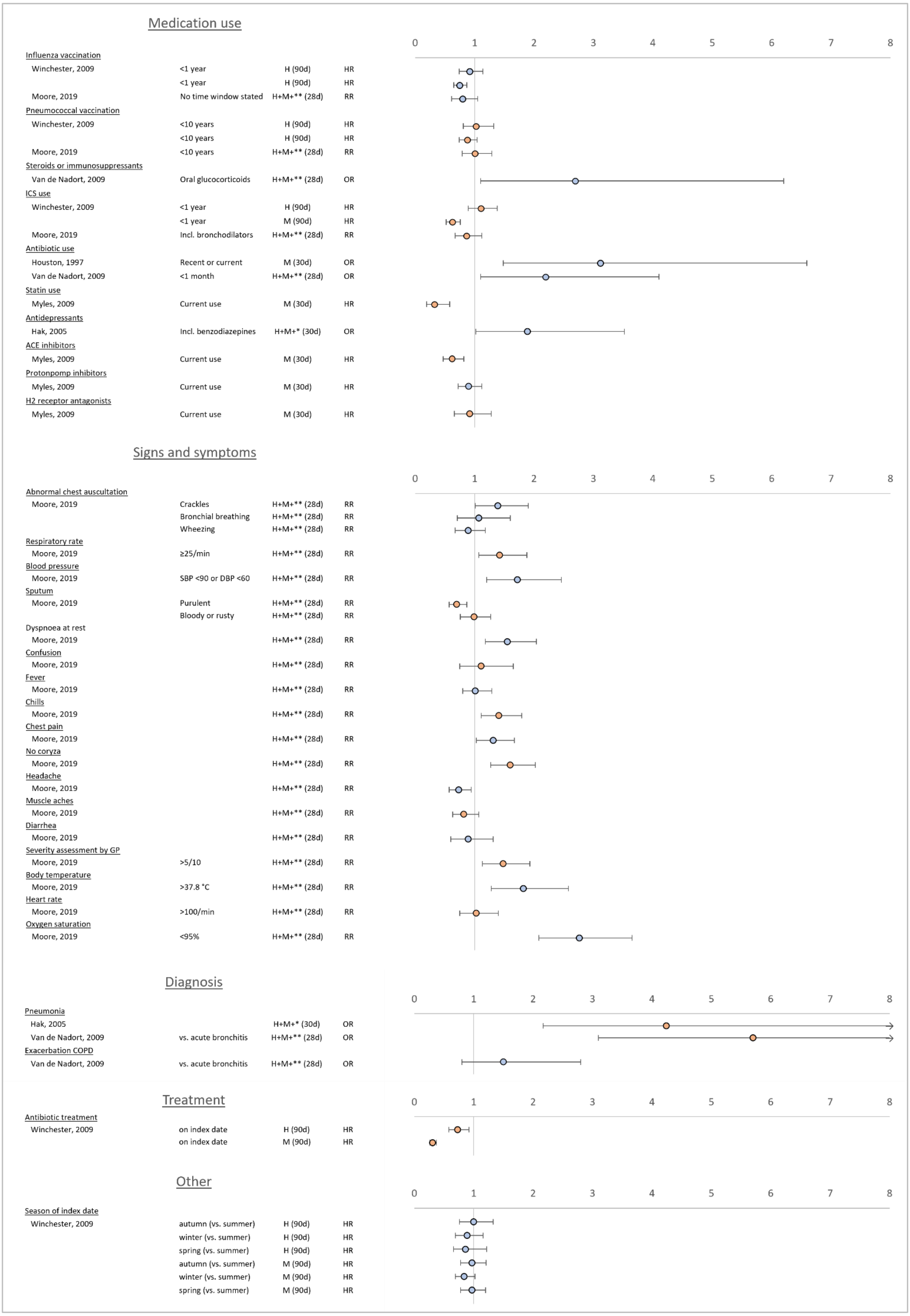

S5, continued

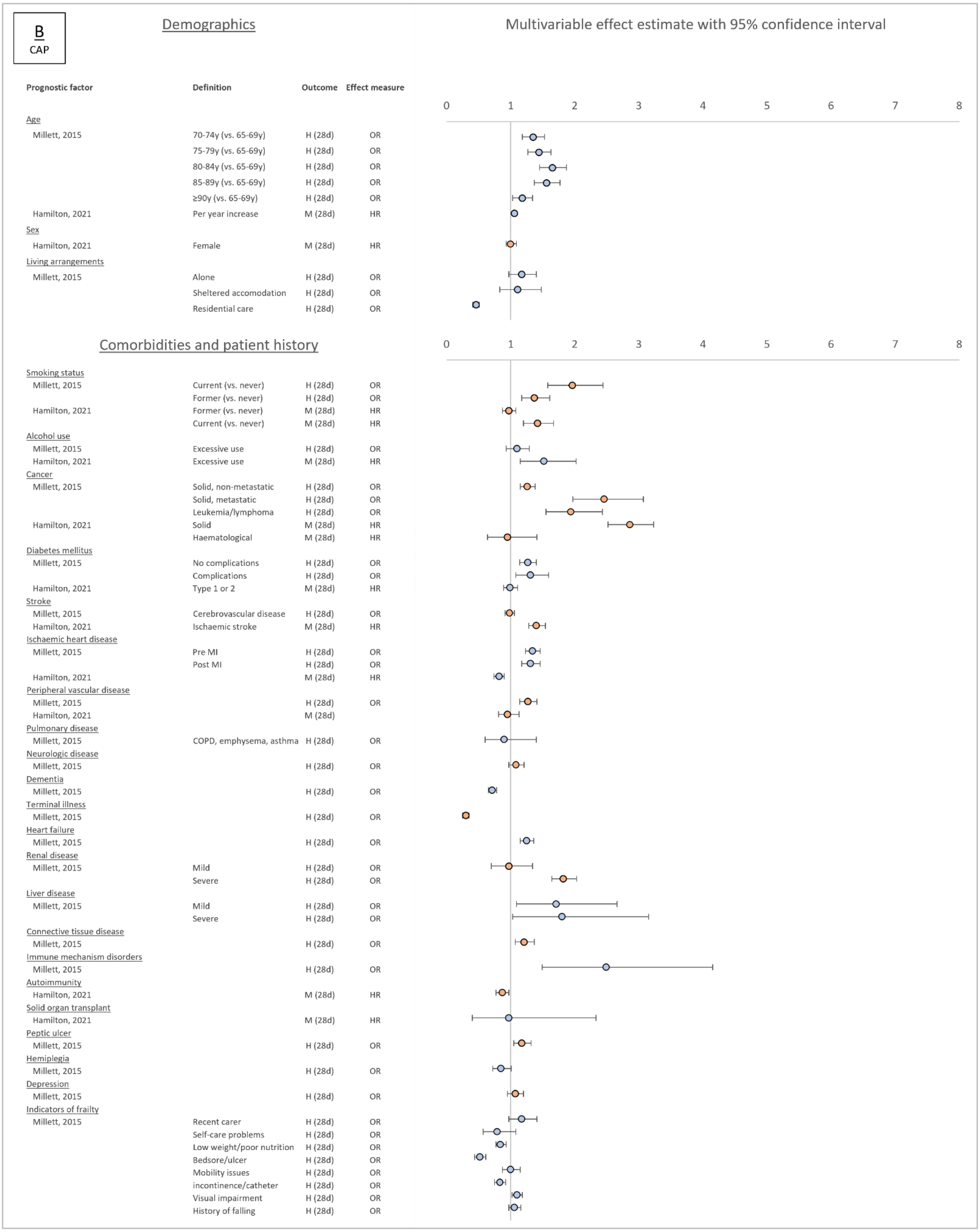

S5, continued

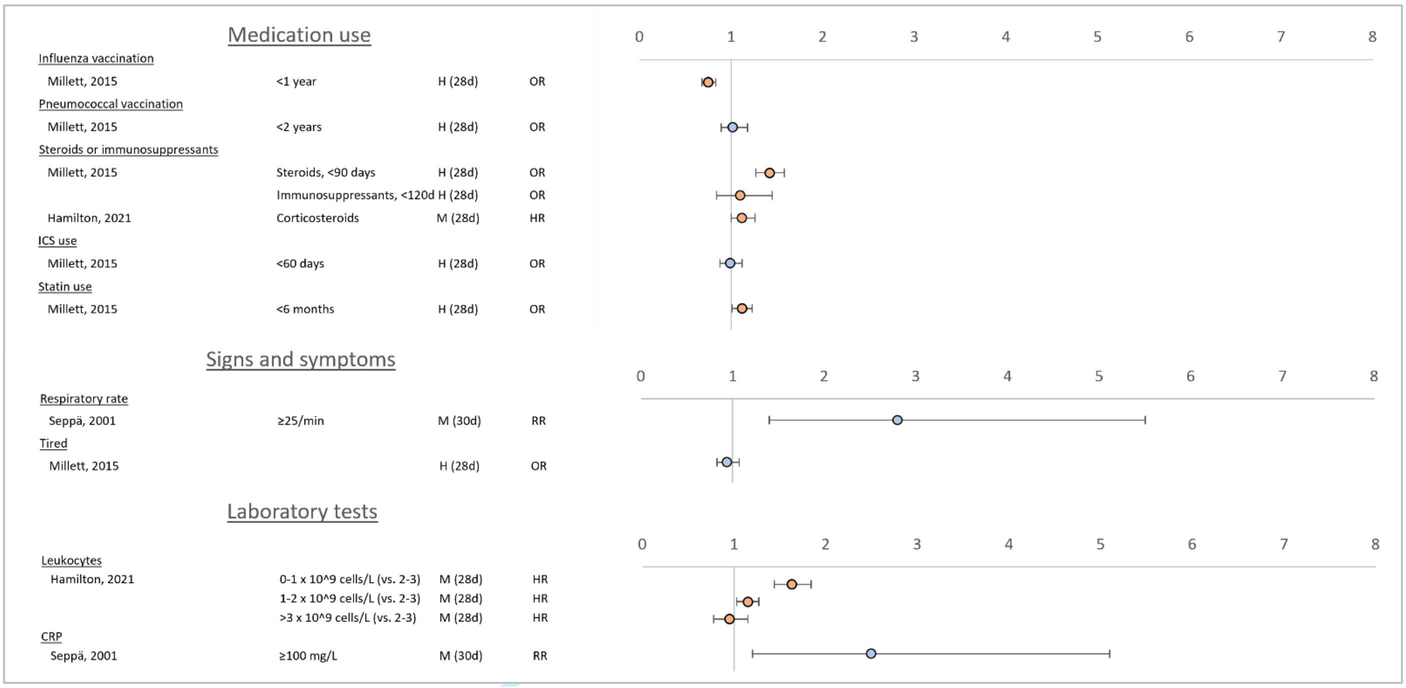

A. Multivariable analyses of prognostic factors from studies on LRTI patients. B. Multivariable analysis of prognostic factors from studies on pneumonia patients.
* Composite outcome also includes dysregulation of diabetes, stroke, heart failure, MI.
** Composite outcome also includes late onset pneumonia.
Abbreviations: LRTI, lower respiratory tract infection; H, hospitalisation; M, mortality; OR, odds ratio; RR, risk ratio; HR, hazard ratio; COPD, chronic obstructive pulmonary disease; BMI, body mass index; MI, myocardial infarction; GP, general practitioner; ICS, inhalation corticosteroids; ACE, angiotensin converting enzyme; SBP, systolic blood pressure; DBP, diastolic blood pressure; min, minute; L, liter; mg, milligram.

For peer review only

Table S6: Rating of the quality of evidence on promising prognostic factors based on the GRADE framework

Prognostic factor	GRADE criteria							Quality of evidence
	Study design	Risk of bias	Inconsistency	Indirectness	Imprecision	Publication bias	Upgrading factors	
Age	Cohort (high quality)	-2	0	0	0	0	0	Low
Sex	Cohort (high quality)	-2	0	-1	-1	0	0	Very low
Smoking status	Cohort (high quality)	-2	-1	-1	-1	0	0	Very low
Diabetes	Cohort (high quality)	-2	0	-1	0	0	0	Very low
Stroke	Cohort (high quality)	-2	-1	-1	-1	0	0	Very low
Cancer	Cohort (high quality)	-2	0	-1	0	0	0	Very low
Heart failure	Cohort (high quality)	-2	0	-1	0	0	0	Very low
Previous hospitalisation	Cohort (high quality)	-2	0	0	0	0	0	Low
Systemic corticosteroids	Cohort (high quality)	-2	0	-1	-1	0	0	Very low
Influenza vaccination	Cohort (high quality)	-2	0	0	-1	0	0	Very low
Recent antibiotic use	Cohort (high quality)	-2	0	0	-1	0	0	Very low
Respiratory rate	Cohort (high quality)	-2	0	-1	0	0	0	Very low
Diagnosis of pneumonia	Cohort (high quality)	-2	0	0	0	0	0	Low

Certainty of evidence			
High	Moderate	Low	Very low
4	3	2	1

Application of the GRADE framework to rate the quality of evidence was based on prognostic research-specific guidance (Foroutan et al., 2020).

Abbreviations: GRADE, grading of recommendations, assessment, development, and evaluations.

For peer review only

Table S7: Overview of prediction models included in the synthesis

Prediction model	Predictors included in model	Predictor weight
Bont, 2007 (new)	- Diagnosis (either acute bronchitis, COPD exacerbation, or pneumonia)	0, 2, or 4
	- Age ≥80 years	2
	- Congestive heart failure	1
	- Diabetes	2
	- Oral glucocorticoid use	3
	- Hospitalisations in previous year (0, 1, or ≥2)	0, 2, or 3
	- Antibiotic use in previous month	2
CRB(-65)	- Confusion	1
	- Respiratory rate ≥30/minute	1
	- Blood pressure (SBP ≤90mmHg or DBP ≤60mmHg)	1
	(- Age ≥65 years)	1
CURB(-65)	- Predictors of CRB(-65)	0-4
	- Blood urea nitrogen >7 mmol/L	1
PSI (stage 1)	- Age >50 years	Any versus none
	- Altered mental status	
	- Pulse >125/minute	
	- Respiratory rate >30/minute	
	- SBP <90mmHg	
	- Temperature <35°C or ≥40°C	
	- Neoplastic disease	
	- Congestive heart failure	
	- Cerebrovascular disease	
	- Renal disease	
	- Liver disease	
CORB-75 (new)	- Confusion	1
	- Peripheral oxygen saturation ≤90%	1
	- Respiratory rate ≥30/minute	1
	- Blood pressure (SBP ≤90mmHg or DBP ≤60mmHg)	1
	- Age ≥75 years	1
RISSC85 (new)	- Risk of poor outcome, grouped by country (A: Spain, B: Belgium, the Netherlands, Poland, UK, C: Germany)	A = 0, B/C = 1
	- Interference in daily activities (some versus severe)	1
	- Number of years stopped smoking (>45 years)	1
	- Severe sputum	1
	- Presence of crackles	1
	- Diastolic blood pressure (<85mmHg)	1
Moore, 2019 (new)	- Oxygen saturation <95%	1
	- Age >65 years	1
	- Blood pressure (SBP <90mmHg or DBP <60mmHg)	1
	- Temperature >37.8°C	1
	- Any comorbidity (cardiovascular, cerebrovascular or lung comorbidities)	1
	- No coryza	1
	- Severity assessment >5/10 by GP	1
	- Chest pain	1

Abbreviations: COPD, chronic obstructive pulmonary disease; SBP, systolic blood pressure; DBP, diastolic blood pressure; GP, general practitioner.

For peer review only

For peer review only - http://bmjopen.bmj.com/site/about/guidelines.xhtml

PRISMA 2020 Checklist

Section and Topic	Item #	Checklist item	Location where item is reported
**TITLE**			
Title	1	Identify the report as a systematic review.	1
**ABSTRACT**			
Abstract	2	See the PRISMA 2020 for Abstracts checklist.	3
**INTRODUCTION**			
Rationale	3	Describe the rationale for the review in the context of existing knowledge.	6
Objectives	4	Provide an explicit statement of the objective(s) or question(s) the review addresses.	6
**METHODS**			
Eligibility criteria	5	Specify the inclusion and exclusion criteria for the review and how studies were grouped for the syntheses.	7
Information sources	6	Specify all databases, registers, websites, organisations, reference lists and other sources searched or consulted to identify studies. Specify the date when each source was last searched or consulted.	7
Search strategy	7	Present the full search strategies for all databases, registers and websites, including any filters and limits used.	S1
Selection process	8	Specify the methods used to decide whether a study met the inclusion criteria of the review, including how many reviewers screened each record and each report retrieved, whether they worked independently, and if applicable, details of automation tools used in the process.	7
Data collection process	9	Specify the methods used to collect data from reports, including how many reviewers collected data from each report, whether they worked independently, any processes for obtaining or confirming data from study investigators, and if applicable, details of automation tools used in the process.	7, 8
Data items	10a	List and define all outcomes for which data were sought. Specify whether all results that were compatible with each outcome domain in each study were sought (e.g. for all measures, time points, analyses), and if not, the methods used to decide which results to collect.	7, 8
	10b	List and define all other variables for which data were sought (e.g. participant and intervention characteristics, funding sources). Describe any assumptions made about any missing or unclear information.	8
Study risk of bias assessment	11	Specify the methods used to assess risk of bias in the included studies, including details of the tool(s) used, how many reviewers assessed each study and whether they worked independently, and if applicable, details of automation tools used in the process.	8
Effect measures	12	Specify for each outcome the effect measure(s) (e.g. risk ratio, mean difference) used in the synthesis or presentation of results.	7, 8
Synthesis methods	13a	Describe the processes used to decide which studies were eligible for each synthesis (e.g. tabulating the study intervention characteristics and comparing against the planned groups for each synthesis (item #5)).	7, 8
	13b	Describe any methods required to prepare the data for presentation or synthesis, such as handling of missing summary statistics, or data conversions.	7, 8
	13c	Describe any methods used to tabulate or visually display results of individual studies and syntheses.	8, 9
	13d	Describe any methods used to synthesize results and provide a rationale for the choice(s). If meta-analysis was performed, describe the model(s), method(s) to identify the presence and extent of statistical heterogeneity, and software package(s) used.	8, 9
	13e	Describe any methods used to explore possible causes of heterogeneity among study results (e.g. subgroup analysis, meta-regression).	N/A
	13f	Describe any sensitivity analyses conducted to assess robustness of the synthesized results.	N/A
Reporting bias assessment	14	Describe any methods used to assess risk of bias due to missing results in a synthesis (arising from reporting biases).	N/A
Certainty	15	Describe any methods used to assess certainty (or confidence) in the body of evidence for an outcome.	N/A

For peer review only

For peer review only - http://bmjopen.bmj.com/site/about/guidelines.xhtml

PRISMA 2020 Checklist

Section and Topic	Item #	Checklist item	Location where item is reported
assessment			
**RESULTS**			
Study selection	16a	Describe the results of the search and selection process, from the number of records identified in the search to the number of studies included in the review, ideally using a flow diagram.	10, Figure 1
	16b	Cite studies that might appear to meet the inclusion criteria, but which were excluded, and explain why they were excluded.	N/A
Study characteristics	17	Cite each included study and present its characteristics.	10, Table 2-3
Risk of bias in studies	18	Present assessments of risk of bias for each included study.	11, Figure S2-3
Results of individual studies	19	For all outcomes, present, for each study: (a) summary statistics for each group (where appropriate) and (b) an effect estimate and its precision (e.g. confidence/credible interval), ideally using structured tables or plots.	11-13, Figure 2-3, Figure S4-5
Results of syntheses	20a	For each synthesis, briefly summarise the characteristics and risk of bias among contributing studies.	11
	20b	Present results of all statistical syntheses conducted. If meta-analysis was done, present for each the summary estimate and its precision (e.g. confidence/credible interval) and measures of statistical heterogeneity. If comparing groups, describe the direction of the effect.	11-13, Figure 2-3, Figure S4-5
	20c	Present results of all investigations of possible causes of heterogeneity among study results.	N/A
	20d	Present results of all sensitivity analyses conducted to assess the robustness of the synthesized results.	N/A
Reporting biases	21	Present assessments of risk of bias due to missing results (arising from reporting biases) for each synthesis assessed.	N/A
Certainty of evidence	22	Present assessments of certainty (or confidence) in the body of evidence for each outcome assessed.	N/A
**DISCUSSION**			
Discussion	23a	Provide a general interpretation of the results in the context of other evidence.	14, 15
	23b	Discuss any limitations of the evidence included in the review.	16
	23c	Discuss any limitations of the review processes used.	16
	23d	Discuss implications of the results for practice, policy, and future research.	17
**OTHER INFORMATION**			
Registration and protocol	24a	Provide registration information for the review, including register name and registration number, or state that the review was not registered.	3, 7
	24b	Indicate where the review protocol can be accessed, or state that a protocol was not prepared.	3, 7
	24c	Describe and explain any amendments to information provided at registration or in the protocol.	N/A
Support	25	Describe sources of financial or non-financial support for the review, and the role of the funders or sponsors in the review.	18
Competing interests	26	Declare any competing interests of review authors.	18
Availability of data, code and other materials	27	Report which of the following are publicly available and where they can be found: template data collection forms; data extracted from included studies; data used for all analyses; analytic code; any other materials used in the review.	18

For peer review only - http://bmjopen.bmj.com/site/about/guidelines.xhtml

PRISMA 2020 Checklist

From: Page MJ, McKenzie JE, Bossuyt PM, Boutron I, Hoffmann TC, Mulrow CD, et al. The PRISMA 2020 statement: an updated guideline for reporting systematic reviews. BMJ 2021;372:n71. doi: 10.1136/bmj.n71

For more information, visit: http://www.prisma-statement.org/

For peer review only

PROSPERO
International prospective register of systematic reviews

NHS
National Institute for Health Research

UNIVERSITY of York
Centre for Reviews and Dissemination

Systematic review

A list of fields that can be edited in an update can be found here

1. * Review title.

Give the title of the review in English

Prognosis of lower respiratory tract infections in adults presenting to primary care: a systematic review of prognostic factors and prediction models

2. Original language title.

For reviews in languages other than English, give the title in the original language. This will be displayed with the English language title.

3. * Anticipated or actual start date.

Give the date the systematic review started or is expected to start.

17/06/2022

4. * [1 change] Anticipated completion date.

Give the date by which the review is expected to be completed.

01/04/2023

5. * [3 changes] Stage of review at time of this submission.

This field uses answers to initial screening questions. It cannot be edited until after registration.

Tick the boxes to show which review tasks have been started and which have been completed.

Update this field each time any amendments are made to a published record.

The review has not yet started: No

For peer review only

PROSPERO
International prospective register of systematic reviews

National Institute for Health Research

Review stage	Started	Completed
Preliminary searches	Yes	Yes
Piloting of the study selection process	Yes	Yes
Formal screening of search results against eligibility criteria	Yes	Yes
Data extraction	Yes	Yes
Risk of bias (quality) assessment	Yes	Yes
Data analysis	Yes	Yes

Provide any other relevant information about the stage of the review here.

6. * Named contact.

The named contact is the guarantor for the accuracy of the information in the register record. This may be any member of the review team.

Merijn Rijk

Email salutation (e.g. "Dr Smith" or "Joanne") for correspondence:

Merijn Rijk

7. * Named contact email.

Give the electronic email address of the named contact.

m.h.rijk@umcutrecht.nl

8. Named contact address

Give the full institutional/organisational postal address for the named contact.

P.O. Box 85500, 3508 GA Utrecht

9. Named contact phone number.

Give the telephone number for the named contact, including international dialling code.

0031647424873

10. * Organisational affiliation of the review.

Full title of the organisational affiliations for this review and website address if available. This field may be

For peer review only

completed as 'None' if the review is not affiliated to any organisation.

Julius Center for Health Sciences and Primary Care, UMC Utrecht

Organisation web address:

11. * Review team members and their organisational affiliations. [1 change]

Give the personal details and the organisational affiliations of each member of the review team. Affiliation refers to groups or organisations to which review team members belong. **NOTE: email and country now MUST be entered for each person, unless you are amending a published record.**

Mr Merijn Rijk. Julius Center for Health Sciences and Primary Care, UMC Utrecht
Mr Teun van den Berg. Julius Center for Health Sciences and Primary Care, UMC Utrecht
Dr Tamara Platteel. Julius Center for Health Sciences and Primary Care, UMC Utrecht
Dr Roderick Venekamp. Julius Center for Health Sciences and Primary Care, UMC Utrecht
Dr Maarten van Smeden. Julius Center for Health Sciences and Primary Care, UMC Utrecht
Dr Geert-Jan Geersing. Julius Center for Health Sciences and Primary Care, UMC Utrecht
Professor Frans Rutten. Julius Center for Health Sciences and Primary Care, UMC Utrecht
Dr Monika Hollander. Julius Center for Health Sciences and Primary Care, UMC Utrecht
Professor Paul Little. University of Southampton
Dr Bert Dalmolen.

12. * Funding sources/sponsors.

Details of the individuals, organizations, groups, companies or other legal entities who have funded or sponsored the review.

The Netherlands Organisation for Health Research and Development (ZonMw), Huisartsgeneeskunde 2021, no. 08391052110003

Grant number(s)

State the funder, grant or award number and the date of award

ZonMw, General Practice and Elderly Care grant, round of 2021

13. * Conflicts of interest.

List actual or perceived conflicts of interest (financial or academic).

None

14. Collaborators.

Give the name and affiliation of any individuals or organisations who are working on the review but who are not listed as review team members. **NOTE: email and country must be completed for each person, unless you are amending a published record.**

For peer review only

PROSPERO
International prospective register of systematic reviews

National Institute for Health Research

15. * Review question. [1 change]

State the review question(s) clearly and precisely. It may be appropriate to break very broad questions down into a series of related more specific questions. Questions may be framed or refined using PI(E)COS or similar where relevant.

1) What are prognostic factors for a complicated trajectory (i.e. hospitalization or death) within 90 days in adult patients with lower respiratory tract infections (LRTI) presenting to primary care?

P: adult patients with LRTI

I: any patient or disease specific predictor

C: -

O: Hospitalisation or death

T1: prognostic factor present at first consultation of LRTI episode

T2: outcome within 90 days

S: primary care

2) What prediction models have been developed to estimate individual risk of hospitalization and/or death within 90 days in adult patients with a LRTI presenting to primary care?

P: adult patients with LRTI

I: any developed prediction model

C: -

O: Hospitalisation or death

T1: prediction at first consultation of LRTI episode

T2: outcome within 90 days

S: primary care

16. * Searches.

State the sources that will be searched (e.g. Medline). Give the search dates, and any restrictions (e.g. language or publication date). Do NOT enter the full search strategy (it may be provided as a link or attachment below.)

For peer review only

MEDLINE, Embase, and Cochrane Library. Restrictions: no restrictions in search strategy.

17. URL to search strategy.

Upload a file with your search strategy, or an example of a search strategy for a specific database, (including the keywords) in pdf or word format. In doing so you are consenting to the file being made publicly accessible. Or provide a URL or link to the strategy. Do NOT provide links to your search **results**.

https://www.crd.york.ac.uk/PROSPEROFILES/341233_STRATEGY_20220628.pdf

Alternatively, upload your search strategy to CRD in pdf format. Please note that by doing so you are consenting to the file being made publicly accessible.

Do not make this file publicly available until the review is complete

18. * Condition or domain being studied.

Give a short description of the disease, condition or healthcare domain being studied in your systematic review.

Prognosis of lower respiratory tract infections in adults presenting to primary care

19. * Participants/population.

Specify the participants or populations being studied in the review. The preferred format includes details of both inclusion and exclusion criteria.

Adults with lower respiratory tract infections presenting to primary care

20. * Intervention(s), exposure(s).

Give full and clear descriptions or definitions of the interventions or the exposures to be reviewed. The preferred format includes details of both inclusion and exclusion criteria.

For review question 1): any patient or disease specific prognostic factor
For review question 2): any developed prediction model applicable to a primary care setting

21. * Comparator(s)/control.

Where relevant, give details of the alternatives against which the intervention/exposure will be compared (e.g. another intervention or a non-exposed control group). The preferred format includes details of both inclusion and exclusion criteria.

Not relevant

22. * Types of study to be included.

Give details of the study designs (e.g. RCT) that are eligible for inclusion in the review. The preferred format includes both inclusion and exclusion criteria. If there are no restrictions on the types of study, this should be stated.

For review question 1): prognostic factor studies
For review question 2): model development and validation studies

For peer review only

PROSPERO
International prospective register of systematic reviews
National Institute for Health Research

23. Context.

Give summary details of the setting or other relevant characteristics, which help define the inclusion or exclusion criteria.

For review question 1):

- Prognostic factor studies conducted within (or applicable to) a primary care setting

For review question 2):

- Model development and/or validation studies conducted within (or applicable to) a primary care setting
- Validation or updating of secondary care derived prediction models in primary care

24. * Main outcome(s).

Give the pre-specified main (most important) outcomes of the review, including details of how the outcome is defined and measured and when these measurement are made, if these are part of the review inclusion criteria.

Hospitalisation or (all-cause) death within 90 days after initial presentation to primary care

Measures of effect

Please specify the effect measure(s) for you main outcome(s) e.g. relative risks, odds ratios, risk difference, and/or 'number needed to treat.

For review question 1): prognostic effect measures (odds ratios and hazard ratios), both unadjusted and adjusted

For review question 2): data on discrimination (C statistic) and calibration (O:E ratio, intercept, slope)

25. * Additional outcome(s).

List the pre-specified additional outcomes of the review, with a similar level of detail to that required for main outcomes. Where there are no additional outcomes please state ‘None’ or ‘Not applicable’ as appropriate to the review

Not applicable

Measures of effect

Please specify the effect measure(s) for you additional outcome(s) e.g. relative risks, odds ratios, risk difference, and/or 'number needed to treat.

26. * Data extraction (selection and coding).

Describe how studies will be selected for inclusion. State what data will be extracted or obtained. State how this will be done and recorded.

Study selection:

1) Clinical domain, outcome, and setting match PICOTS, study design: prognostic factor study or prognostic model development or validation study

For peer review only

- Exclusion: full-text not available, language other than English or Dutch

2) Screening for eligible studies by two researchers, independently and blinded. Screening process: 1) title/abstract, 2) full-text, 3) backward and forward citation searching of included studies.

3) Disagreements resolved through discussion and, if necessary, after consultation of third researcher (supervisor)

4) Software system: de-duplication of yielded studies and title/abstract screening using Rayyan (online application)

Data extraction:

1) Data extraction based on CHARMS (prediction models) and CHARMS-PF (prognostic factor studies) checklists

2) Extraction of data by two researchers: extraction of data by researcher 1, check of extracted data by researcher 2

3) Disagreements will be resolved through discussion and, if neccesary, after consultation of a third researcher (supervisor)

4) Missing data on measurements of effect will be either calculated from raw data if possible or requested from researchers of the primary study

27. * Risk of bias (quality) assessment.

State which characteristics of the studies will be assessed and/or any formal risk of bias/quality assessment tools that will be used.

For risk of bias assessment, the following formal tools will be used:

- For review question 1): QUIPS

- For review question 2): PROBAST

28. * Strategy for data synthesis.

[1 change]

Describe the methods you plan to use to synthesise data. This **must not be generic text** but should be **specific to your review** and describe how the proposed approach will be applied to your data. If meta-analysis is planned, describe the models to be used, methods to explore statistical heterogeneity, and software package to be used.

For review question 1):

For peer review only

Data synthesis will not be performed for prognostic factors due to 1) the broad search for 'any' prognostic factor and 2) the anticipated heterogeneity of the domain and prognostic factor definition. Effect measures (unadjusted and adjusted) of prognostic factors will be summarised in a forest plot.

For review question 2):

Data synthesis will not be performed. Prediction models will be evaluated and compared based on:

- Risk of bias in the development (using PROBAST tool)

- Applicability to the clinical patient and setting (PICOTS and review question)

- Performance of the model (based on the performance measures mentioned in field #24)

- Method of validation (internal, internal-external, external).

Based on these criteria conclusions will be drawn with regard to the usefulness of the prediction models to clinical practice. This will provide guidance for clinicians to choose the appropriate tool (if any of the prediction models proves to be robust) for prediction of hospitalization or death in LRTI patients.

29. * Analysis of subgroups or subsets.

State any planned investigation of ‘subgroups’. Be clear and specific about which type of study or participant will be included in each group or covariate investigated. State the planned analytic approach.

Studies for both review questions will be stratified with regard to data collection on (possible) Coronavirus disease 2019 (COVID-19) patients, to assess any COVID-19 specific effect on results:

- Non-COVID-19 data (data collection up to and including 2019 and data on (explicitly stated) non-COVID-19 patients collected from 2020 onwards)

- COVID-19 data (data collection from 2020 onwards on (possible) COVID-19 patients)

30. * Type and method of review.

Select the type of review, review method and health area from the lists below.

Type of review

Cost effectiveness
No

Diagnostic
No

Epidemiologic
No

Individual patient data (IPD) meta-analysis

No

Intervention

No

Living systematic review

No

Meta-analysis

No

Methodology

No

Narrative synthesis

No

Network meta-analysis

No

Pre-clinical

No

Prevention

No

Prognostic

Yes

Prospective meta-analysis (PMA)

No

Review of reviews

No

Service delivery

No

Synthesis of qualitative studies

No

Systematic review

Yes

Other

No

Health area of the review

Alcohol/substance misuse/abuse

No

Blood and immune system

For peer review only

No

Cancer

No

Cardiovascular

No

Care of the elderly

No

Child health

No

Complementary therapies

No

COVID-19

Yes

For COVID-19 registrations please tick all categories that apply. Doing so will enable your record to appear in area-specific searches

Chinese medicine
Diagnosis
Epidemiological
Genetics
Health impacts
Immunity
Long COVID
Mental health
PPE
Prognosis
Public health intervention
Rehabilitation
Service delivery
Transmission
Treatments
Vaccines
Other

Crime and justice

No

Dental

No

Digestive system

No

Ear, nose and throat

No

For peer review only

PROSPERO
International prospective register of systematic reviews

National Institute for Health Research

Education
No

Endocrine and metabolic disorders
No

Eye disorders
No

General interest
No

Genetics
No

Health inequalities/health equity
No

Infections and infestations
Yes

International development
No

Mental health and behavioural conditions
No

Musculoskeletal
No

Neurological
No

Nursing
No

Obstetrics and gynaecology
No

Oral health
No

Palliative care
No

Perioperative care
No

Physiotherapy
No

Pregnancy and childbirth
No

Public health (including social determinants of health)

For peer review only

No

Rehabilitation

No

Respiratory disorders

No

Service delivery

No

Skin disorders

No

Social care

No

Surgery

No

Tropical Medicine

No

Urological

No

Wounds, injuries and accidents

No

Violence and abuse

No

31. Language.

Select each language individually to add it to the list below, use the bin icon to remove any added in error.

English

There is not an English language summary

32. * Country.

Select the country in which the review is being carried out. For multi-national collaborations select all the countries involved.

Netherlands

33. Other registration details.

Name any other organisation where the systematic review title or protocol is registered (e.g. Campbell, or The Joanna Briggs Institute) together with any unique identification number assigned by them. If extracted data will be stored and made available through a repository such as the Systematic Review Data Repository (SRDR), details and a link should be included here. If none, leave blank.

For peer review only

PROSPERO
International prospective register of systematic reviews

National Institute for Health Research

34. Reference and/or URL for published protocol.

If the protocol for this review is published provide details (authors, title and journal details, preferably in Vancouver format)

Add web link to the published protocol.

Or, upload your published protocol here in pdf format. Note that the upload will be publicly accessible.

No I do not make this file publicly available until the review is complete

Please note that the information required in the PROSPERO registration form must be completed in full even if access to a protocol is given.

35. Dissemination plans.

Do you intend to publish the review on completion?

Yes

Give brief details of plans for communicating review findings.?

36. Keywords.

Give words or phrases that best describe the review. Separate keywords with a semicolon or new line. Keywords help PROSPERO users find your review (keywords do not appear in the public record but are included in searches). Be as specific and precise as possible. Avoid acronyms and abbreviations unless these are in wide use.

Systematic review; prognosis; prognostic factor; prediction model; lower respiratory tract infection; adults; primary care; general practice; hospitalisation; mortality; complications.

37. Details of any existing review of the same topic by the same authors.

If you are registering an update of an existing review give details of the earlier versions and include a full bibliographic reference, if available.

38. * Current review status.
[1. change]

Update review status when the review is completed and when it is published.New registrations must be ongoing so this field is not editable for initial submission.

Please provide anticipated publication date

For peer review only

PROSPERO
International prospective register of systematic reviews

National Institute for Health Research

Review_Completed_not_published

39. Any additional information.

[1 change]

Provide any other information relevant to the registration of this review.

Additional information added after initial submission

- Included PICOTS components for review questions (field #15)

- Elaboration upon the assessment of prediction models and how these results help clinicians (GPs) to choose the appropriate tool (if any) for prediction of hospitalization or death in LRTI patients (field #28)

40. Details of final report/publication(s) or preprints if available.

Leave empty until publication details are available OR you have a link to a preprint (NOTE: this field is not editable for initial submission). List authors, title and journal details preferably in Vancouver format.

Give the link to the published review or preprint.

For peer review only

BMJ Open

Prognostic factors and prediction models for hospitalisation and all-cause mortality in adults presenting to primary care with a lower respiratory tract infection: a systematic review

Journal:	*BMJ Open*
Manuscript ID	bmjopen-2023-075475.R2
Article Type:	Original research
Date Submitted by the Author:	21-Feb-2024
Complete List of Authors:	Rijk, Merijn; University Medical Centre Utrecht, Julius Center for Health Sciences and Primary Care<br>Platteel, Tamara ; University Medical Centre Utrecht, Julius Center for Health Sciences and Primary Care<br>van den Berg, Teun; University Medical Centre Utrecht, Julius Center for Health Sciences and Primary Care<br>Geersing, Geert-Jan; Julius Center for Health Sciences and Primary Care, Department of General Practice<br>Little, Paul; University of Southampton, Primary Care and Population Science;<br>Rutten, Frans; Julius Centre for Health Sciences and Primary Care, University Medical Centre Utrecht, Department of General Practice<br>van Smeden, Maarten; University Medical Center Utrecht, Julius Center for Health Sciences and Primary Care<br>Venekamp , Roderick ; University Medical Center, Utrecht, Julius Center for Health Sciences and Primary Care
**Primary Subject Heading**:	Respiratory medicine
Secondary Subject Heading:	General practice / Family practice
Keywords:	Respiratory infections < THORACIC MEDICINE, Primary Care < Primary Health Care, Prognosis, Systematic Review

SCHOLARONE™
Manuscripts

I, the Submitting Author has the right to grant and does grant on behalf of all authors of the Work (as defined in the below author licence), an exclusive licence and/or a non-exclusive licence for contributions from authors who are: i) UK Crown employees; ii) where BMJ has agreed a CC-BY licence shall apply, and/or iii) in accordance with the terms applicable for US Federal Government officers or employees acting as part of their official duties; on a worldwide, perpetual, irrevocable, royalty-free basis to BMJ Publishing Group Ltd ("BMJ") its licensees and where the relevant Journal is co-owned by BMJ to the co-owners of the Journal, to publish the Work in this journal and any other BMJ products and to exploit all rights, as set out in our licence.

The Submitting Author accepts and understands that any supply made under these terms is made by BMJ to the Submitting Author unless you are acting as an employee on behalf of your employer or a postgraduate student of an affiliated institution which is paying any applicable article publishing charge ("APC") for Open Access articles. Where the Submitting Author wishes to make the Work available on an Open Access basis (and intends to pay the relevant APC), the terms of reuse of such Open Access shall be governed by a Creative Commons licence – details of these licences and which Creative Commons licence will apply to this Work are set out in our licence referred to above.

Other than as permitted in any relevant BMJ Author's Self Archiving Policies, I confirm this Work has not been accepted for publication elsewhere, is not being considered for publication elsewhere and does not duplicate material already published. I confirm all authors consent to publication of this Work and authorise the granting of this licence.

For peer review only

Title page

Title

Prognostic factors and prediction models for hospitalisation and all-cause mortality in adults presenting to primary care with a lower respiratory tract infection: a systematic review

Authors

Merijn H. Rijk[1], MD; Tamara N. Platteel[1], MD PhD; Teun M. C. van den Berg[1], MD; Geert-Jan Geersing[1], MD PhD; Paul Little[2], MD PhD; Frans H. Rutten[1], MD PhD; Maarten van Smeden[3]*, PhD; Roderick P. Venekamp[1]*, MD PhD

Affiliations

[1] Department of General Practice & Nursing Science, Julius Center for Health Sciences and Primary Care, University Medical Center Utrecht, Utrecht University, Utrecht, the Netherlands

[2] Primary Care Research Centre, Primary Care Population Sciences and Medical Education Unit, University of Southampton, Southampton, United Kingdom

[3] Department of Epidemiology & Health Economics, Julius Center for Health Sciences and Primary Care, University Medical Center Utrecht, Utrecht University, Utrecht, the Netherlands

* MvS and RPV contributed equally to this work.

Corresponding author

Merijn H. Rijk, MD

For peer review only
For peer review only - http://bmjopen.bmj.com/site/about/guidelines.xhtml

Department of General Practice & Nursing Science, Julius Center for Health Sciences and Primary Care, University Medical Center Utrecht, Utrecht University, Utrecht, the Netherlands

Universiteitsweg 100, 3584 CG Utrecht

Phone: 0031 88 75 681 81

Email: m.h.rijk@umcutrecht.nl

For peer review only

Prior presentation(s)

None

Word count

Abstract: 300

Main text: 3435

Number of tables, figures, and appendices

Tables: 2

Figures: 3

Appendices: 9

For peer review only - http://bmjopen.bmj.com/site/about/guidelines.xhtml

Abstract

Objective To identify and synthesize relevant existing prognostic factors (PF) and prediction models (PM) for hospitalisation and all-cause mortality within 90 days in primary care patients with acute lower respiratory tract infections (LRTI).

Design Systematic review.

Methods Systematic searches of MEDLINE, Embase, and the Cochrane Library were performed. All PF and PM studies on risk of hospitalisation or all-cause mortality within 90 days in adult primary care LRTI patients were included. Risk of bias was assessed using the QUIPS and PROBAST tools for PF and PM studies, respectively. Results of included PF and PM studies were descriptively summarised.

Results Of 2,799 unique records identified, 16 were included: nine PF studies, six PM studies, and one combination of both. Risk of bias was judged high for all studies, mainly due to limitations in the analysis domain. Based on reported multivariable associations in PF studies, increasing age, sex, current smoking, diabetes, a history of stroke, cancer, or heart failure, previous hospitalisation, influenza vaccination (negative association), current use of systemic corticosteroids, recent antibiotic use, respiratory rate ≥25/minute, and diagnosis of pneumonia were identified as most promising candidate predictors. One newly developed PM was externally validated (c statistic 0.74, 95% confidence interval [CI] 0.71 to 0.78) whereas the previously hospital-derived CRB-65 was externally validated in primary care in five studies (c statistic ranging from 0.72 [95% CI 0.63 to 0.81] to 0.79 [95% CI 0.65 to 0.92]). None of the PM studies reported measures of model calibration.

Conclusions Implementation of existing models for individualised risk prediction of 90-day hospitalisation or mortality in primary care LRTI patients in everyday practice is hampered by incomplete assessment of model performance. The identified candidate predictors provide useful information for clinicians and warrant consideration when developing or updating PMs using state-of-the-art development and validation techniques.

PROSPERO registration number CRD42022341233

For peer review only - http://bmjopen.bmj.com/site/about/guidelines.xhtml

Strengths and limitations of this study

- Both prognostic factors and prediction models were covered in this comprehensive systematic review.
- The systematic search syntax included a validated prognostic search string to identify all relevant prognostic literature.
- Prevailing guidelines were followed for data extraction (CHARMS-PF and CHARMS), quality assessment (QUIPS, PROBAST, and GRADE) and reporting (PRISMA).
- Substantial between-study heterogeneity hampered pooling of the results in meta-analysis.

For peer review only

Keywords

Lower respiratory tract infections; primary care; general practice; prognosis; prognostic factor; prediction model; systematic review

Abbreviations

LRTI	Lower respiratory tract infections
CAP	Community-acquired pneumonia
GP	General practitioner
PSI	Pneumonia severity index
PF	Prognostic factor
PM	Prediction model
CHARMS	Checklist for Critical Appraisal and Data Extraction for Systematic Reviews of Prediction Modelling Studies
QUIPS	Quality in Prognostic Studies
PROBAST	Prediction Model Risk Of Bias Assessment Tool
GRADE	Grading of Recommendations, Assessment, Development, and Evaluations
CI	Confidence interval
O/E	Observed over expected
COVID-19	Coronavirus disease 2019
NICE	National Institute for Health and Care Excellence
ATS	American Thoracic Society
IDSA	Infectious Diseases Society of America

For peer review only

Introduction

Acute lower respiratory tract infections (LRTI) are common in primary care.(1,2) Uncomplicated LRTI episodes generally have a favourable natural course in otherwise healthy adults, and antibiotic treatment with amoxicillin confers only little benefit in terms of earlier symptom resolution, both overall and in higher-risk subgroups of patients.(3,4)(5) The clinical spectrum of LRTI patients, however, is heterogenous in terms of patient and disease specific characteristics, and thus the risk of hospitalisation and death varies substantially. For example, observational evidence suggests that adverse outcomes such as hospitalisation and death occur in fewer than 1% of patients with uncomplicated LRTI(5), whereas this risk is far more pronounced among those with community-acquired pneumonia (CAP).(6,7) However, diagnosing CAP in a primary care setting is notoriously challenging(8,9), leaving general practitioners (GP) with uncertainties how to identify higher-risk groups among those presenting with LRTI symptoms.

Nevertheless, identification of LRTI patients at highest risk of complications is pivotal as this might help GPs identify those in whom close follow-up or early (antibiotic) treatment is warranted. Currently, several scores exist to assist physicians in estimating risk of adverse outcomes in LRTI patients, such as the Pneumonia Severity Index (PSI) and CURB-65 (a prediction rule including confusion, blood urea nitrogen, respiratory rate, blood pressure, and age ≥65 years).(10,11) However, these prediction rules have been developed in secondary care and the inclusion of laboratory and/or radiographic features hampers applicability to outpatients. A modified version of the CURB-65 (CRB-65, not including blood urea nitrogen) has been proposed as primary care alternative, but its development in hospitalised patients raises uncertainty about its performance in outpatients.(10) An overview of existing primary care-specific prognostic factors (PF) and prediction models (PM) for adverse outcomes in LRTI patients could provide insight on (i) existing PFs or PMs suitable for use in clinical practice and (ii) relevant PFs to include when developing a new or updating an existing model. We therefore aimed to synthesize existing knowledge on PFs and PMs for hospitalisation and mortality within 90 days in adult patients presenting to primary care with LRTI.

For peer review only - http://bmjopen.bmj.com/site/about/guidelines.xhtml

Methods

The protocol for this review was registered in PROSPERO (CRD42022341233) and the review was reported according to the PRISMA reporting guideline for systematic reviews.(12)

Searches and study selection

The electronic databases of MEDLINE, Embase, and the Cochrane Library were searched on 27 June 2022 for eligible studies. Keywords for 'respiratory tract infection', 'hospitalisation', 'death', 'adults' and 'primary care' were combined with a validated search string to identify relevant prognostic studies (Table S1).(13) Eligibility criteria were based on PICOTS guidance; we included all studies on adults with LRTI (patients) in which prognostic factors or prediction models (index) for 90-day hospitalisation or all-cause mortality (outcome) were assessed on the day of diagnosis (timing) in primary care (setting).(14,15) Non-English articles or those without full-text availability were excluded.

After de-duplication, two review authors (MHR, TMCB) independently reviewed titles/abstracts of unique records retrieved from the electronic databases against the eligibility criteria. Next, the same authors independently reviewed the full text of potentially relevant articles for eligibility. Any disagreements were resolved by discussion and, where necessary, by consulting a third reviewer (TNP). To increase the yield of potentially eligible articles, reference lists of the included articles were reviewed for eligibility. Studies assessing the prognostic value of PFs, developing a new PM, or externally validating a previously developed PM were eligible for inclusion.

Data collection

Data from eligible PF and PM studies were extracted by MHR using standardised forms based on the Checklist for Critical Appraisal and Data Extraction for Systematic Reviews of PF studies (CHARMS-PF) and PM studies (CHARMS), respectively.(14,16) Main outcomes of interest were hospitalisation or death within 90 days after initial LRTI consultation. For PF studies we

For peer review only - http://bmjopen.bmj.com/site/about/guidelines.xhtml

extracted odds ratios, hazard ratios, or risk ratios with accompanying 95% confidence intervals (CI) from both univariable and multivariable analyses. For those studies that did not report such estimates, these associations were calculated from crude data using Rothman's Episheet version 15 November 2021 wherever possible. For PM studies, model performance estimates in terms of discrimination (c statistic with accompanying 95% CI) and calibration (intercept and slope) were extracted. If calibration measures were absent, the total observed over expected (O/E) ratio with accompanying 95% CI of patients with the outcome was calculated wherever possible as a proxy of overall model calibration.(15) To this end, we used data on the distribution of subjects and outcomes across different PM risk strata and the corresponding predicted risks of the strata as reported in the original model development study. These calculations were performed in R version 4.2.2 using the 'metamisc' package.

Other data extraction items included study population, candidate predictors, sample size, (handling of) missing data, analysis (modelling method and/or model development and validation method), and interpretation of results. Where needed, corresponding authors of individual studies were contacted to request additional data.

Assessment of risk of bias and applicability

Risk of bias assessment was performed by two authors (MHR, TNP) independently using the Quality in Prognostic Studies tool (QUIPS, assessing study participation, study attrition, PF measurement, outcome measurement, and statistical analysis) and the Prediction Model Risk Of Bias Assessment Tool (PROBAST, covering the domains of participants, predictors, outcome, and analysis) for PF and PM studies, respectively.(17,18) We excluded the 'study confounding' item from the QUIPS, since confounding is not applicable to prognostic research. Applicability of included articles was assessed based on the PICOTS criteria.

Synthesis methods

For peer review only - http://bmjopen.bmj.com/site/about/guidelines.xhtml

Due to substantial heterogeneity in domain, predictor, and outcome definition, combining PF effect estimates or PM performance measures in meta-analyses was considered inappropriate and results were therefore descriptively summarised, stratified according to diagnosis (i.e. LRTI versus pneumonia). Forest plots were created to visually summarise (i) univariable and multivariable effect estimates of PFs, and (ii) discrimination and calibration performance measures of PMs. PFs were categorised into either demographics, patient history, healthcare use, medication use, signs and symptoms, laboratory tests, or diagnosis. Promising candidate predictors were identified based on reported results of multivariable analysis in PF studies. For each promising PF and PM, we applied the grading of recommendations, assessment, development, and evaluations (GRADE) framework using prognostic research-specific guidance to rate the quality of evidence.(19,20)

Patient and public involvement

There was no patient or public involvement in this study.

For peer review only

Results

Study selection

The search strategy yielded a total of 3,191 records. Removing duplicates left 2,799 unique records (Figure 1). After screening title and abstract, 34 studies remained for full-text review. Of these, 19 were excluded (Figure 1). One additional publication was retrieved from screening reference lists(21), leaving 16 publications for inclusion: nine PF studies(21–29), six PM studies(30–35), and one combination of PM and PF(36).

Study characteristics

The PF studies were conducted between 1997-2021 of which most (7/10) used a retrospective cohort design (Table 1). The study population in terms of age (minimum age ranging from 1-80 years) and LRTI definition (ranging from suspected LRTI to diagnosis of CAP), as well as the hospitalisation rate (from 0.7% in LRTI patients to 76.5% CAP patients) varied substantially across studies. Mortality was used as outcome in four studies, hospitalisation in three studies, and a composite of both in one study. Two studies also included deterioration of existing illness and late-onset pneumonia in addition to hospitalisation and mortality in their outcome(23,36), while the timing of outcome assessment was unknown in one study(29).

The PM studies were conducted between 2000-2013 and the majority (4/7) used a prospective cohort design. In total, nine different PMs could be included in the synthesis: CRB, CRB-65, CURB, CURB-65, PSI, a model developed by Bont et al., RISSC85, CORB-75, and a model developed by Moore et al. (Table 2). Four studies developed a new prediction model(30,32,35,36) and five externally validated previously developed models(31–35), of which the CRB-65 was validated in all five studies. Clinical heterogeneity was mostly attributed to age (minimum age ranging from 16-65 years) and LRTI definition (ranging from suspected LRTI to radiographically confirmed CAP). Hospitalisation rates varied between 0.5% in LRTI patients and 86.9% in CAP patients. All PM studies used binary outcomes. Mortality was used as outcome in three studies, hospitalisation in one study, and a composite of both in one study.

For peer review only - http://bmjopen.bmj.com/site/about/guidelines.xhtml

One study also included re-consultation with new or worsened symptoms in addition to hospitalisation in their outcome(35), while another study included late-onset pneumonia in addition to hospitalisation and mortality.(36)

Risk of bias assessment

Overall risk of bias was judged high for all studies (Figures S2 and S3). For PF studies, this was mostly due to issues in the statistical analysis domains. For instance, five studies selected predictors for multivariable analysis based on univariable associations(21–25), and two included multiple episodes per patient but did not perform recurrent event analysis.(25,27) Furthermore, in some studies an adequate definition of LRTI or PFs was lacking, and methods to account for missing data were mostly judged inadequate or not described.

Similarly, risk of bias due to the issues in the analysis domain was judged high for all PM studies. In all model development studies continuous predictors were dichotomised, and handling of missing data was judged inadequate or not reported. None of the PM studies reported any evaluation of model calibration.

Prognostic factors

Three PF studies did not report estimates of univariable associations of PFs, and these could also not be calculated from the reported data.(24,27,28) The remaining PF studies reported univariable analysis of 69 PFs, of which sex, age, and history of pulmonary disease were analysed most frequently (Figure S4). If reported in the primary study, absolute risks according to the presence and absence of individual PFs per study are presented in Table S5.

Estimates of multivariable associations were reported for 74 PFs (Figure S6), of which 21 were analysed in at least two studies. The number of covariates per multivariable analysis ranged from 3 to 29, and a common set of covariates across studies was lacking. Age was

For peer review only - http://bmjopen.bmj.com/site/about/guidelines.xhtml

analysed in five studies, and all found increasing age to be associated with poor outcome.(23,24,27,28,36) Apart from increasing age, PFs that were identified as promising based on multiple multivariable analyses were sex (significant association in two/five studies), current smoking (three/four studies), diabetes (two/three studies), a history of stroke (two/three studies), cancer (three/three studies), or heart failure (two/two studies), hospitalisation in the previous year (two/two studies), current use of systemic corticosteroids (two/three studies), antibiotic use in the previous month (two/three studies), a respiratory rate ≥25/minute (two/two studies), and diagnosis of pneumonia (two/two studies) (Figure 2). Having received an influenza vaccination in the previous year was the only promising candidate predictor estimated to have a negative association, based on a significant multivariable association in two/three studies. The quality of evidence was judged low for age, hospitalisation in the previous year, and diagnosis of pneumonia and very low for the remaining promising PFs (Table S7).

Prediction models

Several PMs included predictors that were also identified as promising candidate predictors based on PF studies (Table S8). Sex, current smoking, and influenza vaccination were the only promising candidate predictors that were not incorporated in any of the PMs. Predictors that were not identified as promising PFs but were incorporated in PMs include history of lung, renal or liver disease, extent to which complaints interfere with daily activities, sputum or coryza, chest pain, confusion, presence of crackles, heart rate, blood pressure, oxygen saturation, body temperature, blood urea nitrogen, and an overall GP severity assessment score.

Four studies developed a new PM (three for use in LRTI and one for CAP patients), in which the number of events per predictor ranged from 3.3-13.7.(30,34–36) C statistics ranged from 0.63 (95% CI 0.61-0.67) to 0.82 (95% CI 0.75-0.88) after (internal or external) model

For peer review only - http://bmjopen.bmj.com/site/about/guidelines.xhtml

validation (Figure 3). One study performed external validation(30), whereas other studies only conducted internal(-external) validation.(34–36) None reported measures of model calibration, while a total O/E ratio could not be calculated for any model.

All five PM validation studies externally validated the CRB-65.(31–35) C statistics for 30-day mortality in CAP patients (range outcome events: 11 to 80) ranged from 0.72 (95% CI 0.66-0.78) to 0.79 (95% CI 0.65-0.92)(31,32,34) (Figure 3). The c statistic for 28-day re-consultation or hospitalisation in LRTI patients was substantially lower: 0.53 (95% CI 0.51-0.56).(35) None reported measures of model calibration. A total O/E ratio could be calculated for the CRB-65 in four studies, ranging from 0.67 (95% CI 0.26-1.08) to 1.42 (95% CI 1.13-1.71).

Other validated PMs were PSI, CURB-65, CURB, and CRB. C statistics for 28-day re-consultation or hospitalisation in LRTI patients ranged from 0.51 (95% CI 0.50-0.54) to 0.53 (95% CI 0.50-0.55).(35) Calibration measures were not reported and total O/E ratios could not be calculated. C statistics of PSI and CURB-65 for 30-day mortality in CAP patients were 0.73 (95% CI 0.67-0.79) and 0.67 (95% CI 0.61-0.74), respectively.(32) Model calibration measures were not reported, while the total O/E ratios were estimated at 1.46 (95% CI 1.16-1.76) and 1.92 (95% CI 1.53-2.32), respectively. The quality of evidence was judged low for CRB-65 and very low for all other PMs included in this systematic review (Table S9).

For peer review only - http://bmjopen.bmj.com/site/about/guidelines.xhtml

Discussion

Summary of main findings

This systematic review summarised evidence on PFs and PMs for hospitalisation and mortality within 90 days in patients presenting to primary care with LRTI. The risk of bias of included studies was judged high, mainly due to issues in the analysis domains. Of all PFs analysed, thirteen were identified as most promising; increasing age, sex, current smoking, diabetes, a history of stroke, cancer, or heart failure, hospitalisation in the previous year, current use of systemic corticosteroids, antibiotic use in the previous month, a respiratory rate ≥25/minute, and diagnosis of pneumonia were associated with poor outcome whereas seasonal influenza vaccination was estimated to have a negative association. Most of these promising candidate predictors have also been incorporated in the identified PMs. The secondary care-derived CRB-65, predicting mortality in CAP patients, and a newly developed PM by Bont et al.(30) predicting hospitalisation and mortality in elderly LRTI patients both showed promising results after external validation. However, none assessed model calibration, leaving the precision of predicted risks unknown.

Comparison with existing literature

During the coronavirus disease 2019 (COVID-19) pandemic, many studies have focused on PFs associated with poor outcomes in COVID-19 patients, and some were similar to those identified in our review of all-cause LRTI patients. A recent, field-wide systematic review and meta-analysis of PFs for adverse outcomes in COVID-19 patients found cancer, insulin use, and smoking – among other factors – to be associated with mortality, and age with both hospitalisation and mortality.(37) Another community-based systematic review of European COVID-19 patients found male sex, heart failure, and diabetes to be associated with hospitalisation, whereas stroke, heart failure, and neurologic disease were associated with increased mortality risk.(38) Although we did not identify studies that assessed the prognostic

For peer review only - http://bmjopen.bmj.com/site/about/guidelines.xhtml

value of specific viral respiratory pathogens such as SARS-CoV-2, respiratory syncytial virus and influenza, it is likely that the specific pathogen is a relevant prognostic factor, and point-of-care tests for respiratory pathogens are increasingly available in the post-pandemic era. The potential usefulness of such point-of-care tests is supported by evidence of patients with a viral LRTI admitted to the emergency room in whom specific viral pathogens were associated with increased risk of hospitalisation.(39)

Some of the identified PMs have been incorporated in LRTI guidelines – mainly those focusing on CAP – as additional tool to aid patient management decisions. The CAP guideline issued by the American Thoracic Society (ATS) and the Infectious Diseases Society of America (IDSA) supports the use of PSI or CURB-65 – in addition to clinical judgement – to determine the appropriate level of care, i.e. inpatient versus outpatient management.(40,41) Although this guideline applies to all levels of health care, the value of the suggested models for GPs is limited since models' laboratory and radiographic features are not routinely available in primary care. The UK National Institute for Health and Care Excellence (NICE) guideline on pneumonia advocates the use of CRB-65; it recommends GPs to consider hospital admission in patients with a score of ≥1.(42) Guidelines vary with regard to the incorporation of individual factors that are associated with poor outcome. The ATS/IDSA guideline and the Dutch primary care guideline both list tachypnoea and confusion as indicators of disease severity, while the Dutch guideline also emphasised the importance of PFs such as age, history of heart failure, and diabetes.(40,43) The NICE guideline does not explicitly highlight individual factors associated with poor outcome in adult patients.(42)

Similar to our findings, a review of the performance of CRB-65 for predicting 30-day mortality among CAP patients in community and hospital settings concluded that this model has been insufficiently validated for use in primary care.(44) Although the population of this review slightly differed from ours by also including patients who visited the emergency department, a similarly low number of outcome events was found. Contrary to the slight overprediction reported in this review, our synthesis of more recent studies suggested

For peer review only

underprediction of risks based on total O/E ratios. However, in absence of proper assessment of model calibration the true precision of predicted risks remains to be elucidated.

Strengths and limitations

By covering both PFs and PMs in a single review, we have provided a comprehensive overview of available prognostic literature of primary care LRTI patients. We prospectively registered our review protocol, conducted a broad search with incorporation of a validated prognostic search string, and thoroughly reviewed citations of eligible studies to assure all relevant literature was included in the synthesis. By including various prognostic study designs, our synthesis provides a comprehensive overview of all prognostic evidence on primary care LRTI patients. Studies assessing the prognostic value of individual factors, developing a new or validating an existing model all provide insight on important PFs that could be used in future prognostic research, whereas studies validating a new or previously developed model such as CRB-65 provide insight on the suitability for its use in clinical practice.

Some limitations should, however, be acknowledged. First, our findings should be interpreted with caution since risk of bias was judged high for all included studies and the quality of the evidence was judged low to very low for the promising PFs and PMs. Second, due to substantial heterogeneity in domain, predictor, and outcome definitions between studies, we refrained from conducting meta-analyses of PF effect estimates or PM performance measures, which makes direct comparison of study results difficult. It is plausible that PF effect estimates for poor outcome vary according to age, diagnosis, and disease severity (a priori risk of event). For example, half of the participants in Hak et al.(23) had either pneumonia or an exacerbation of chronic obstructive pulmonary disease, whereas Moore et al.(36) had fewer than 2% of such patients. Furthermore, one study included patients aged >1 year (median age of included participants: 54 years [interquartile range: 37]) but did not specifically report separate results for age strata.(24) Given the median age, we considered

For peer review only

including this study on prognostic factors in our review justified. Consistency in effect estimates would have further facilitated direct comparison of PFs, but converting the various reported estimates to odds ratios appeared not possible. Albeit our synthesis of PFs provides some useful information for clinicians, it merely informs future research aimed at developing or updating PMs. Third, we excluded most studies on patients with a specific respiratory pathogen (e.g. SARS-CoV-2 or influenza) since these included patients with both upper and lower respiratory tract infections and did not report stratified results for LRTI. As such, we were unable to present any respiratory pathogen-specific results regarding prognostic factors for poor outcome in patients presenting to primary care with LRTI. However, since GPs are typically unaware of the specific pathogen in LRTI patients, such results would have limited applicability to current clinical practice. Lastly, PM building techniques were generally suboptimal which increased risk of bias, and none of the identified PM studies assessed model calibration. In contrast to discrimination, calibration generally receives little attention when assessing PM performance, whereas well discriminating but poorly calibrated models can lead to imprecise, misleading predictions.(45) Total O/E ratios calculations provided some insight into mean model calibration – the most basic level in the hierarchy of calibration assessment – but this is insufficient to provide guidance on the safe use of a PM in clinical practice.(46)

Implications for research and clinical practice

Based on our synthesis, implementation of existing PMs for individualised risk prediction of 90-day hospitalisation or all-cause mortality in primary care LRTI patients in everyday practice is hampered by incomplete assessment of model performance. The identified candidate predictors provide useful information for clinicians about factors associated with poor outcome in primary care LRTI patients and warrant consideration when developing or updating PMs using state-of-the-art development and appropriate validation techniques.

For peer review only - http://bmjopen.bmj.com/site/about/guidelines.xhtml

Declarations

Author contributions

MHR, TNP, MvS, and RPV were involved in the conceptualisation and design of the study. MHR, TMCB, and TNP conducted the study selection procedure. Data of eligible studies was extracted by MHR. MHR, TNP, and MvS performed the risk of bias assessment. MHR wrote the first draft of the manuscript, which was initially reviewed by TNP, MvS, and RVP. MHR, TNP, TMCB, GJG, PL, FHR, MvS, and RVP reviewed and approved the final manuscript.

For peer review only

Ethical approval

This study did not involve human participants and ethical approval was therefore not required.

Funding

This study was funded by ZonMw (grant number 08391052110003). The funder played no role in study design, data collection, analysis and interpretation of data, or the writing of this manuscript.

Competing interests

All authors declare no financial or non-financial competing interests.

Patient and public involvement

There was no patient or public involvement in this study.

Data availability

For peer review only - http://bmjopen.bmj.com/site/about/guidelines.xhtml

Study materials (e.g. template data collection forms, data extracted from included studies) are available from the corresponding author upon reasonable request.

For peer review only

For peer review only - http://bmjopen.bmj.com/site/about/guidelines.xhtml

References

1. Bergmann M, Haasenritter J, Beidatsch D, Schwarm S, Hörner K, Bösner S, et al. Prevalence, aetiologies and prognosis of the symptom cough in primary care: a systematic review and meta-analysis. BMC Fam Pract. 2021;22(1):1–19.

2. Hak E, Rovers MM, Kuyvenhoven MM, Schellevis FG, Verheij TJM. Incidence of GP-diagnosed respiratory tract infections according to age, gender and high-risk co-morbidity: The Second Dutch National Survey of General Practice. Fam Pract. 2006;23(3):291–4.

3. Little P, Stuart B, Moore M, Coenen S, Butler CC, Godycki-Cwirko M, et al. Amoxicillin for acute lower-respiratory-tract infection in primary care when pneumonia is not suspected: A 12-country, randomised, placebo-controlled trial. Lancet Infect Dis. 2013;13(2):123–9.

4. Moore M, Stuart B, Coenen S, Butler CC, Goossens H, Verheij TJM, et al. Amoxicillin for acute lower respiratory tract infection in primary care: Subgroup analysis of potential high-risk groups. British Journal of General Practice. 2014;64(619):75–80.

5. Little P, Stuart B, Smith S, Thompson MJ, Knox K, Van Den Bruel A, et al. Antibiotic prescription strategies and adverse outcome for uncomplicated lower respiratory tract infections: Prospective cough complication cohort (3C) study. BMJ (Online). 2017;357.

6. Welte T, Torres A, Nathwani D. Clinical and economic burden of community-acquired pneumonia among adults in Europe. Thorax. 2012;67(1):71–9.

7. McLaughlin JM, Khan FL, Thoburn EA, Isturiz RE, Swerdlow DL. Rates of hospitalization for community-acquired pneumonia among US adults: A systematic review. Vaccine. 2020;38(4):741–51.

8. Teepe J, Broekhuizen BDL, Loens K, Lammens C, Ieven M, Goossens H, et al. Predicting the presence of bacterial pathogens in the airways of primary care patients with acute cough. CMAJ. 2017;189(2):E50–5.

9. Minnaard MC, De Groot JAH, Hopstaken RM, Schierenberg A, De Wit NJ, Reitsma JB, et al. The added value of C-reactive protein measurement in diagnosing pneumonia in primary care: A meta-analysis of individual patient data. CMAJ. 2017;189(2):E56–63.

10. Lim WS, Van Der Eerden MM, Laing R, Boersma WG, Karalus N, Town GI, et al. Defining community acquired pneumonia severity on presentation to hospital: An international derivation and validation study. Thorax. 2003;58(5):377–82.

11. Fine M, Auble T, Yealy D, Hanusa B, Weissfeld L, Singer D, et al. A prediction rule to identify low-risk patients with community-acquired pneumonia. New England Journal of Medicine. 1997;336(4):243–50.

12. Page MJ, McKenzie JE, Bossuyt PM, Boutron I, Hoffmann TC, Mulrow CD, et al. The PRISMA 2020 statement: An updated guideline for reporting systematic reviews. The BMJ. 2021;372.

13. Geersing GJ, Bouwmeester W, Zuithoff P, Spijker R, Leeflang M, Moons K. Search filters for finding prognostic and diagnostic prediction studies in medline to enhance systematic reviews. PLoS One. 2012;7(2):3–8.

For peer review only - http://bmjopen.bmj.com/site/about/guidelines.xhtml

14. Moons KGM, de Groot JAH, Bouwmeester W, Vergouwe Y, Mallett S, Altman DG, et al. Critical Appraisal and Data Extraction for Systematic Reviews of Prediction Modelling Studies: The CHARMS Checklist. PLoS Med. 2014;11(10).

15. Debray TPA, Damen JAAG, Snell KIE, Ensor J, Hooft L, Reitsma JB, et al. A guide to systematic review and meta-analysis of prediction model performance. BMJ (Online). 2017;356.

16. Riley RD, Moons KGM, Snell KIE, Ensor J, Hooft L, Altman DG, et al. A guide to systematic review and meta-analysis of prognostic factor studies. BMJ (Online). 2019;364.

17. Hayden JA, van der Windt DA, Cartwright JL, Côté P, Bombardier C. Assessing Bias in Studies of Prognostic Factors. Ann Intern Med. 2013;158:280–6.

18. Wolff RF, Moons KGM, Riley RD, Whiting PF, Westwood M, Collins GS, et al. PROBAST: A tool to assess the risk of bias and applicability of prediction model studies. Ann Intern Med. 2019;170(1):51–8.

19. Foroutan F, Guyatt G, Zuk V, Vandvik PO, Alba AC, Mustafa R, et al. GRADE Guidelines 28: Use of GRADE for the assessment of evidence about prognostic factors: rating certainty in identification of groups of patients with different absolute risks. J Clin Epidemiol. 2020 May 1;121:62–70.

20. Brozek JL, Canelo-Aybar C, Akl EA, Bowen JM, Bucher J, Chiu WA, et al. GRADE Guidelines 30: the GRADE approach to assessing the certainty of modeled evidence—An overview in the context of health decision-making. J Clin Epidemiol. 2021 Jan 1;129:138–50.

21. Houston MS, Silverstein MD, Suman VJ. Risk Factors for 30-Day Mortality in Elderly Patients With Lower Respiratory Tract Infection: Community-Based Study. Arch Intern Med. 1997;157:2190–5.

22. Seppä Y, Bloigu A, Honkanen PO, Miettinen L, Syrjälä H. Severity assessment of lower respiratory tract infection in elderly patients in primary care. Arch Intern Med. 2001;161(22):2709–13.

23. Hak E, Bont J, Hoes AW, Verheij TJM. Prognostic factors for serious morbidity and mortality from community-acquired lower respiratory tract infections among the elderly in primary care. Fam Pract. 2005;22(4):375–80.

24. Winchester CC, Macfarlane T V., Thomas M, Price D. Antibiotic prescribing and outcomes of lower respiratory tract infection in UK primary care. Chest. 2009;135(5):1163–72.

25. van de Nadort C, Smeets HM, Bont J, Zuithoff NPA, Hak E, Verheij TJM. Prognosis of primary care patients aged 80 years and older with lower respiratory tract infection. British Journal of General Practice. 2009;59(561):261–5.

26. Myles P, Hubbard R, Gibson J, Pogson Z, Smith C, McKeever T. The impact of statins, ace inhibitors and gastric acid suppressants on pneumonia mortality in a UK general practice population cohort. Pharmacoepidemiol Drug Saf. 2009;18(May):697–703.

27. Millett ERC, De Stavola BL, Quint JK, Smeeth L, Thomas SL. Risk factors for hospital admission in the 28 days following a community-acquired pneumonia diagnosis in older adults, and their contribution to increasing hospitalisation rates over time: A cohort study. BMJ Open. 2015;5(12).

For peer review only

For peer review only - http://bmjopen.bmj.com/site/about/guidelines.xhtml

28. Hamilton F, Arnold D, Payne R. Association of prior lymphopenia with mortality in pneumonia: A cohort study in UK primary care. British Journal of General Practice. 2021;71(703):E148–56.

29. Martínez-Redondo J, Comas C, Salud JP, Crespo-Pons M, García-Serrano C, Bravo MO, et al. The risk of hospitalization in COVID-19 patients can be predicted by lung ultrasound in primary care. Int J Environ Res Public Health. 2021;18(11).

30. Bont J, Hak E, Hoes AW, Schipper M, Schellevis FG, Verheij TJM. A prediction rule for elderly primary-care patients with lower respiratory tract infections. European Respiratory Journal. 2007;29(5):969–75.

31. Bont J, Hak E, Hoes AW, Macfarlane JT, Verheij TJ. Predicting death in elderly patients with community-acquired pneumonia: a prospective validation study reevaluating the CRB-65 severity assessment tool. Arch Intern Med. 2008;168(13):1465–8.

32. Ochoa-Gondar O, Vila-Corcoles A, Rodriguez-Blanco T, Ramos F, De Diego C, Salsench E, et al. Comparison of three predictive rules for assessing severity in elderly patients with CAP. Int J Clin Pract. 2011;65(11):1165–72.

33. Francis NA, Cals JW, Butler CC, Hood K, Verheij T, Little P, et al. Severity assessment for lower respiratory tract infections: Potential use and validity of the CRB-65 in primary care. Primary Care Respiratory Journal. 2012;21(1):65–70.

34. Ochoa-Gondar O, Vila-Corcoles A, Rodriguez-Blanco T, Hospital I, Salsench E, Ansa X, et al. Validation of the CORB75 (confusion, oxygen saturation, respiratory rate, blood pressure, and age ≥75 years) as a simpler pneumonia severity rule. Infection. 2013;42(2):371–8.

35. Bruyndonckx R, Hens N, Verheij TJM, Aerts M, Ieven M, Butler CC, et al. Development of a prediction tool for patients presenting with acute cough in primary care: A prognostic study spanning six European countries. British Journal of General Practice. 2018;68(670):e342–50.

36. Moore M, Stuart B, Lown M, Van Den Bruel A, Smith S, Knox K, et al. Predictors of adverse outcomes in uncomplicated lower respiratory tract infections. Ann Fam Med. 2019;17(3):231–8.

37. Bellou V, Tzoulaki I, van Smeden M, Moons KGM, Evangelou E, Belbasis L. Prognostic factors for adverse outcomes in patients with COVID-19: A field-wide systematic review and meta-analysis. Vol. 59, European Respiratory Journal. European Respiratory Society; 2022.

38. Vardavas CI, Mathioudakis AG, Nikitara K, Stamatelopoulos K, Georgiopoulos G, Phalkey R, et al. Prognostic factors for mortality, intensive care unit and hospital admission due to SARS-CoV-2: a systematic review and metaanalysis of cohort studies in Europe. Vol. 31, European Respiratory Review. European Respiratory Society; 2022.

39. Lemarie B, Boussaid G, Gault E, Prigent H, Beaune S, Moreau F, et al. Predictors of Hospitalization and Superinfection in Viral Respiratory Tract Infections Between Influenza and Paramyxoviruses: The SUPERFLUOUS Study. Journal of Infectious Diseases. 2022 Sep 15;226(6):1027–35.

40. Metlay JP, Waterer GW, Long AC, Anzueto A, Brozek J, Crothers K, et al. Diagnosis and treatment of adults with community-acquired pneumonia. Am J Respir Crit Care Med. 2019 Oct 1;200(7):E45–67.

For peer review only - http://bmjopen.bmj.com/site/about/guidelines.xhtml

41. Armstrong CAFP. Community-Acquired Pneumonia: Updated Recommendations from the ATS and IDSA. Am Fam Physician. 2020;102(2):121–4.

42. National Institute for Health and Care Excellence (NICE). Pneumonia in adults: diagnosis and management (Clinical guideline 191) [Internet]. 2014. Available from: www.nice.org.uk/guidance/cg191

43. Verheij T, Hopstaken R, Prins J, Salomé P, Bindels P, Ponsioen B, et al. Nederlands Huisartsen Genootschap. 2020 [cited 2022 May 25]. NHG-Standaard Acuut hoesten. Available from: https://richtlijnen.nhg.org/standaarden/acuut-hoesten#volledige-tekst

44. McNally M, Curtain J, O'Brien KK, Dimitrov BD, Fahey T. Validity of British Thoracic Society guidance (the CRB-65 rule) for predicting the severity of pneumonia in general practice: Systematic review and meta-analysis. British Journal of General Practice. 2010;60(579):423–33.

45. van Calster B, McLernon DJ, van Smeden M, Wynants L, Steyerberg EW, Bossuyt P, et al. Calibration: The Achilles heel of predictive analytics. BMC Med. 2019 Dec 16;17(1).

46. van Calster B, Nieboer D, Vergouwe Y, de Cock B, Pencina MJ, Steyerberg EW. A calibration hierarchy for risk models was defined: From utopia to empirical data. J Clin Epidemiol. 2016 Jun 1;74:167–76.

For peer review only

Tables

Table 1. Study characteristics of included prognostic factor studies

Study	*Source of data*	*Study population*	*Included episodes*	*Age, mean (SD) or median (IQR)*	*Outcome [a]*	*Hospitalisation, n (%)*	*Mortality, n (%)*	*Total outcome events, n (%)*
**Houston, 1997**	Community-based cohort, retrospective	Elderly (≥65 years) LRTI patients	413	NR	30-day all-cause mortality	NR	44 (10.7)	44 (10.7)
**Seppä, 2001**	Primary care cohort, prospective	Elderly (≥65 years) patients with LRTI suggestive of pneumonia	950	Survival: 76 (IQR 71-81), death: 80 (IQR 73-86)	LRTI-related mortality within 30 days	NR	38 (4.0)	38 (4.0)
**Hak, 2005**	Primary care cohort, retrospective	Patients ≥60 years with an LRTI	455	75 (SD 8.6)	Composite of: i) Dysregulation of diabetes, stroke, heart failure, MI, ii) hospitalisation, iii) all-cause mortality, all within 30 days	21 (4.6)	24 (5.3)	65 (14.3)
**Winchester, 2009**	Primary care cohort, retrospective	Patients ≥1 year with an LRTI	151,088	54 (IQR 37)	Respiratory infection-related admission or mortality within 3 months	1,147 (0.8)	2,126 (1.4)	NR
**Van de Nadort, 2009**	Primary care cohort, retrospective	Elderly (≥80 years) LRTI patients	860	85.2 (no SD)	Hospitalisation or death within 30 days	NR	51 (5.9)	109 (12.7)
**Myles, 2009**	Primary care cohort, retrospective	Patients ≥40 years with an LRTI	3,681	NR	All-cause mortality within 30 days	NR	905 (24.6)	905 (24.6)
**Millett, 2015**	Primary care cohort, retrospective	Elderly (≥65 years) CAP patients	43,576	81 (IQR 75-87)	Hospital admission within 28 days	33,321 (76.5)	NR	33,321 (76.5)

For peer review only - http://bmjopen.bmj.com/site/about/guidelines.xhtml

**Moore, 2019**	Primary care cohort, prospective	Patients ≥16 years with acute cough attributed to LRTI	28,846	NR	LRTI-related hospitalisation, mortality or late-onset pneumonia within 2-30 days	196 (0.7)	29 (0.1)	325 (1.1)
**Hamilton, 2021**	Primary care cohort, retrospective	Patients ≥50 years with pneumonia and history of lymphocyte test	28,556	76 (IQR 66-85)	28-day mortality	NR	2,544 (8.9)	2,544 (8.9)
**Martínez-Redondo, 2021**	Primary care cohort, prospective	Adults (≥18 years) with a COVID-19 pneumonia	161	53.0 (no SD)	Hospital admission, no time window reported	37 (23.0)	NR	37 (23.0)

Abbreviations: LRTI, lower respiratory tract infection; CAP, community-acquired pneumonia; COVID-19, coronavirus disease 2019; SD, standard deviation; IQR, interquartile range; NR, not reported; MI, myocardial infarction.

[a] Time window of outcome is defined as days from first consultation within LRTI episode.

For peer review only

For peer review only - http://bmjopen.bmj.com/site/about/guidelines.xhtml

Table 2. Study characteristics of included prediction model studies

Study	*Type of study*	*Model(s)*	*Source of data*	*Study population*	*Age, mean (SD)*	*Outcome [a]*	*Total outcome events, n (%)*	*Method of validation*
**Bont, 2007**	D+V	New	Primary care cohort, retrospective	Elderly (≥65) LRTI patients	75.5 (no SD)	Hospitalisation or all-cause mortality within 30 days	D: 274 (8.7), V: 178 (7.2)	Internal (random split-sample) and external
**Bont, 2008**	V	CRB-65	Primary care cohort, prospective	Elderly (≥65) CAP patients	77.3 (no SD)	Mortality within 30 days	11 (3.5)	External
**Ochoa-Gondar, 2011**	V	PSI, CURB-65, CRB-65	Population-based cohort, retrospective	Elderly (≥65) patients with radiographically confirmed CAP	77.4 (7.6)	Mortality within 30 days	80 (13.6)	External
**Francis, 2012**	V	CRB-65	Primary care cohort, prospective	Adults (≥18) presenting with acute cough or suspected LRTI	49.3 (16.5)	Hospitalisation within 28 days	10 (2.9)	External
**Ochoa-Gondar, 2013**	D+V	CORB-75 (new), CRB-65	Population-based cohort, retrospective	Elderly (≥65) patients with radiographically confirmed CAP	78.1 (7.7)	Mortality within 30 days	D: 46 (13.1), V: 34 (13.1)	Internal-external (temporal, CORB-75), external (CRB-65)
**Bruyndonckx, 2018**	D (RISCC85) + V (all models)	RISCC85 (new), PSI (stage I), CRB, CRB-65, CURB, CURB-65	Primary care cohort, prospective	Adults (≥18) with symptoms suggestive of LRTI	50 (17)	Re-consultation with new/worsened symptoms or hospitalisation within 28 days	521 (20.0)	Internal (random split sample, RISCC85), external (other models)
**Moore, 2019**	D+V	New	Primary care cohort, prospective	Patients ≥16 years with acute cough attributed to LRTI	NR	Hospitalisation, mortality, or clinical diagnosis of pneumonia within 2-30 days	325 (1.1)	Internal (bootstrapping)

For peer review only - http://bmjopen.bmj.com/site/about/guidelines.xhtml

[a] Time window of outcome is defined as days from first consultation.

Abbreviations: D, development; V, validation; LRTI, lower respiratory tract infection; CAP, community acquired pneumonia; SD, standard deviation; NR, not reported.

For peer review only

Figure captions and legends

Figure 1. Flow of articles through study

Legend:

* Systematic reviews have been screened for eligible primary studies

Figure 2. Overview of most promising candidate predictors for hospitalisation or mortality within 90 days based on multivariable analysis.

Legend:

- Blue coloured points: studies on LRTI patients

- Orange coloured points: studies on pneumonia patients

Quality of evidence: as judged based on the GRADE framework.

* Composite outcome also includes dysregulation of diabetes, stroke, heart failure, MI.

** Composite outcome also includes late onset pneumonia.

Abbreviations: LRTI, lower respiratory tract infection; CAP, community-acquired pneumonia; H, hospitalisation; M, mortality; OR, odds ratio; HR, hazard ratio; RR, risk ratio; min, minute.

Figure 3. Overview of discrimination and calibration performance measures of the included prediction models

Legend:

For peer review only - http://bmjopen.bmj.com/site/about/guidelines.xhtml

A. Performance of prediction models evaluated in LRTI patients. B. Performance of prediction models evaluated in community-acquired pneumonia patients.

Abbreviations: LRTI, lower respiratory tract infection; CAP, community-acquired pneumonia; H, hospitalisation; M, mortality; rC, reconsultation; LoP, late onset pneumonia; E, external; IE, internalexternal; I, internal; CI, confidence interval; O/E, observed over expected.

For peer review only

For peer review only - http://bmjopen.bmj.com/site/about/guidelines.xhtml

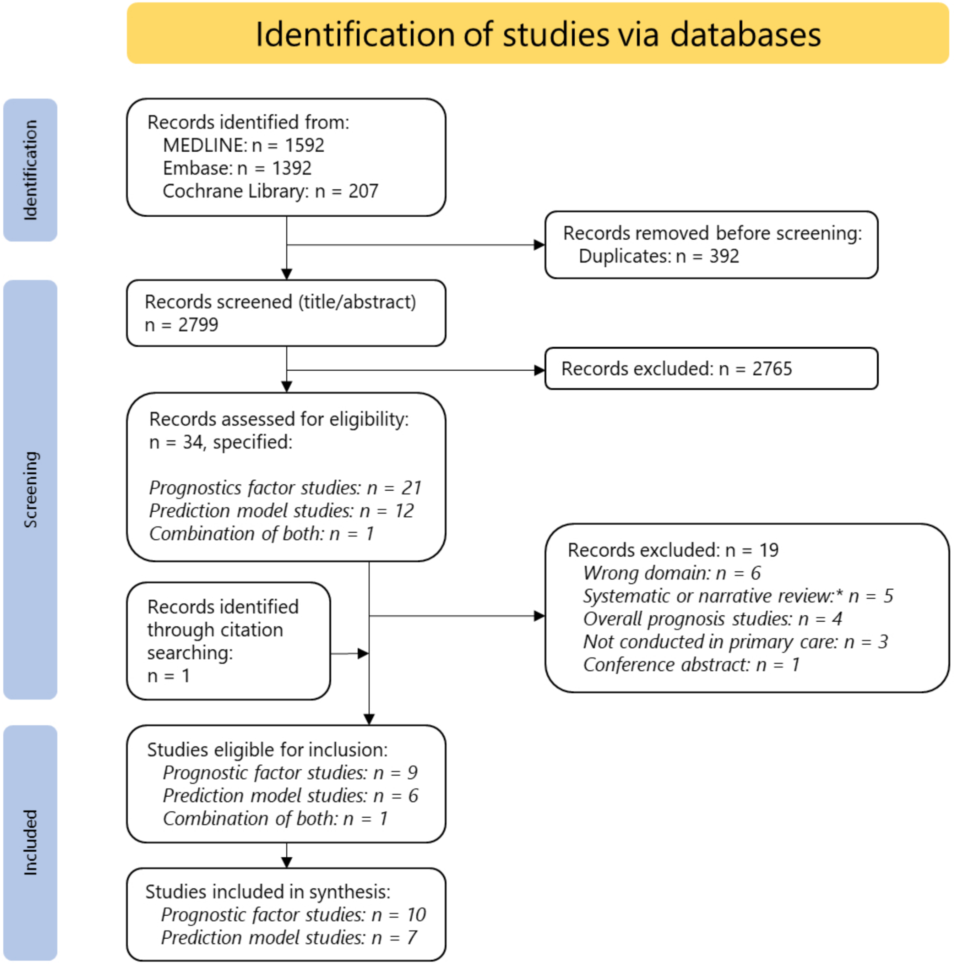

Caption: Figure 1. Flow of articles through study

Legend: * Systematic reviews have been screened for eligible primary studies

184x182mm (150 x 150 DPI)

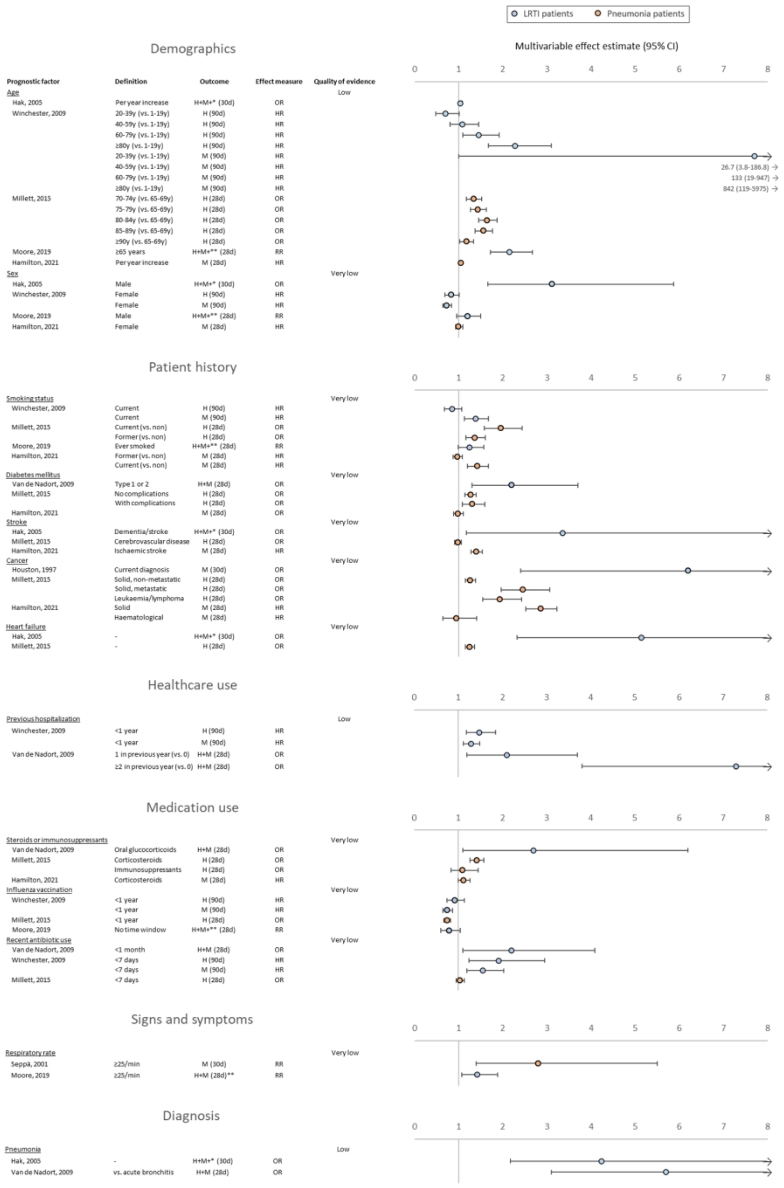

Figure 2. Overview of most promising candidate predictors for hospitalisation or mortality within 90 days based on multivariable analysis.

Legend:
- Blue coloured points: studies on LRTI patients
- Orange coloured points: studies on pneumonia patients

Quality of evidence: as judged based on the GRADE framework.
* Composite outcome also includes dysregulation of diabetes, stroke, heart failure, MI.
** Composite outcome also includes late onset pneumonia.
Abbreviations: LRTI, lower respiratory tract infection; CAP, community-acquired pneumonia; H, hospitalisation; M, mortality; OR, odds ratio; HR, hazard ratio; RR, risk ratio; min, minute.

133x196mm (330 x 330 DPI)

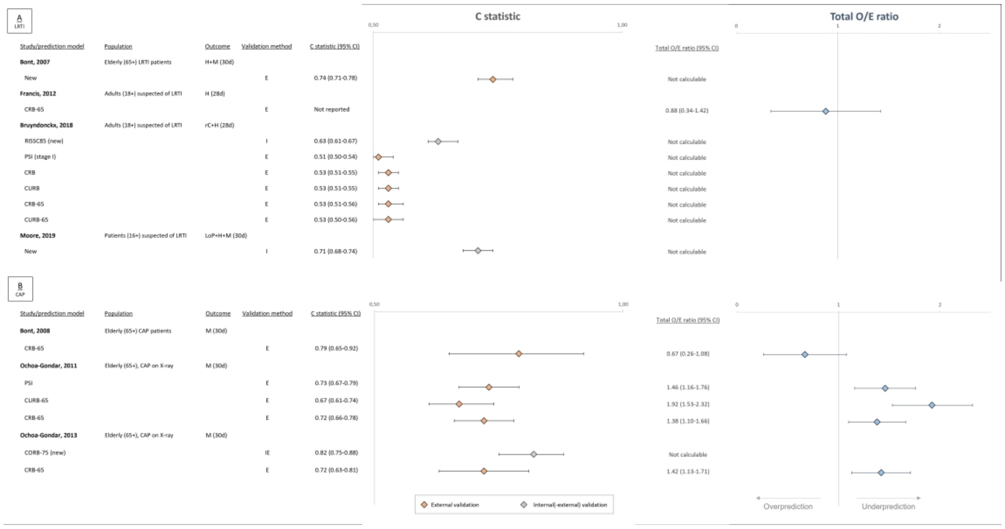

Figure 3. Overview of discrimination and calibration performance measures of the included prediction models

Legend:
A. Performance of prediction models evaluated in LRTI patients. B. Performance of prediction models evaluated in community-acquired pneumonia patients.
Abbreviations: LRTI, lower respiratory tract infection; CAP, community-acquired pneumonia; H, hospitalisation; M, mortality; rC, reconsultation; LoP, late onset pneumonia; E, external; IE, internal-external; I, internal; CI, confidence interval; O/E, observed over expected.

557x290mm (330 x 330 DPI)

Supplementary data

Table S1: search strategy

Search date: 27 June 2022		
**Pubmed**	**Embase**	**The Cochrane Library**
((((("RTI"[Title/Abstract] OR "respiratory tract infection*"[Title/Abstract] OR "respiratory infection*"[Title/Abstract] OR "pneumonia*"[Title/Abstract] OR "bronchitis"[Title/Abstract] OR "lung infection*"[Title/Abstract] OR "chest infection*"[Title/Abstract]) OR ("Respiratory Tract Infections"[Mesh])) AND (((Validat* OR Predict*[Title] OR Rule*) OR (Predict* AND (Outcome* OR Risk* OR Model*)) OR ((History OR Variable* OR Criteria OR Scor* OR Characteristic* OR Finding* OR Factor*) AND (Predict* OR Model* OR Decision* OR Identif* OR Prognos*)) OR (Decision* AND (Model* OR Clinical* OR Logistic Models)) OR (Prognostic AND (History OR Variable* OR Criteria OR Scor* OR Characteristic* OR Finding* OR Factor* OR Model*))) OR (‘‘Stratification’’ OR ‘‘ROC Curve’’[Mesh] OR ‘‘Discrimination’’ OR ‘‘Discriminate’’ OR ‘‘c-statistic’’ OR ‘‘c statistic’’ OR ‘‘Area under the curve’’ OR ‘‘AUC’’ OR ‘‘Calibration’’ OR ‘‘Indices’’ OR ‘‘Algorithm’’ OR ‘‘Multivariable’’))) AND (("hospital*"[Title/Abstract] OR "complicat*"[Title/Abstract] OR "morbidity"[Title/Abstract] OR "death"[Title/Abstract] OR "mortality"[Title/Abstract] OR	(('rti':ti,ab,kw OR 'respiratory tract infection*':ti,ab,kw OR 'respiratory infection*':ti,ab,kw OR 'pneumonia*':ti,ab,kw OR 'bronchitis':ti,ab,kw OR 'lung infection*':ti,ab,kw OR 'chest infection*':ti,ab,kw) OR ('respiratory tract infection'/exp OR 'respiratory tract inflammation'/exp)) AND ((validat* OR predict*:ti OR rule* OR (predict* AND (outcome* OR risk* OR model*)) OR ((history OR variable* OR criteria OR scor* OR characteristic* OR finding* OR factor*) AND (predict* OR model* OR decision* OR identif* OR prognos*)) OR (decision* AND (model* OR clinical* OR logistic) AND models) OR (prognostic AND (history OR variable* OR criteria OR scor* OR characteristic* OR finding* OR factor* OR model*))) OR ('stratification' OR 'receiver operating characteristic'/exp OR 'discrimination' OR 'discriminate' OR 'c-statistic' OR 'c statistic' OR 'area under the curve' OR 'auc' OR 'calibration' OR 'indices' OR 'algorithm' OR 'multivariable')) AND (('hospital*':ti,ab,kw OR 'complicat*':ti,ab,kw OR 'morbidity':ti,ab,kw OR 'death':ti,ab,kw OR 'mortality':ti,ab,kw OR 'surviv*':ti,ab,kw) OR ('hospitalization'/exp OR 'hospital admission'/exp OR	(RTI OR respiratory tract infection OR respiratory infection OR pneumonia OR bronchitis OR lung infection OR chest infection) AND (hospitalisation OR hospitalization OR hospital OR complication OR complicated OR morbidity OR death OR mortality OR survival OR survive) AND (Primary care OR general practice OR general practitioner OR family practice OR GP) in Title Abstract Keyword

For peer review only

"surviv*"[Title/Abstract]) OR ("Mortality"[Mesh] OR "Hospitalization"[Mesh] OR "Death"[Mesh]))) AND (("Primary care"[Title/Abstract] OR "general practi*"[Title/Abstract] OR "family practice"[Title/Abstract] OR "GP"[Title/Abstract]) OR ("Primary Health Care"[Mesh] OR "Family Practice"[Mesh] OR "General Practice"[Mesh]))) NOT (("child*"[Title/Abstract] OR "pediatric*"[Title/Abstract] OR "paediatric*"[Title/Abstract] OR "infan*"[Title/Abstract] OR "adolescen*"[Title/Abstract] OR "young*"[Title/Abstract] OR "newborn*"[Title/Abstract]) OR ("Infant"[Mesh] OR "Child"[Mesh] OR "Adolescent"[Mesh] OR "Pediatrics"[Mesh] OR "Young Adult"[Mesh]))	'death'/exp OR 'mortality'/exp)) AND (('primary care':ti,ab,kw OR 'general practi*':ti,ab,kw OR 'family practice':ti,ab,kw OR 'gp':ti,ab,kw) OR ('primary medical care'/exp OR 'primary health care'/exp OR 'general practice'/exp)) NOT (('child*':ti,ab,kw OR 'pediatric*':ti,ab,kw OR 'paediatric*':ti,ab,kw OR 'infan*':ti,ab,kw OR 'adolescen*':ti,ab,kw OR 'young*':ti,ab,kw OR 'newborn*':ti,ab,kw) OR ('child'/exp OR 'adolescent'/exp OR 'pediatrics'/exp))	
Filters: none<br>Language restrictions: none<br>Publication date restrictions: none	Filters: none<br>Language restrictions: none<br>Publication date restrictions: none	Filters: none<br>Language restrictions: none<br>Publication date restrictions: none
Records identified: 1592	Records identified: 1392	Records identified: 207

For peer review only

Figure S2: Overall assessment of risk of bias and applicability of included studies

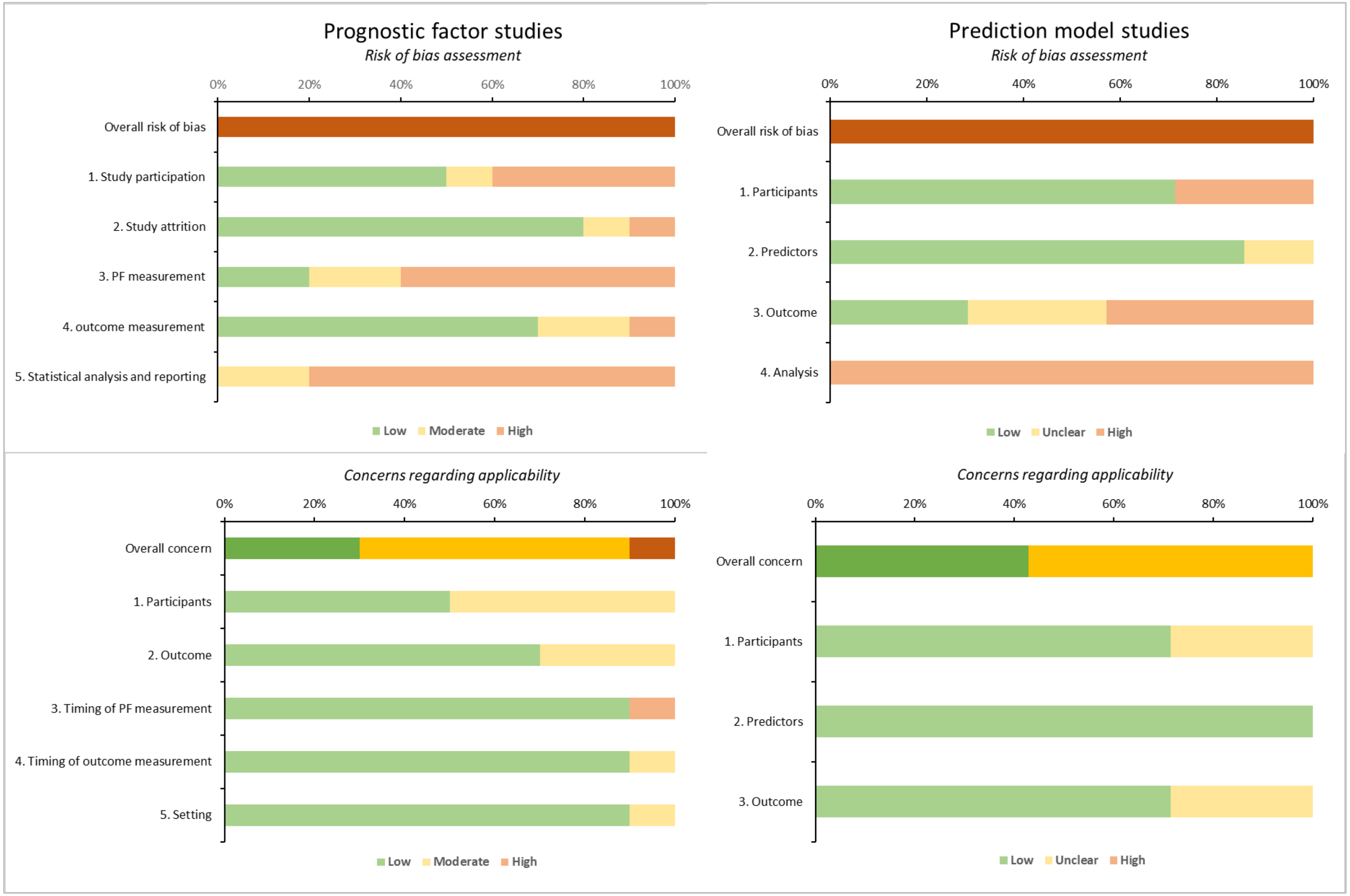

Figure S3: Risk of bias assessment and applicability per individual included study

QUIPS assessment of prognostic factor studies

Study	RoB					Applicability					Overall	
	Study participation	Study attrition	PF measurement	Outcome measurement	Statistical analysis and reporting	Participants	Outcome	Timing of PF measurement	Timing of outcome measurement	Setting	RoB	Applicability
Houston, 1997	±	+	-	+	-	+	+	+	+	±	-	±
Seppä, 2001	-	±	-	+	-	+	+	+	+	+	-	+
Hak, 2005	+	+	±	+	-	+	±	+	+	+	-	±
Myles, 2009	-	+	+	±	-	+	+	+	+	+	-	+
van de Nadort, 2009	+	+	+	±	-	±	+	+	+	+	-	±
Winchester, 2009	+	+	-	+	-	±	±	+	+	+	-	±
Millett, 2015	+	+	±	+	-	+	+	+	+	+	-	+
Moore, 2019	-	+	-	+	±	±	±	+	+	+	-	±
Hamilton, 2021	+	-	-	+	±	±	+	+	+	+	-	±
Martínez-Redondo, 2021	-	+	-	-	-	±	+	-	±	+	-	-

PROBAST assessment of prediction model studies

Study	RoB				Applicability			Overall	
	Participants	Predictors	Outcome	Analysis	Participants	Predictors	Outcome	RoB	Applicability
Bont, 2007	+	+	-	-	+	+	+	-	+
Bont, 2008	-	+	?	-	+	+	+	-	+
Ochoa-Gondar, 2011	+	?	?	-	?	+	+	-	?
Francis, 2012	-	+	-	-	+	+	+	-	+
Ochoa-Gondar, 2013	+	+	+	-	?	+	+	-	?
Bruyndonckx, 2018	+	+	+	-	+	+	?	-	?
Moore, 2019	+	+	+	-	+	+	?	-	?

Figure S4: figure of all PF effect estimates based on univariable analysis

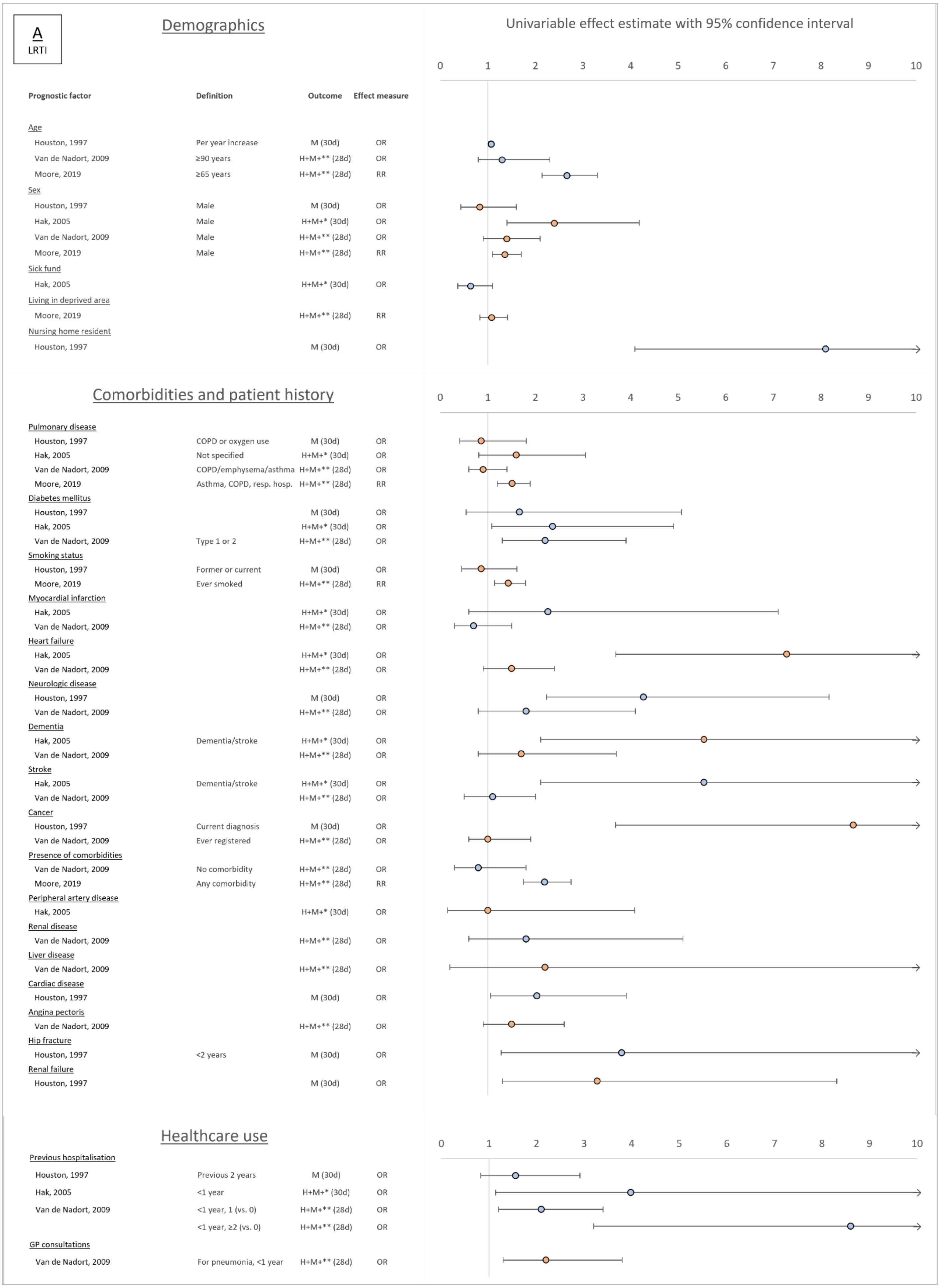

S4, continued

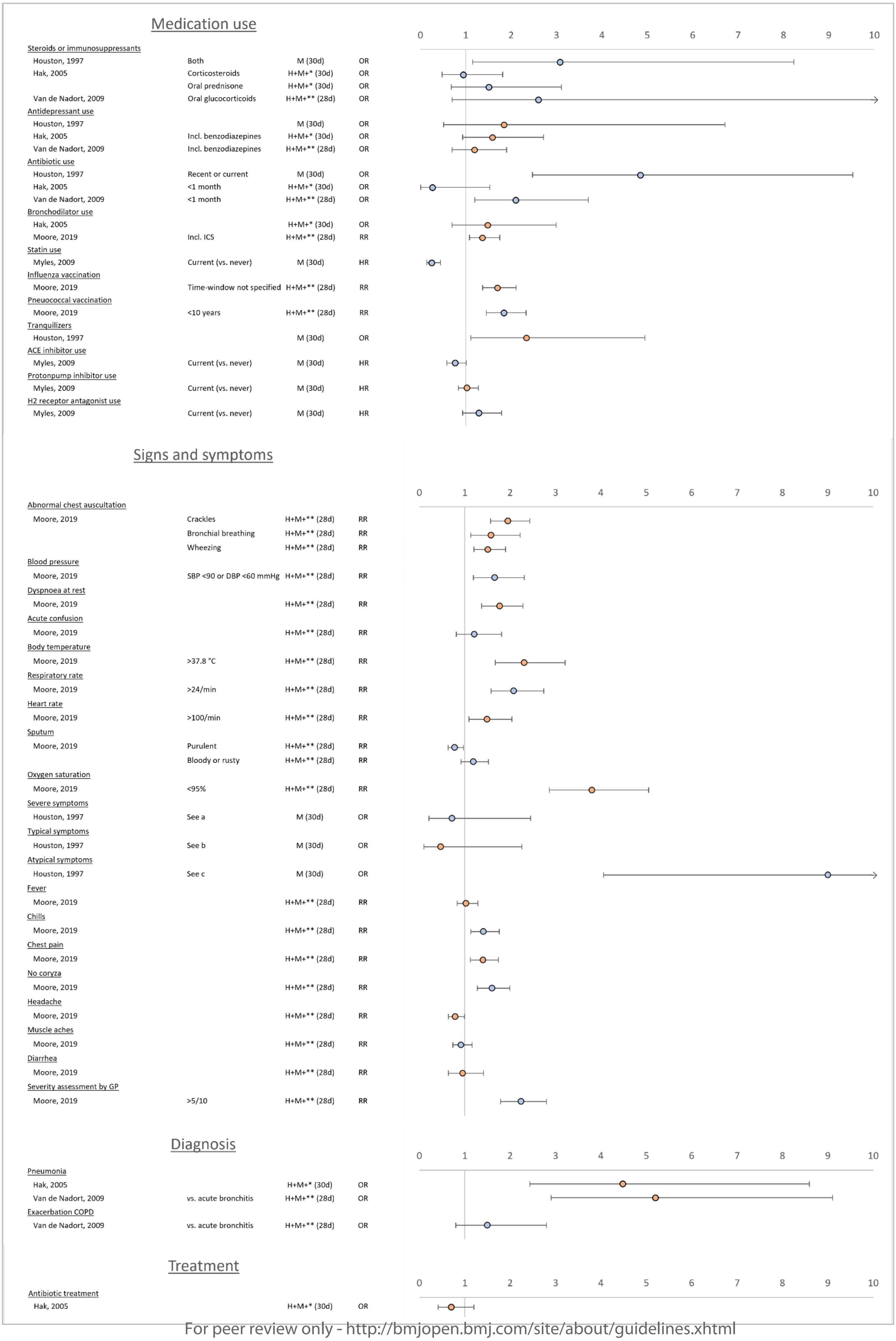

S4, continued

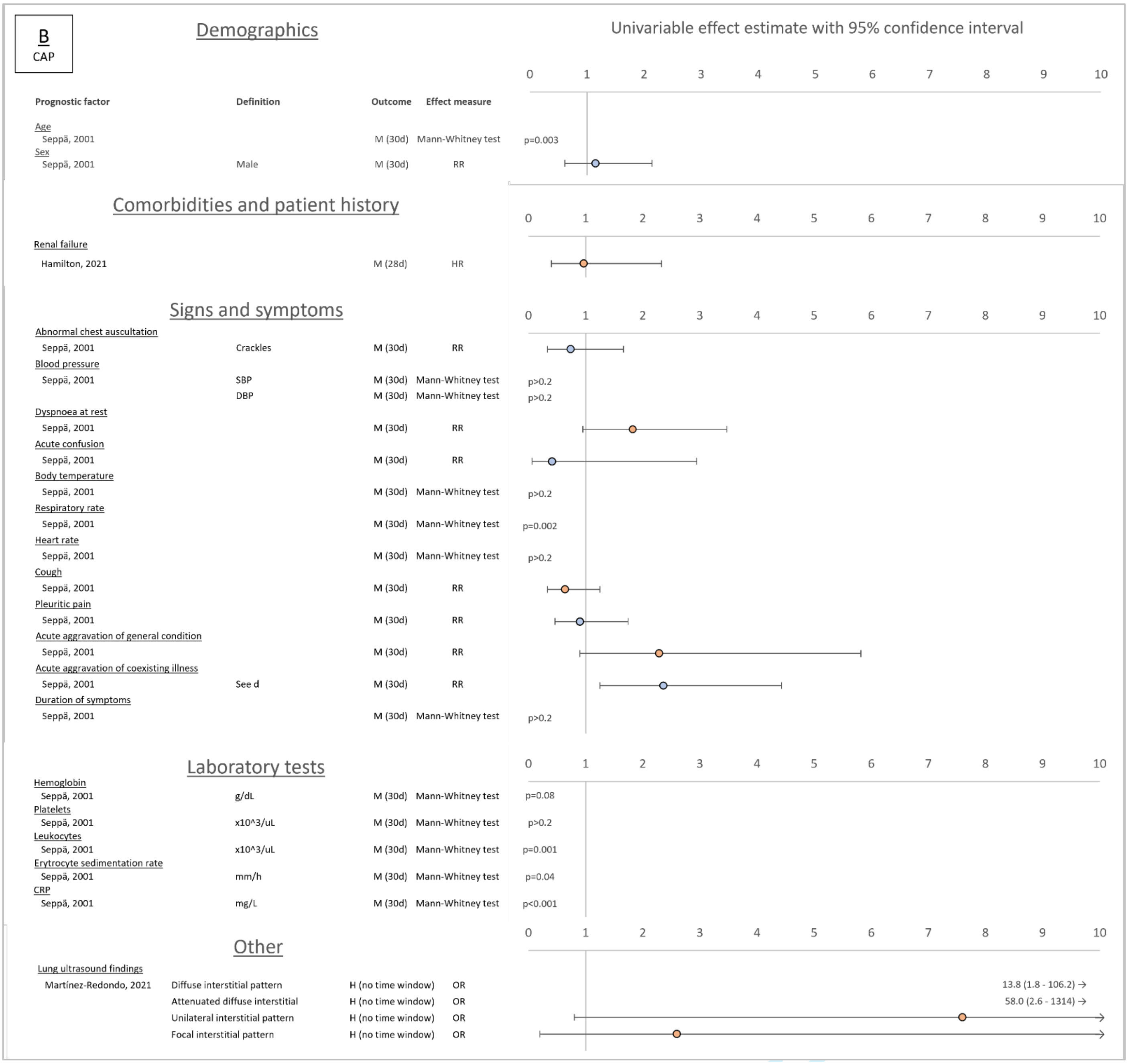

A. Univariable analyses of prognostic factors from studies on LRTI patients. B. Univariable analysis of prognostic factors from studies on pneumonia patients.
* Composite outcome also includes dysregulation of diabetes, stroke, heart failure, MI.
** Composite outcome also includes late onset pneumonia.
a: rigor, hemoptysis, pleuritic pain.
b: chills, cough, congestion, fever, dyspnea, sputum production.
c: poor eating, confusion, lethargy.
d: impairment of glucose balance in diabetic patients, deterioration of congestive heart failure.
Abbreviations: LRTI, lower respiratory tract infection; H, hospitalisation; M, mortality; OR, odds ratio; RR, risk ratio; HR, hazard ratio; COPD, chronic obstructive pulmonary disease; GP, general practitioner; ICS, inhalation corticosteroids; ACE, angiotensin converting enzyme; SBP, systolic blood pressure; DBP, diastolic blood pressure; min, minute; g, gram; dL, deciliter; uL, microliter; mm, millimeter; h, hour; L, liter.

Table S5. Absolute risks according to absence and presence of individual (categorical) prognostic factors

**Study**	Houston, 1997	Seppä, 2001	Hak, 2005	Winchester, 2009		Van de Nadort, 2009	Myles, 2009	Millett, 2015	Moore, 2019	Hamilton, 2021	Martínez-Redondo, 2021
Absolute risks provided or calculable?	No	Yes	Yes	Yes		Yes	Yes	Yes	Yes	No	Yes
Outcome definition	Mortality <30 days	Mortality <30 days	Composite including mortality <30 days	Hosp. <3 months	Mortality <3 months	Hosp. or mortality <30 days	Mortality <30 days	Hosp. <28 days	Composite including hosp. or mortality <30 days	Mortality <28 days	Hosp.
Baseline risk of outcome	10.7 %	4.0 %	14.3%	0.8 %	1.4 %	12.7 %	24.6 %	76.5 %	1.1 %	8.9 %	23.0 %
**Prognostic factor**	**Absolute risk according to prognostic factor (present/absent)**										
Demographics											
Male sex		4.3/3.7 %	20.2/9.5 %	0.8/0.7 %	1.5/1.3 %	14.2/11.8 %		79.5/73.8 %	1.3/0.9 %		
Sick fund			12.3/18.1 %								
Living in deprived area									1.2/1.1 %		
Medical history											
Heart failure			46.7/10.7 %			16.4/10.8 %		79.6/75.5 %			
Dementia/ stroke			45.0/12.9 %			17.0/12.4 %<br>12.0/12.8 %		66.8/78.2 %			
Myocardial infarct.			26.7/13.9 %			11.5/12.8 %		83.2/75.4 %			
Periph. arterial dis.			14.3/14.3 %					82.7/75.5 %			
Diabetes mellitus			26.2/13.1 %			23.1/10.8 %		82.6/75.2 %			
Prior hospitalisation			38.5/13.6 %			36.0/10.1 %					
Smoking				0.6/0.8 %	0.9/1.7 %				13.1/0.9 %		
Alcohol				0.7/0.8 %	1.1/2.3 %						
Asthma/COPD			19.7/13.3 %	0.8/0.8 %	0.7/1.5 %	12.3/12.9 %		83.4/71.8 %	1.5/1.0 %		
All. rhinitis				0.7/0.8 %	0.5/1.5 %						
Malignancies						11.9/12.8 %		80.7/75.6 %			
Angina pectoris						18.2/11.4 %		81.5/75.2 %			
Neurological dis.						18.0/12.3 %		73.1/76.8 %			
Renal disease						19.0/12.5 %		88.6/73.8 %			
Liver disease						25.0/12.6 %		85.5/76.4 %			
Connective tissue dis.								81.9/75.9 %			

For peer review only

Peptic ulcer							81.1/76.0 %	
Hemiplegia							76.4/76.5 %	
Leukaemia/lymphoma							85.0/76.2	
Cerebrovascular dis.							74.5/77.2 %	
Terminal illness							67.1/76.9 %	
Medication use								
Antidepressant/ benzodiazepines		18.1/12.2 %			15.4/11.6 %			
Corticosteroids		13.8/14.4 %						
Oral prednisone		19.2/13.6 %			37.5/11.7 %		83.0/75.4 %	
Immunosuppressants							85.3/76.3 %	
ICS							84.7/74.6 %	
Bronchodilator		19.0/13.6 %						1.4/1.0 %
Recent antibiotics		4.5/14.8 %	2.8/0.7 %	3.4/1.4 %	24.2/11.8 %		74.8/77.2 %	
Vaccination								
Influenza			0.9/0.4 %	2.4/0.8 %			76.2/69.7 %	1.5/0.9 %
Pneumococcal			1.0/0.7 %	2.6/1.1 %			81.3/33.6 %	1.8/1.0 %
Statin use						6.8/25.5 %		
ACEi use						18.6/25.1 %		
PPI use						26.1/24.4 %		
H2 antagonist						31.7/24.3 %		
Presenting signs and symptoms								
Cough	3.6/5.6 %							
Dyspnoea	5.3/2.9 %							1.3/0.8 %
Pleuritic pain	3.8/4.2 %							
Confusion	1.8/4.4 %							1.3/1.1 %
Aggravation of								
General condition	3.7/4.7 %							
Coexisting illness	7.6/3.2 %							
Abn. auscultation	3.7/5.0%							
Fever								1.1/1.1 %
Chills								1.4/1.0 %
Chest pain								1.4/1.0 %
No coryza								1.4/0.9 %
Muscle aches								1.1/1.2 %
Diarrhoea								1.1/1.1 %
Purulent sputum								1.0/1.3 %
Bloody sputum								1.6/1.1 %
Severity assessment >5/10								1.7/0.7 %
Respiratory rate >24/min								2.1/1.0 %

For peer review only

Temperature >37.8				2.4/0/1 %	
Pulse >100/min				1.6/1.1 %	
O_2 saturation <95 %				3.5/0.9 %	
SBP<90 or DBP<60 mmHg				1.8/1.1 %	
Crackles				15.7/0.8 %	
Bronchial breathing				1.7/1.1 %	
Wheezing				1.5/1.0 %	
LRTI diagnosis					
Pneumonia	22.6/6.1 %	27.3/8.0 %			
Exacerbation COPD		9.9/14.5 %			
Acute bronchitis		5.8/16.5 %			
Frailty factors					
Recent carer			79.6/76.3 %		
Visual impairment			78.8/75.3 %		
Self-care			79.7/76.4 %		
Anxious/depressed			76.0/76.5 %		
Bedsore/ulcer			59.7/77.0 %		
Mobility issues			79.0/76.3 %		
Tired			74.4/76.6 %		
Low weight/nutrition			75.1/76.7 %		
Incontinence			71.7/77.0		
History of falling			76.4/76.5 %		
Lung ultrasound					
Abnormal					28.2/3.33 %

Abbreviations: NR, not reported; COPD, chronic obstructive pulmonary disease; ICS, inhaled corticosteroids; ACEi, angiotensin converting enzyme inhibitor; PPI, protonpump inhibitor; H2, histamine-2; SBP, systolic blood pressure; DBP, diastolic blood pressure; LRTI, lower respiratory tract infection.

For peer review only

Figure S6: figure of all PF effect estimates based on multivariable effect estimates

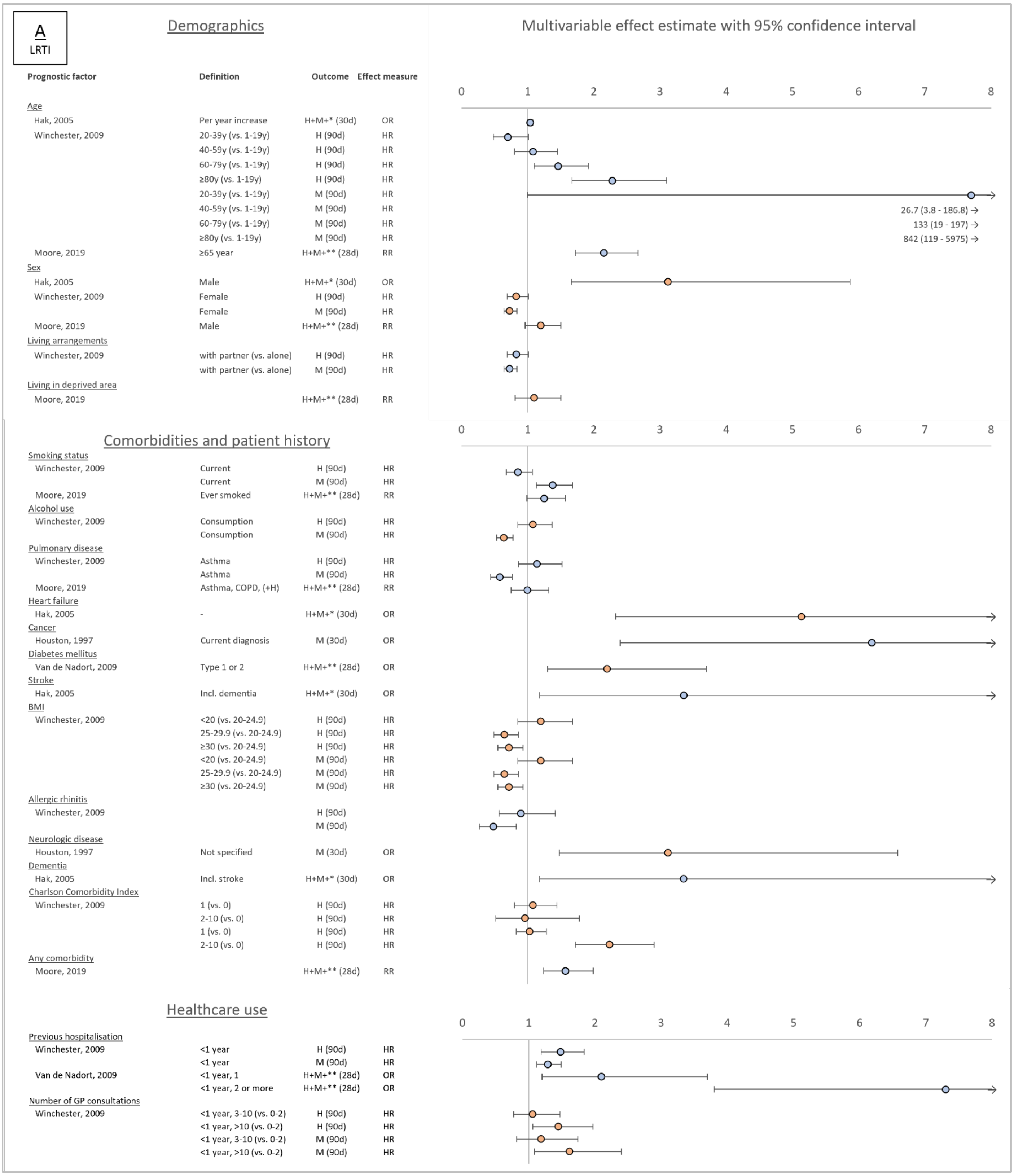

S6, continued

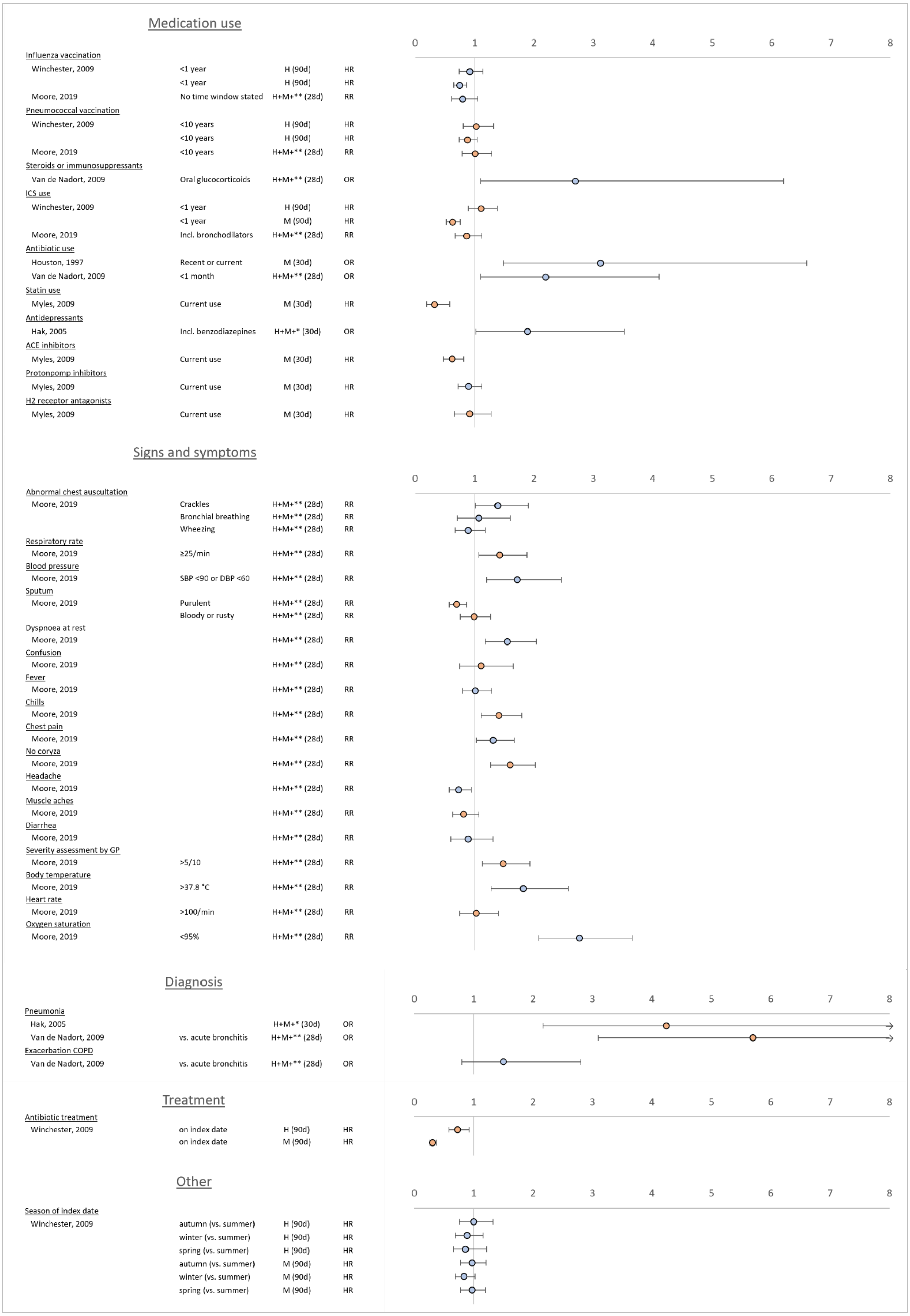

S6, continued

B
CAP

Demographics

Prognostic factor	Definition	Outcome	Effect measure
Age			
Millett, 2015	70-74y (vs. 65-69y)	H (28d)	OR
	75-79y (vs. 65-69y)	H (28d)	OR
	80-84y (vs. 65-69y)	H (28d)	OR
	85-89y (vs. 65-69y)	H (28d)	OR
	≥90y (vs. 65-69y)	H (28d)	OR
Hamilton, 2021	Per year increase	M (28d)	HR
Sex			
Hamilton, 2021	Female	M (28d)	HR
Living arrangements			
Millett, 2015	Alone	H (28d)	OR
	Sheltered accomodation	H (28d)	OR
	Residential care	H (28d)	OR

Comorbidities and patient history

Prognostic factor	Definition	Outcome	Effect measure
Smoking status			
Millett, 2015	Current (vs. never)	H (28d)	OR
	Former (vs. never)	H (28d)	OR
Hamilton, 2021	Former (vs. never)	M (28d)	HR
	Current (vs. never)	M (28d)	HR
Alcohol use			
Millett, 2015	Excessive use	H (28d)	OR
Hamilton, 2021	Excessive use	M (28d)	HR
Cancer			
Millett, 2015	Solid, non-metastatic	H (28d)	OR
	Solid, metastatic	H (28d)	OR
	Leukemia/lymphoma	H (28d)	OR
Hamilton, 2021	Solid	M (28d)	HR
	Haematological	M (28d)	HR
Diabetes mellitus			
Millett, 2015	No complications	H (28d)	OR
	Complications	H (28d)	OR
Hamilton, 2021	Type 1 or 2	M (28d)	HR
Stroke			
Millett, 2015	Cerebrovascular disease	H (28d)	OR
Hamilton, 2021	Ischaemic stroke	M (28d)	HR
Ischaemic heart disease			
Millett, 2015	Pre MI	H (28d)	OR
	Post MI	H (28d)	OR
Hamilton, 2021		M (28d)	HR
Peripheral vascular disease			
Millett, 2015		H (28d)	OR
Hamilton, 2021		M (28d)	
Pulmonary disease			
Millett, 2015	COPD, emphysema, asthma	H (28d)	OR
Neurologic disease			
Millett, 2015		H (28d)	OR
Dementia			
Millett, 2015		H (28d)	OR
Terminal illness			
Millett, 2015		H (28d)	OR
Heart failure			
Millett, 2015		H (28d)	OR
Renal disease			
Millett, 2015	Mild	H (28d)	OR
	Severe	H (28d)	OR
Liver disease			
Millett, 2015	Mild	H (28d)	OR
	Severe	H (28d)	OR
Connective tissue disease			
Millett, 2015		H (28d)	OR
Immune mechanism disorders			
Millett, 2015		H (28d)	OR
Autoimmunity			
Hamilton, 2021		M (28d)	HR
Solid organ transplant			
Hamilton, 2021		M (28d)	HR
Peptic ulcer			
Millett, 2015		H (28d)	OR
Hemiplegia			
Millett, 2015		H (28d)	OR
Depression			
Millett, 2015		H (28d)	OR
Indicators of frailty			
Millett, 2015	Recent carer	H (28d)	OR
	Self-care problems	H (28d)	OR
	Low weight/poor nutrition	H (28d)	OR
	Bedsore/ulcer	H (28d)	OR
	Mobility issues	H (28d)	OR
	incontinence/catheter	H (28d)	OR
	Visual impairment	H (28d)	OR
	History of falling	H (28d)	OR

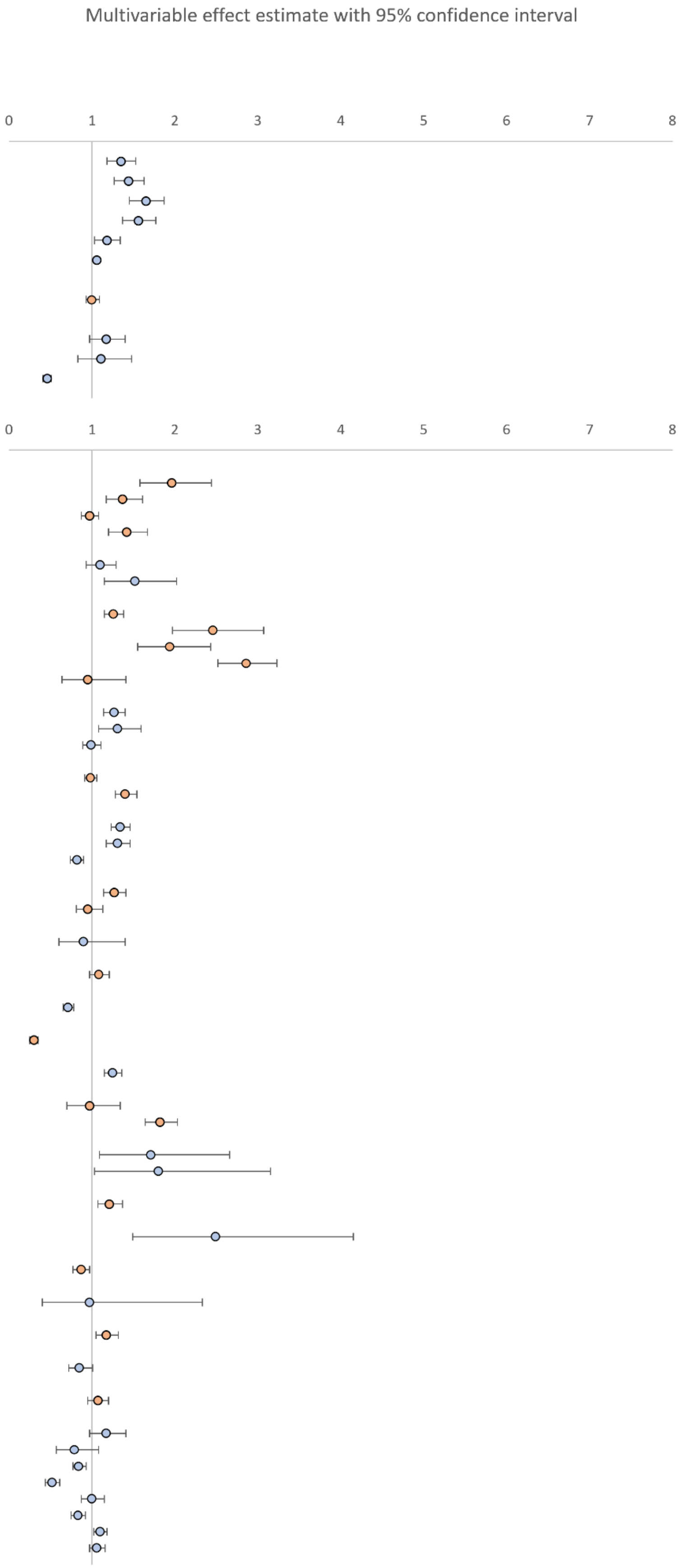

S6, continued

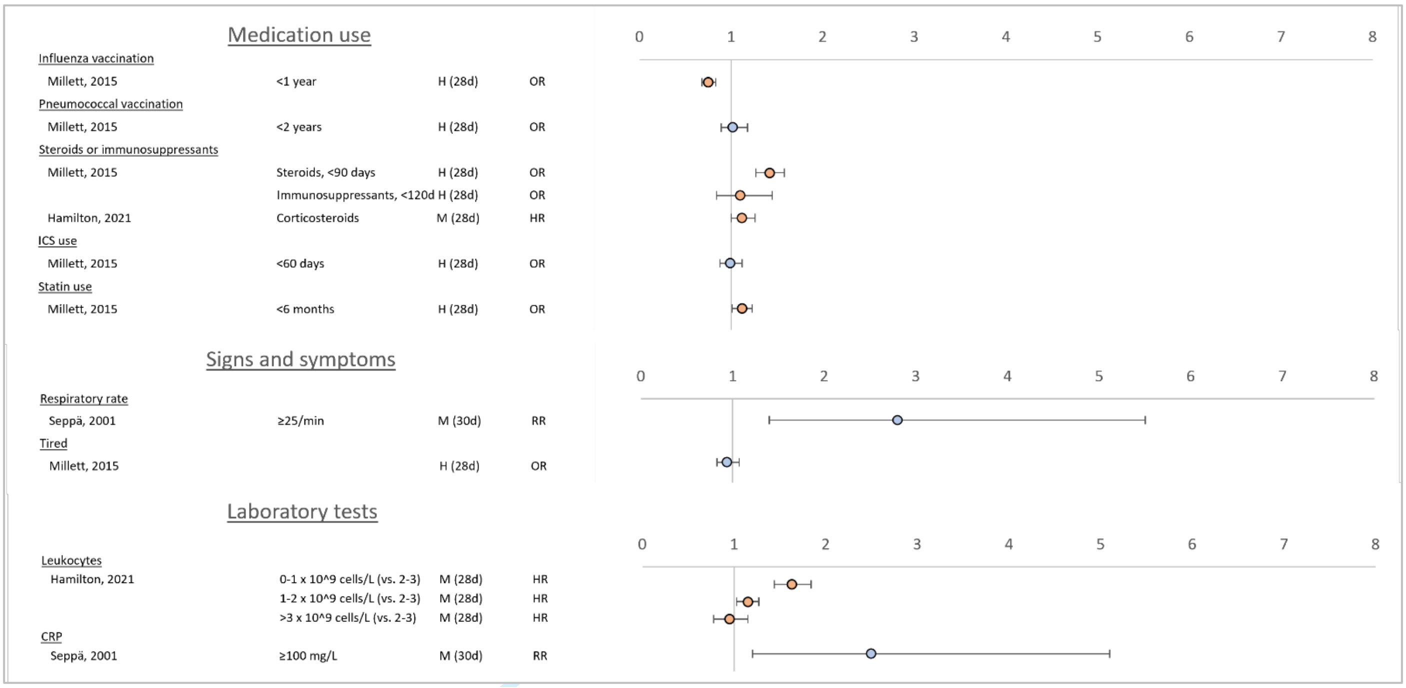

A. Multivariable analyses of prognostic factors from studies on LRTI patients. B. Multivariable analysis of prognostic factors from studies on pneumonia patients.
* Composite outcome also includes dysregulation of diabetes, stroke, heart failure, MI.
** Composite outcome also includes late onset pneumonia.
Abbreviations: LRTI, lower respiratory tract infection; H, hospitalisation; M, mortality; OR, odds ratio; RR, risk ratio; HR, hazard ratio; COPD, chronic obstructive pulmonary disease; BMI, body mass index; MI, myocardial infarction; GP, general practitioner; ICS, inhalation corticosteroids; ACE, angiotensin converting enzyme; SBP, systolic blood pressure; DBP, diastolic blood pressure; min, minute; L, liter; mg, milligram.

For peer review only

Table S7: Rating of the quality of evidence on promising prognostic factors based on the GRADE framework

Prognostic factor	GRADE criteria							Quality of evidence
	Study design	Risk of bias	Inconsistency	Indirectness	Imprecision	Publication bias	Upgrading factors	
Age	Cohort (high quality)	-2	0	0	0	0	0	Low
Sex	Cohort (high quality)	-2	0	-1	-1	0	0	Very low
Smoking status	Cohort (high quality)	-2	-1	-1	-1	0	0	Very low
Diabetes	Cohort (high quality)	-2	0	-1	0	0	0	Very low
Stroke	Cohort (high quality)	-2	-1	-1	-1	0	0	Very low
Cancer	Cohort (high quality)	-2	0	-1	0	0	0	Very low
Heart failure	Cohort (high quality)	-2	0	-1	0	0	0	Very low
Previous hospitalisation	Cohort (high quality)	-2	0	0	0	0	0	Low
Systemic corticosteroids	Cohort (high quality)	-2	0	-1	-1	0	0	Very low
Influenza vaccination	Cohort (high quality)	-2	0	0	-1	0	0	Very low
Recent antibiotic use	Cohort (high quality)	-2	0	0	-1	0	0	Very low
Respiratory rate	Cohort (high quality)	-2	0	-1	0	0	0	Very low
Diagnosis of pneumonia	Cohort (high quality)	-2	0	0	0	0	0	Low

Certainty of evidence			
High	Moderate	Low	Very low
4	3	2	1

Application of the GRADE framework to rate the quality of evidence was based on prognostic research-specific guidance (Foroutan et al., 2020).

Abbreviations: GRADE, grading of recommendations, assessment, development, and evaluations.

For peer review only

Table S8: Overview of prediction models included in the synthesis

Prediction model	Predictors included in model	Predictor weight
Bont, 2007 (new)	- Diagnosis (either acute bronchitis, COPD exacerbation, or pneumonia)	0, 2, or 4
	- Age ≥80 years	2
	- Congestive heart failure	1
	- Diabetes	2
	- Oral glucocorticoid use	3
	- Hospitalisations in previous year (0, 1, or ≥2)	0, 2, or 3
	- Antibiotic use in previous month	2
CRB(-65)	- Confusion	1
	- Respiratory rate ≥30/minute	1
	- Blood pressure (SBP ≤90mmHg or DBP ≤60mmHg)	1
	(- Age ≥65 years)	1
CURB(-65)	- Predictors of CRB(-65)	0-4
	- Blood urea nitrogen >7 mmol/L	1
PSI (stage 1)	- Age >50 years	Any versus none
	- Altered mental status	
	- Pulse >125/minute	
	- Respiratory rate >30/minute	
	- SBP <90mmHg	
	- Temperature <35°C or ≥40°C	
	- Neoplastic disease	
	- Congestive heart failure	
	- Cerebrovascular disease	
	- Renal disease	
	- Liver disease	
CORB-75 (new)	- Confusion	1
	- Peripheral oxygen saturation ≤90%	1
	- Respiratory rate ≥30/minute	1
	- Blood pressure (SBP ≤90mmHg or DBP ≤60mmHg)	1
	- Age ≥75 years	1
RISSC85 (new)	- Risk of poor outcome, grouped by country (A: Spain, B: Belgium, the Netherlands, Poland, UK, C: Germany)	A = 0, B/C = 1
	- Interference in daily activities (some versus severe)	1
	- Number of years stopped smoking (>45 years)	1
	- Severe sputum	1
	- Presence of crackles	1
	- Diastolic blood pressure (<85mmHg)	1
Moore, 2019 (new)	- Oxygen saturation <95%	1
	- Age >65 years	1
	- Blood pressure (SBP <90mmHg or DBP <60mmHg)	1
	- Temperature >37.8°C	1
	- Any comorbidity (cardiovascular, cerebrovascular or lung comorbidities)	1
	- No coryza	1
	- Severity assessment >5/10 by GP	1
	- Chest pain	1

Abbreviations: COPD, chronic obstructive pulmonary disease; SBP, systolic blood pressure; DBP, diastolic blood pressure; GP, general practitioner.

For peer review only

Table S9: Rating of the quality of evidence on prediction models based on the GRADE framework

Prediction model	GRADE criteria							Quality of evidence
	Study design	Risk of bias	Inconsistency	Indirectness	Imprecision	Publication bias	Upgrading factors	
Bont, 2007	Cohort (high quality)	-2	0	0	-1	0	0	Very low
CRB	Cohort (high quality)	-2	0	-1	-1	0	0	Very low
CRB-65	Cohort (high quality)	-2	0	0	0	0	0	Low
CURB	Cohort (high quality)	-2	0	-1	-1	0	0	Very low
CURB-65	Cohort (high quality)	-2	0	-1	-1	0	0	Very low
PSI	Cohort (high quality)	-2	0	-1	-1	0	0	Very low
CORB-75	Cohort (high quality)	-2	0	-1	-1	0	0	Very low
RISCC85	Cohort (high quality)	-2	0	-1	-1	0	0	Very low
Moore, 2019	Cohort (high quality)	-2	0	-1	-1	0	0	Very low

Certainty of evidence			
High	Moderate	Low	Very low
4	3	2	1

Application of the GRADE framework to rate the quality of evidence was based on prognostic research-specific guidance (Brozek et al., 2021).

Abbreviations: GRADE, grading of recommendations, assessment, development, and evaluations.

For peer review only

PRISMA 2020 Checklist

Section and Topic	Item #	Checklist item	Location where item is reported
**TITLE**			
Title	1	Identify the report as a systematic review.	1
**ABSTRACT**			
Abstract	2	See the PRISMA 2020 for Abstracts checklist.	3
**INTRODUCTION**			
Rationale	3	Describe the rationale for the review in the context of existing knowledge.	6
Objectives	4	Provide an explicit statement of the objective(s) or question(s) the review addresses.	6
**METHODS**			
Eligibility criteria	5	Specify the inclusion and exclusion criteria for the review and how studies were grouped for the syntheses.	7
Information sources	6	Specify all databases, registers, websites, organisations, reference lists and other sources searched or consulted to identify studies. Specify the date when each source was last searched or consulted.	7
Search strategy	7	Present the full search strategies for all databases, registers and websites, including any filters and limits used.	S1
Selection process	8	Specify the methods used to decide whether a study met the inclusion criteria of the review, including how many reviewers screened each record and each report retrieved, whether they worked independently, and if applicable, details of automation tools used in the process.	7
Data collection process	9	Specify the methods used to collect data from reports, including how many reviewers collected data from each report, whether they worked independently, any processes for obtaining or confirming data from study investigators, and if applicable, details of automation tools used in the process.	7, 8
Data items	10a	List and define all outcomes for which data were sought. Specify whether all results that were compatible with each outcome domain in each study were sought (e.g. for all measures, time points, analyses), and if not, the methods used to decide which results to collect.	7, 8
	10b	List and define all other variables for which data were sought (e.g. participant and intervention characteristics, funding sources). Describe any assumptions made about any missing or unclear information.	8
Study risk of bias assessment	11	Specify the methods used to assess risk of bias in the included studies, including details of the tool(s) used, how many reviewers assessed each study and whether they worked independently, and if applicable, details of automation tools used in the process.	8
Effect measures	12	Specify for each outcome the effect measure(s) (e.g. risk ratio, mean difference) used in the synthesis or presentation of results.	7, 8
Synthesis methods	13a	Describe the processes used to decide which studies were eligible for each synthesis (e.g. tabulating the study intervention characteristics and comparing against the planned groups for each synthesis (item #5)).	7, 8
	13b	Describe any methods required to prepare the data for presentation or synthesis, such as handling of missing summary statistics, or data conversions.	7, 8
	13c	Describe any methods used to tabulate or visually display results of individual studies and syntheses.	8, 9
	13d	Describe any methods used to synthesize results and provide a rationale for the choice(s). If meta-analysis was performed, describe the model(s), method(s) to identify the presence and extent of statistical heterogeneity, and software package(s) used.	8, 9
	13e	Describe any methods used to explore possible causes of heterogeneity among study results (e.g. subgroup analysis, meta-regression).	N/A
	13f	Describe any sensitivity analyses conducted to assess robustness of the synthesized results.	N/A
Reporting bias assessment	14	Describe any methods used to assess risk of bias due to missing results in a synthesis (arising from reporting biases).	N/A
Certainty	15	Describe any methods used to assess certainty (or confidence) in the body of evidence for an outcome.	N/A

For peer review only - http://bmjopen.bmj.com/site/about/guidelines.xhtml

PRISMA 2020 Checklist

Section and Topic	Item #	Checklist item	Location where item is reported
assessment			
**RESULTS**			
Study selection	16a	Describe the results of the search and selection process, from the number of records identified in the search to the number of studies included in the review, ideally using a flow diagram.	10, Figure 1
	16b	Cite studies that might appear to meet the inclusion criteria, but which were excluded, and explain why they were excluded.	N/A
Study characteristics	17	Cite each included study and present its characteristics.	10, Table 2-3
Risk of bias in studies	18	Present assessments of risk of bias for each included study.	11, Figure S2-3
Results of individual studies	19	For all outcomes, present, for each study: (a) summary statistics for each group (where appropriate) and (b) an effect estimate and its precision (e.g. confidence/credible interval), ideally using structured tables or plots.	11-13, Figure 2-3, Figure S4-6
Results of syntheses	20a	For each synthesis, briefly summarise the characteristics and risk of bias among contributing studies.	11
	20b	Present results of all statistical syntheses conducted. If meta-analysis was done, present for each the summary estimate and its precision (e.g. confidence/credible interval) and measures of statistical heterogeneity. If comparing groups, describe the direction of the effect.	11-13, Figure 2-3, Figure S4-6
	20c	Present results of all investigations of possible causes of heterogeneity among study results.	N/A
	20d	Present results of all sensitivity analyses conducted to assess the robustness of the synthesized results.	N/A
Reporting biases	21	Present assessments of risk of bias due to missing results (arising from reporting biases) for each synthesis assessed.	N/A
Certainty of evidence	22	Present assessments of certainty (or confidence) in the body of evidence for each outcome assessed.	N/A
**DISCUSSION**			
Discussion	23a	Provide a general interpretation of the results in the context of other evidence.	14, 15
	23b	Discuss any limitations of the evidence included in the review.	16
	23c	Discuss any limitations of the review processes used.	16
	23d	Discuss implications of the results for practice, policy, and future research.	17
**OTHER INFORMATION**			
Registration and protocol	24a	Provide registration information for the review, including register name and registration number, or state that the review was not registered.	3, 7
	24b	Indicate where the review protocol can be accessed, or state that a protocol was not prepared.	3, 7
	24c	Describe and explain any amendments to information provided at registration or in the protocol.	N/A
Support	25	Describe sources of financial or non-financial support for the review, and the role of the funders or sponsors in the review.	18
Competing interests	26	Declare any competing interests of review authors.	18
Availability of data, code and other materials	27	Report which of the following are publicly available and where they can be found: template data collection forms; data extracted from included studies; data used for all analyses; analytic code; any other materials used in the review.	18

For peer review only - http://bmjopen.bmj.com/site/about/guidelines.xhtml

For peer review only

PRISMA 2020 Checklist

From: Page MJ, McKenzie JE, Bossuyt PM, Boutron I, Hoffmann TC, Mulrow CD, et al. The PRISMA 2020 statement: an updated guideline for reporting systematic reviews. BMJ 2021;372:n71. doi: 10.1136/bmj.n71

For more information, visit: http://www.prisma-statement.org/

For peer review only

PROSPERO
International prospective register of systematic reviews

National Institute for Health Research

University of York
Centre for Reviews and Dissemination

Systematic review

A list of fields that can be edited in an update can be found here

1. * Review title.

Give the title of the review in English

Prognosis of lower respiratory tract infections in adults presenting to primary care: a systematic review of prognostic factors and prediction models

2. Original language title.

For reviews in languages other than English, give the title in the original language. This will be displayed with the English language title.

3. * Anticipated or actual start date.

Give the date the systematic review started or is expected to start.

17/06/2022

4. * Anticipated completion date. [1 change]

Give the date by which the review is expected to be completed.

01/04/2023

5. * Stage of review at time of this submission. [3 changes]

This field uses answers to initial screening questions. It cannot be edited until after registration.

Tick the boxes to show which review tasks have been started and which have been completed.

Update this field each time any amendments are made to a published record.

The review has not yet started: No

For peer review only

PROSPERO
International prospective register of systematic reviews

National Institute for Health Research

Review stage	Started	Completed
Preliminary searches	Yes	Yes
Piloting of the study selection process	Yes	Yes
Formal screening of search results against eligibility criteria	Yes	Yes
Data extraction	Yes	Yes
Risk of bias (quality) assessment	Yes	Yes
Data analysis	Yes	Yes

Provide any other relevant information about the stage of the review here.

6. * Named contact.

The named contact is the guarantor for the accuracy of the information in the register record. This may be any member of the review team.

Merijn Rijk

Email salutation (e.g. "Dr Smith" or "Joanne") for correspondence:

Merijn Rijk

7. * Named contact email.

Give the electronic email address of the named contact.

m.h.rijk@umcutrecht.nl

8. Named contact address

Give the full institutional/organisational postal address for the named contact.

P.O. Box 85500, 3508 GA Utrecht

9. Named contact phone number.

Give the telephone number for the named contact, including international dialling code.

0031647424873

10. * Organisational affiliation of the review.

Full title of the organisational affiliations for this review and website address if available. This field may be

For peer review only

completed as 'None' if the review is not affiliated to any organisation.

Julius Center for Health Sciences and Primary Care, UMC Utrecht

Organisation web address:

11. * Review team members and their organisational affiliations. [1 change]

Give the personal details and the organisational affiliations of each member of the review team. Affiliation refers to groups or organisations to which review team members belong. **NOTE: email and country now MUST be entered for each person, unless you are amending a published record.**

Mr Merijn Rijk. Julius Center for Health Sciences and Primary Care, UMC Utrecht
Mr Teun van den Berg. Julius Center for Health Sciences and Primary Care, UMC Utrecht
Dr Tamara Platteel. Julius Center for Health Sciences and Primary Care, UMC Utrecht
Dr Roderick Venekamp. Julius Center for Health Sciences and Primary Care, UMC Utrecht
Dr Maarten van Smeden. Julius Center for Health Sciences and Primary Care, UMC Utrecht
Dr Geert-Jan Geersing. Julius Center for Health Sciences and Primary Care, UMC Utrecht
Professor Frans Rutten. Julius Center for Health Sciences and Primary Care, UMC Utrecht
Dr Monika Hollander. Julius Center for Health Sciences and Primary Care, UMC Utrecht
Professor Paul Little. University of Southampton
Dr Bert Dalmolen.

For peer review only

12. * Funding sources/sponsors.

Details of the individuals, organizations, groups, companies or other legal entities who have funded or sponsored the review.

The Netherlands Organisation for Health Research and Development (ZonMw), Huisartsgeneeskunde 2021, no. 08391052110003

Grant number(s)

State the funder, grant or award number and the date of award

ZonMw, General Practice and Elderly Care grant, round of 2021

13. * Conflicts of interest.

List actual or perceived conflicts of interest (financial or academic).

None

14. Collaborators.

Give the name and affiliation of any individuals or organisations who are working on the review but who are not listed as review team members. **NOTE: email and country must be completed for each person, unless you are amending a published record.**

PROSPERO
International prospective register of systematic reviews

National Institute for Health Research

15. * Review question.

State the review question(s) clearly and precisely. It may be appropriate to break very broad questions down into a series of related more specific questions. Questions may be framed or refined using PI(E)COS or similar where relevant.

1) What are prognostic factors for a complicated trajectory (i.e. hospitalization or death) within 90 days in adult patients with lower respiratory tract infections (LRTI) presenting to primary care?

P: adult patients with LRTI

I: any patient or disease specific predictor

C: -

O: Hospitalisation or death

T1: prognostic factor present at first consultation of LRTI episode

T2: outcome within 90 days

2) What prediction models have been developed to estimate individual risk of hospitalization and/or death within 90 days in adult patients with a LRTI presenting to primary care?

P: adult patients with LRTI

I: any developed prediction model

C: -

O: Hospitalisation or death

T1: prediction at first consultation of LRTI episode

T2: outcome within 90 days

S: primary care

16. * Searches.

State the sources that will be searched (e.g. Medline). Give the search dates, and any restrictions (e.g. language or publication date). Do NOT enter the full search strategy (it may be provided as a link or attachment below.)

For peer review only

MEDLINE, Embase, and Cochrane Library. Restrictions: no restrictions in search strategy.

17. URL to search strategy.

Upload a file with your search strategy, or an example of a search strategy for a specific database, (including the keywords) in pdf or word format. In doing so you are consenting to the file being made publicly accessible. Or provide a URL or link to the strategy. Do NOT provide links to your search **results**.

https://www.crd.york.ac.uk/PROSPEROFILES/341233_STRATEGY_20220628.pdf

Alternatively, upload your search strategy to CRD in pdf format. Please note that by doing so you are consenting to the file being made publicly accessible.

Do not make this file publicly available until the review is complete

18. * Condition or domain being studied.

Give a short description of the disease, condition or healthcare domain being studied in your systematic review.

Prognosis of lower respiratory tract infections in adults presenting to primary care

19. * Participants/population.

Specify the participants or populations being studied in the review. The preferred format includes details of both inclusion and exclusion criteria.

Adults with lower respiratory tract infections presenting to primary care

20. * Intervention(s), exposure(s).

Give full and clear descriptions or definitions of the interventions or the exposures to be reviewed. The preferred format includes details of both inclusion and exclusion criteria.

For review question 1): any patient or disease specific prognostic factor
For review question 2): any developed or updated prediction model applicable to a primary care setting

21. * Comparator(s)/control.

Where relevant, give details of the alternatives against which the intervention/exposure will be compared (e.g. another intervention or a non-exposed control group). The preferred format includes details of both inclusion and exclusion criteria.

Not relevant

22. * Types of study to be included.

Give details of the study designs (e.g. RCT) that are eligible for inclusion in the review. The preferred format includes both inclusion and exclusion criteria. If there are no restrictions on the types of study, this should be stated.

For review question 1): prognostic factor studies
For review question 2): model development and validation studies

For peer review only

PROSPERO
International prospective register of systematic reviews

National Institute for Health Research

23. Context.

Give summary details of the setting or other relevant characteristics, which help define the inclusion or exclusion criteria.

For review question 1):

- Prognostic factor studies conducted within (or applicable to) a primary care setting

For review question 2):

- Model development and/or validation studies conducted within (or applicable to) a primary care setting
- Validation or updating of secondary care derived prediction models in primary care

24. * Main outcome(s).

Give the pre-specified main (most important) outcomes of the review, including details of how the outcome is defined and measured and when these measurement are made, if these are part of the review inclusion criteria.

Hospitalisation or (all-cause) death within 90 days after initial presentation to primary care

Measures of effect

Please specify the effect measure(s) for you main outcome(s) e.g. relative risks, odds ratios, risk difference, and/or 'number needed to treat.

For review question 1): prognostic effect measures (odds ratios and hazard ratios), both unadjusted and adjusted

For review question 2): data on discrimination (C statistic) and calibration (O:E ratio, intercept, slope)

25. * Additional outcome(s).

List the pre-specified additional outcomes of the review, with a similar level of detail to that required for main outcomes. Where there are no additional outcomes please state 'None' or 'Not applicable' as appropriate to the review

Not applicable

Measures of effect

Please specify the effect measure(s) for you additional outcome(s) e.g. relative risks, odds ratios, risk difference, and/or 'number needed to treat.

26. * Data extraction (selection and coding).

Describe how studies will be selected for inclusion. State what data will be extracted or obtained. State how this will be done and recorded.

1) Study selection:

Clinical domain, outcome, and setting match PICOTS, study design: prognostic factor study or prognostic model development or validation study

- Exclusion: full-text not available, language other than English or Dutch

2) Screening for eligible studies by two researchers, independently and blinded. Screening process: 1) title/abstract, 2) full-text, 3) backward and forward citation searching of included studies.

3) Disagreements resolved through discussion and, if necessary, after consultation of third researcher (supervisor)

4) Software system: de-duplication of yielded studies and title/abstract screening using Rayyan (online application)

Data extraction:

1) Data extraction based on CHARMS (prediction models) and CHARMS-PF (prognostic factor studies) checklists

2) Extraction of data by two researchers: extraction of data by researcher 1, check of extracted data by researcher 2

3) Disagreements will be resolved through discussion and, if neccesary, after consultation of a third researcher (supervisor)

4) Missing data on measurements of effect will be either calculated from raw data if possible or requested from researchers of the primary study

27. * Risk of bias (quality) assessment.

State which characteristics of the studies will be assessed and/or any formal risk of bias/quality assessment tools that will be used.

For risk of bias assessment the following formal tools will be used:
- For review question 1): QUIPS

- For review question 2): PROBAST

28. * Strategy for data synthesis.
[1 change]

Describe the methods you plan to use to synthesise data. This **must not be generic text** but should be **specific to your review** and describe how the proposed approach will be applied to your data. If meta-analysis is planned, describe the models to be used, methods to explore statistical heterogeneity, and software package to be used.

For review question 1):

For peer review only

Data synthesis will not be performed for prognostic factors due to 1) the broad search for 'any' prognostic factor and 2) the anticipated heterogeneity of the domain and prognostic factor definition. Effect measures (unadjusted and adjusted) of prognostic factors will be summarised in a forest plot.

For review question 2):

Data synthesis will not be performed. Prediction models will be evaluated and compared based on:

- Risk of bias in the development (using PROBAST tool)

- Applicability to the clinical patient and setting (PICOTS and review question)

- Performance of the model (based on the performance measures mentioned in field #24)

- Method of validation (internal, internal-external, external).

Based on these criteria conclusions will be drawn with regard to the usefulness of the prediction models to clinical practice. This will provide guidance for clinicians to choose the appropriate tool (if any of the prediction models proves to be robust) for prediction of hospitalization or death in LRTI patients.

29. * Analysis of subgroups or subsets.

State any planned investigation of 'subgroups'. Be clear and specific about which type of study or participant will be included in each group or covariate investigated. State the planned analytic approach.

Studies for both review questions will be stratified with regard to data collection on (possible) Coronavirus disease 2019 (COVID-19) patients, to assess any COVID-19 specific effect (results:

- Non-COVID-19 data (data collection up to and including 2019 and data on (explicitly stated) non-COVID-19 patients collected from 2020 onwards)

- COVID-19 data (data collection from 2020 onwards on (possible) COVID-19 patients)

30. * Type and method of review.

Select the type of review, review method and health area from the lists below.

Type of review

Cost effectiveness
No

Diagnostic
No

Epidemiologic
No

Individual patient data (IPD) meta-analysis

No

Intervention

No

Living systematic review

No

Meta-analysis

No

Methodology

No

Narrative synthesis

No

Network meta-analysis

No

Pre-clinical

No

Prevention

No

Prognostic

Yes

Prospective meta-analysis (PMA)

No

Review of reviews

No

Service delivery

No

Synthesis of qualitative studies

No

Systematic review

Yes

Other

No

Health area of the review

Alcohol/substance misuse/abuse

No

Blood and immune system

For peer review only

No

Cancer

No

Cardiovascular

No

Care of the elderly

No

Child health

No

Complementary therapies

No

COVID-19

Yes

For COVID-19 registrations please tick all categories that apply. Doing so will enable your record to appear in area-specific searches

Chinese medicine
Diagnosis
Epidemiological
Genetics
Health impacts
Immunity
Long COVID
Mental health
PPE
Prognosis
Public health intervention
Rehabilitation
Service delivery
Transmission
Treatments
Vaccines
Other

Crime and justice

No

Dental

No

Digestive system

No

Ear, nose and throat

No

For peer review only

Education

No

Endocrine and metabolic disorders

No

Eye disorders

No

General interest

No

Genetics

No

Health inequalities/health equity

No

Infections and infestations

Yes

International development

No

Mental health and behavioural conditions

No

Musculoskeletal

No

Neurological

No

Nursing

No

Obstetrics and gynaecology

No

Oral health

No

Palliative care

No

Perioperative care

No

Physiotherapy

No

Pregnancy and childbirth

No

Public health (including social determinants of health)

For peer review only

No

Rehabilitation

No

Respiratory disorders

No

Service delivery

No

Skin disorders

No

Social care

No

Surgery

No

Tropical Medicine

No

Urological

No

Wounds, injuries and accidents

No

Violence and abuse

No

31. Language.

Select each language individually to add it to the list below, use the bin icon to remove any added in error.

English

There is not an English language summary

32. * Country.

Select the country in which the review is being carried out. For multi-national collaborations select all the countries involved.

Netherlands

33. Other registration details.

Name any other organisation where the systematic review title or protocol is registered (e.g. Campbell, or The Joanna Briggs Institute) together with any unique identification number assigned by them. If extracted data will be stored and made available through a repository such as the Systematic Review Data Repository (SRDR), details and a link should be included here. If none, leave blank.

For peer review only

PROSPERO
International prospective register of systematic reviews

National Institute for Health Research

34. Reference and/or URL for published protocol.

If the protocol for this review is published provide details (authors, title and journal details, preferably in Vancouver format)

Add web link to the published protocol.

Or, upload your published protocol here in pdf format. Note that the upload will be publicly accessible.

No I do not make this file publicly available until the review is complete

For peer review only

Please note that the information required in the PROSPERO registration form must be completed in full even if access to a protocol is given.

35. Dissemination plans.

Do you intend to publish the review on completion?

Yes

Give brief details of plans for communicating review findings.?

36. Keywords.

Give words or phrases that best describe the review. Separate keywords with a semicolon or new line. Keywords help PROSPERO users find your review (keywords do not appear in the public record but are included in searches). Be as specific and precise as possible. Avoid acronyms and abbreviations unless these are in wide use.

Systematic review; prognosis; prognostic factor; prediction model; lower respiratory tract infection; adults;

primary care; general practice; hospitalisation; mortality; complications.

37. Details of any existing review of the same topic by the same authors.

If you are registering an update of an existing review give details of the earlier versions and include a full bibliographic reference, if available.

38. * Current review status.

Update review status when the review is completed and when it is published.New registrations must be ongoing so this field is not editable for initial submission.

Please provide anticipated publication date

PROSPERO
International prospective register of systematic reviews

National Institute for Health Research

Review_Completed_not_published

39. Any additional information. [1 change]

Provide any other information relevant to the registration of this review.

Additional information added after initial submission

- Inclusion PICOTS components for review questions (field #15)

- Elaboration upon the assessment of prediction models and how these results help clinicians (GPs) to choose the appropriate tool (if any) for prediction of hospitalization or death in LRTI patients (field #28)

40. Details of final report/publication(s) or preprints if available.

Leave empty until publication details are available OR you have a link to a preprint (NOTE: this field is not editable for initial submission). List authors, title and journal details preferably in Vancouver format.

Give the link to the published review or preprint.

For peer review only
